# Supplementary figures and images for: Quantification of gene expression patterns to reveal the origins of abnormal morphogenesis
Source: eLife. 2018 Sep 20;7:e36405. doi: 10.7554/eLife.36405 (PMC6199133; doi:10.7554/eLife.36405)

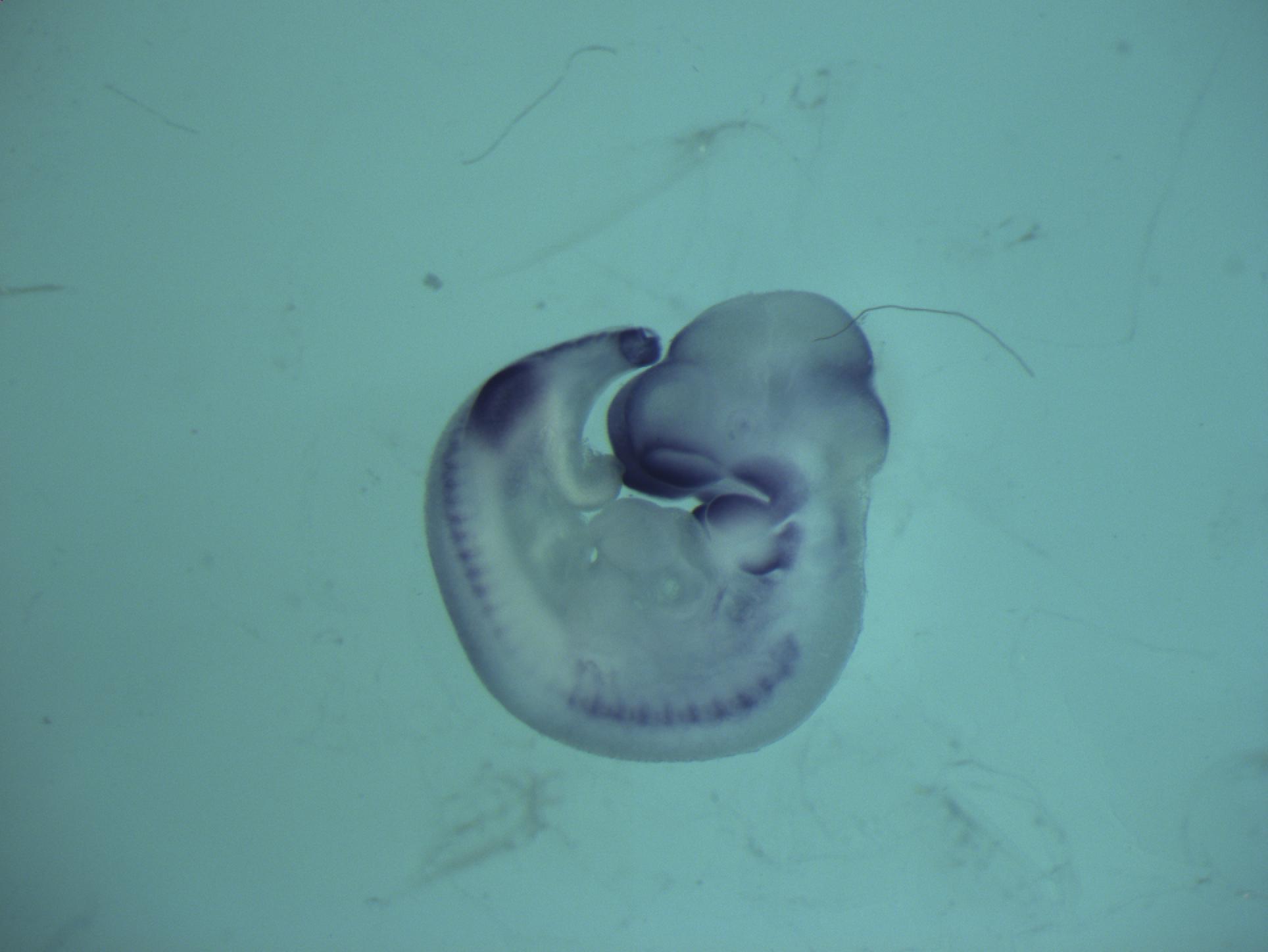

Supplement: Figure 2—source data 1. — This zip archive contains pictures, taken using a Leica MX16F microscope, of the right and left sides of the mouse embryos that underwent Dusp6 WMISH. Folders are organized by developmental stage and genotype. [file elife-36405-fig2-data1.zip › Figure 2 supplement 1-Source data 1/Dusp6 10.5 mut/Dusp6 10.5 mut1L.jpg]

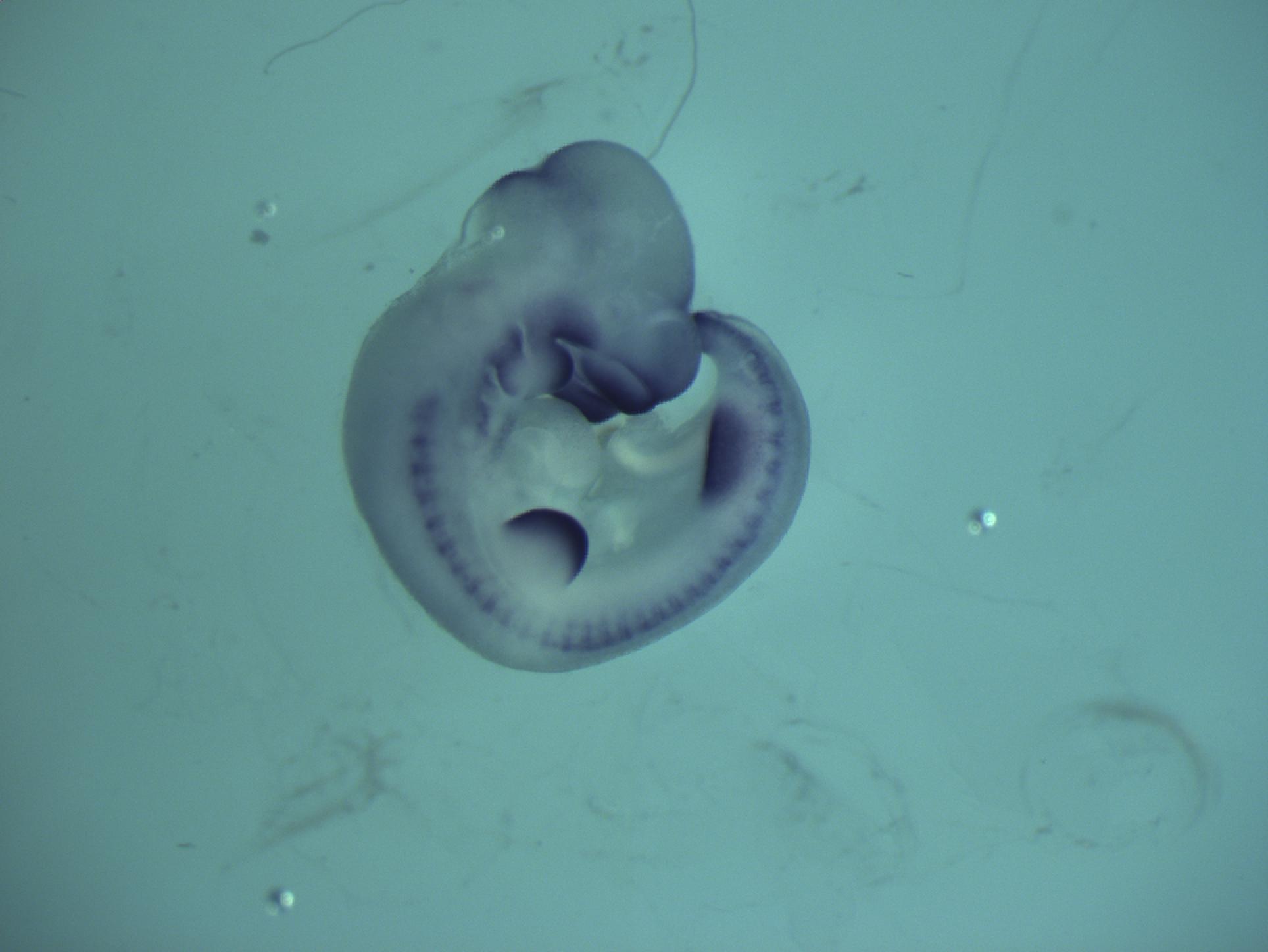

Supplement: Figure 2—source data 1. — This zip archive contains pictures, taken using a Leica MX16F microscope, of the right and left sides of the mouse embryos that underwent Dusp6 WMISH. Folders are organized by developmental stage and genotype. [file elife-36405-fig2-data1.zip › Figure 2 supplement 1-Source data 1/Dusp6 10.5 mut/Dusp6 10.5 mut1R.jpg]

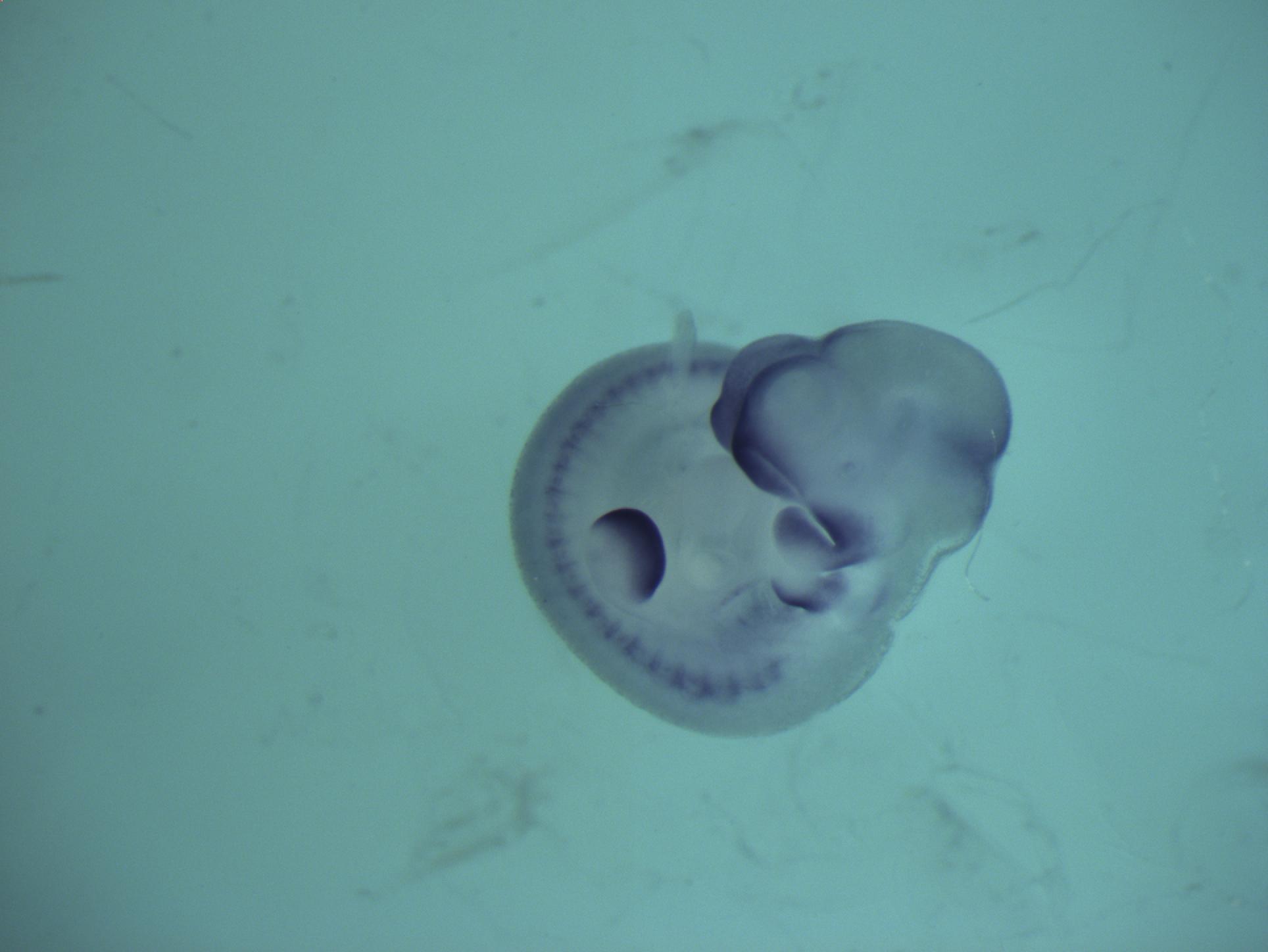

Supplement: Figure 2—source data 1. — This zip archive contains pictures, taken using a Leica MX16F microscope, of the right and left sides of the mouse embryos that underwent Dusp6 WMISH. Folders are organized by developmental stage and genotype. [file elife-36405-fig2-data1.zip › Figure 2 supplement 1-Source data 1/Dusp6 10.5 mut/Dusp6 10.5 mut2L.jpg]

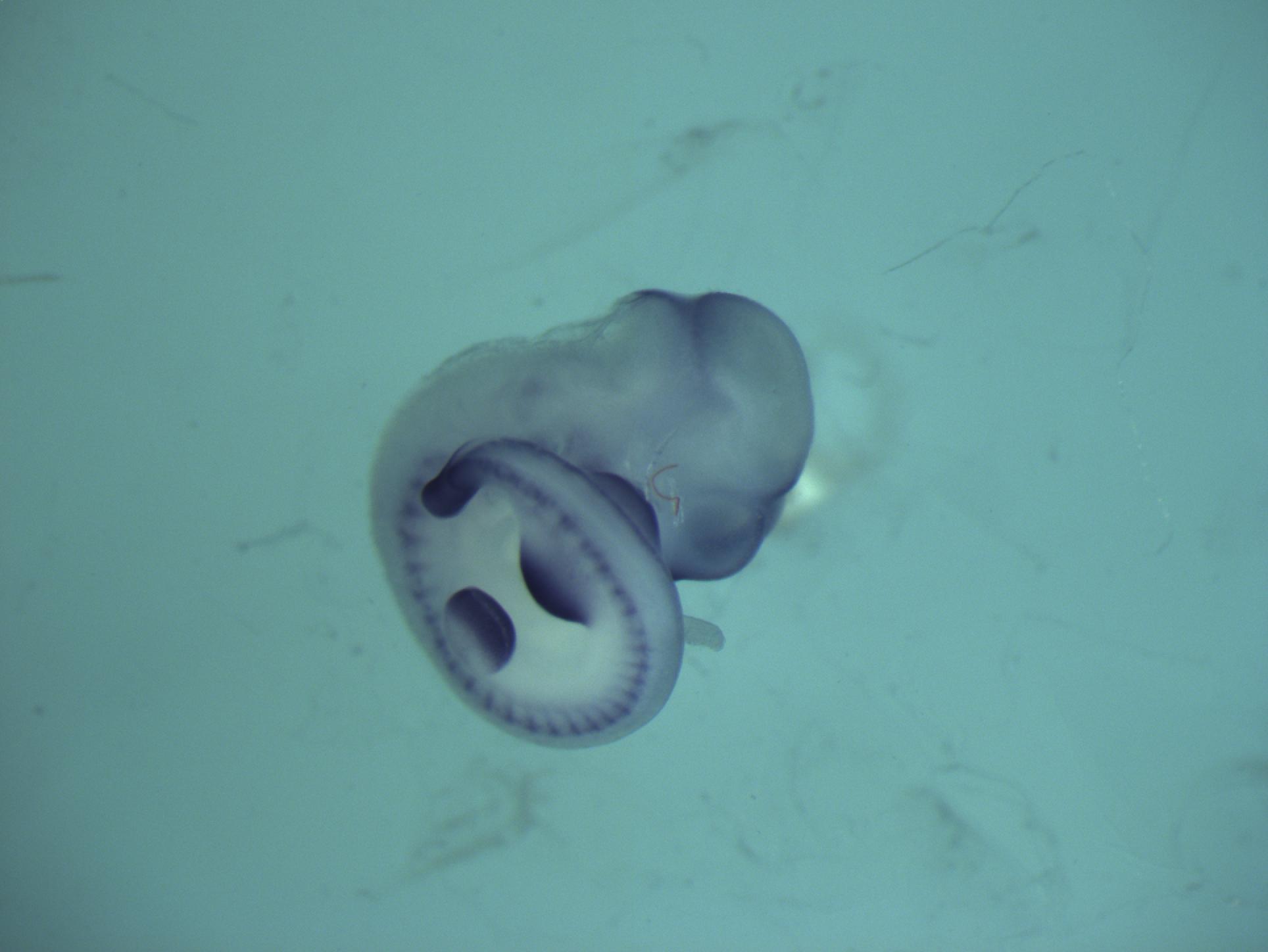

Supplement: Figure 2—source data 1. — This zip archive contains pictures, taken using a Leica MX16F microscope, of the right and left sides of the mouse embryos that underwent Dusp6 WMISH. Folders are organized by developmental stage and genotype. [file elife-36405-fig2-data1.zip › Figure 2 supplement 1-Source data 1/Dusp6 10.5 mut/Dusp6 10.5 mut2R.jpg]

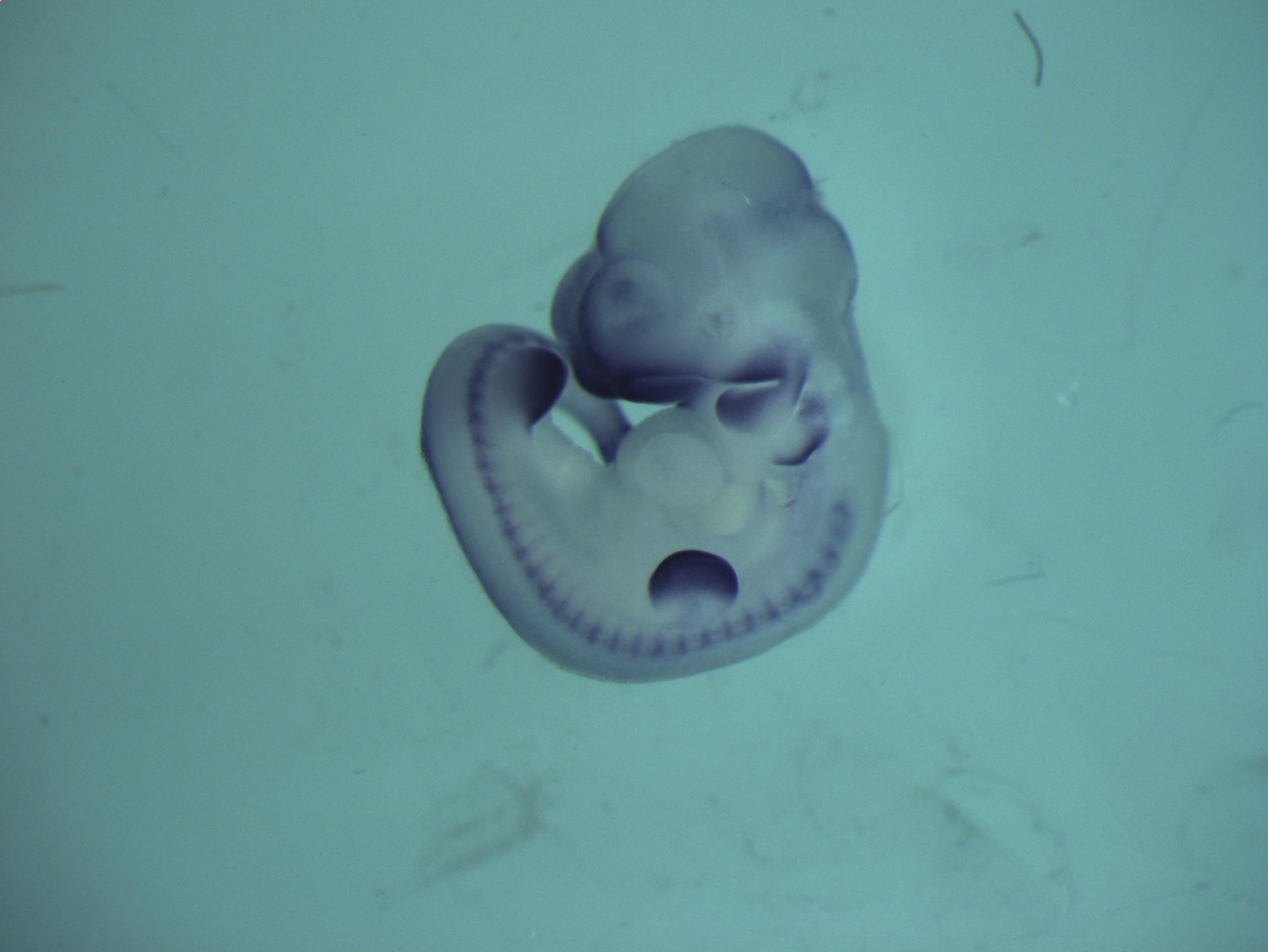

Supplement: Figure 2—source data 1. — This zip archive contains pictures, taken using a Leica MX16F microscope, of the right and left sides of the mouse embryos that underwent Dusp6 WMISH. Folders are organized by developmental stage and genotype. [file elife-36405-fig2-data1.zip › Figure 2 supplement 1-Source data 1/Dusp6 10.5 mut/Dusp6 10.5 mut3L.jpg]

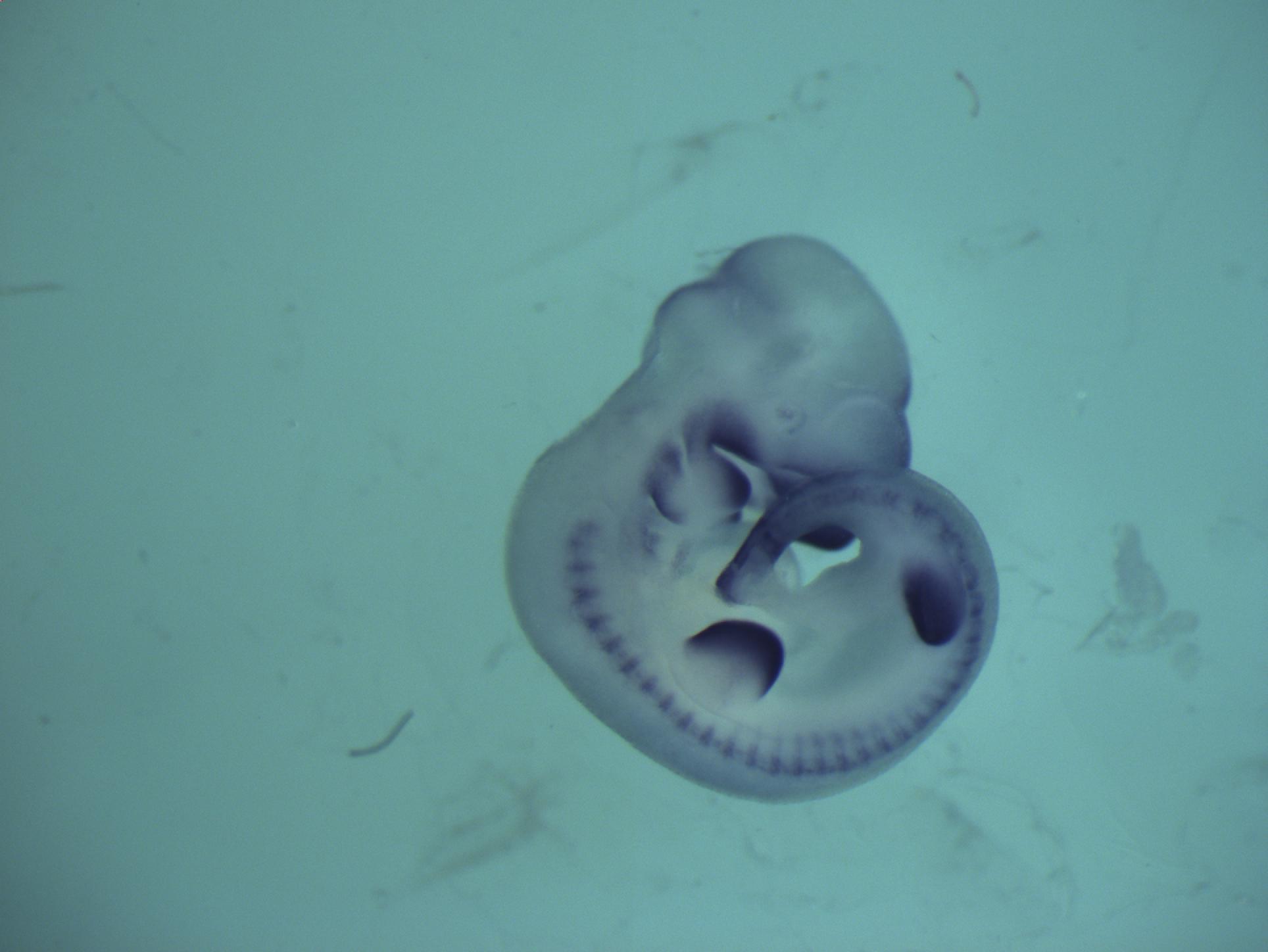

Supplement: Figure 2—source data 1. — This zip archive contains pictures, taken using a Leica MX16F microscope, of the right and left sides of the mouse embryos that underwent Dusp6 WMISH. Folders are organized by developmental stage and genotype. [file elife-36405-fig2-data1.zip › Figure 2 supplement 1-Source data 1/Dusp6 10.5 mut/Dusp6 10.5 mut3R.jpg]

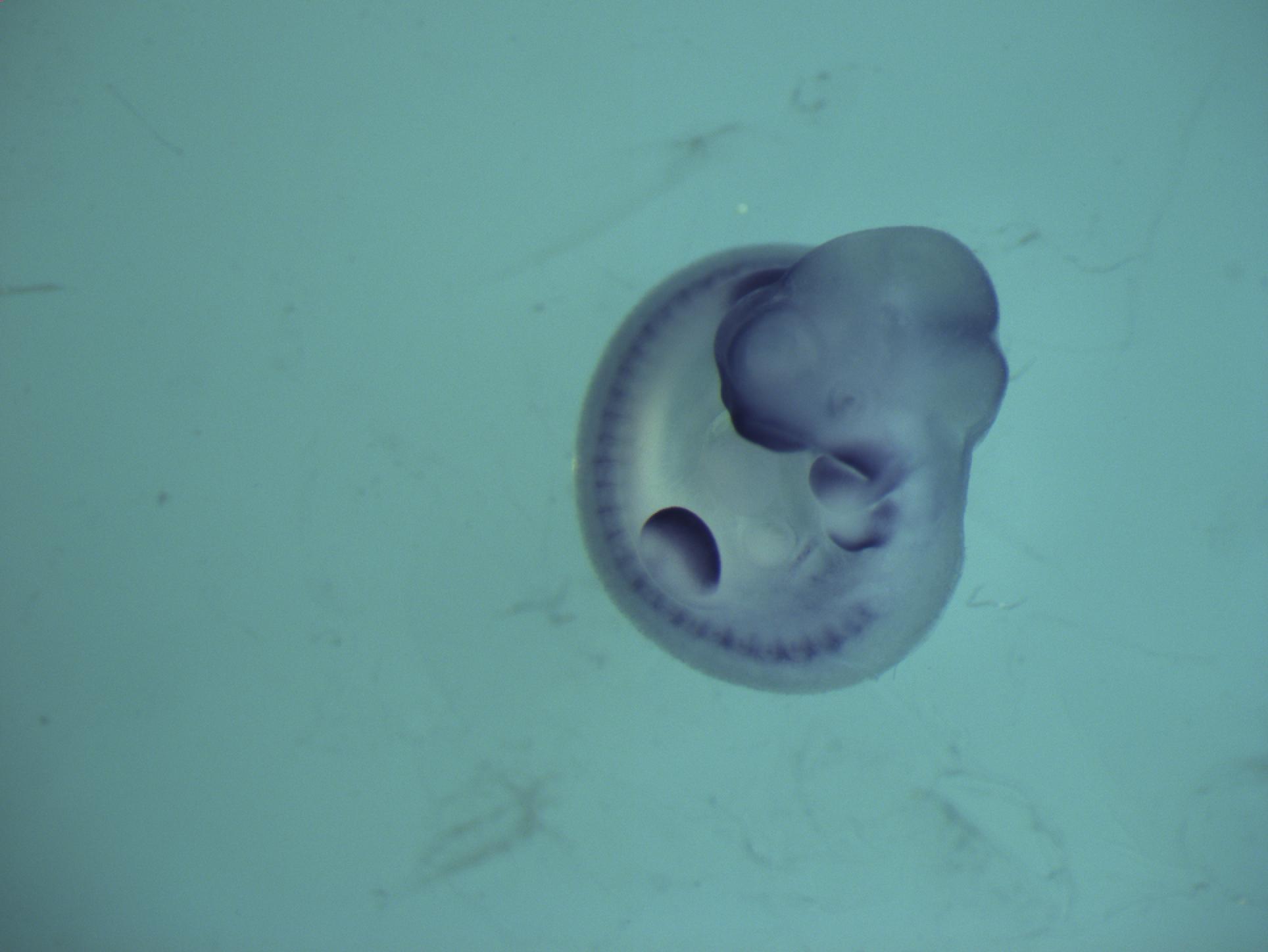

Supplement: Figure 2—source data 1. — This zip archive contains pictures, taken using a Leica MX16F microscope, of the right and left sides of the mouse embryos that underwent Dusp6 WMISH. Folders are organized by developmental stage and genotype. [file elife-36405-fig2-data1.zip › Figure 2 supplement 1-Source data 1/Dusp6 10.5 mut/Dusp6 10.5 mut4L.jpg]

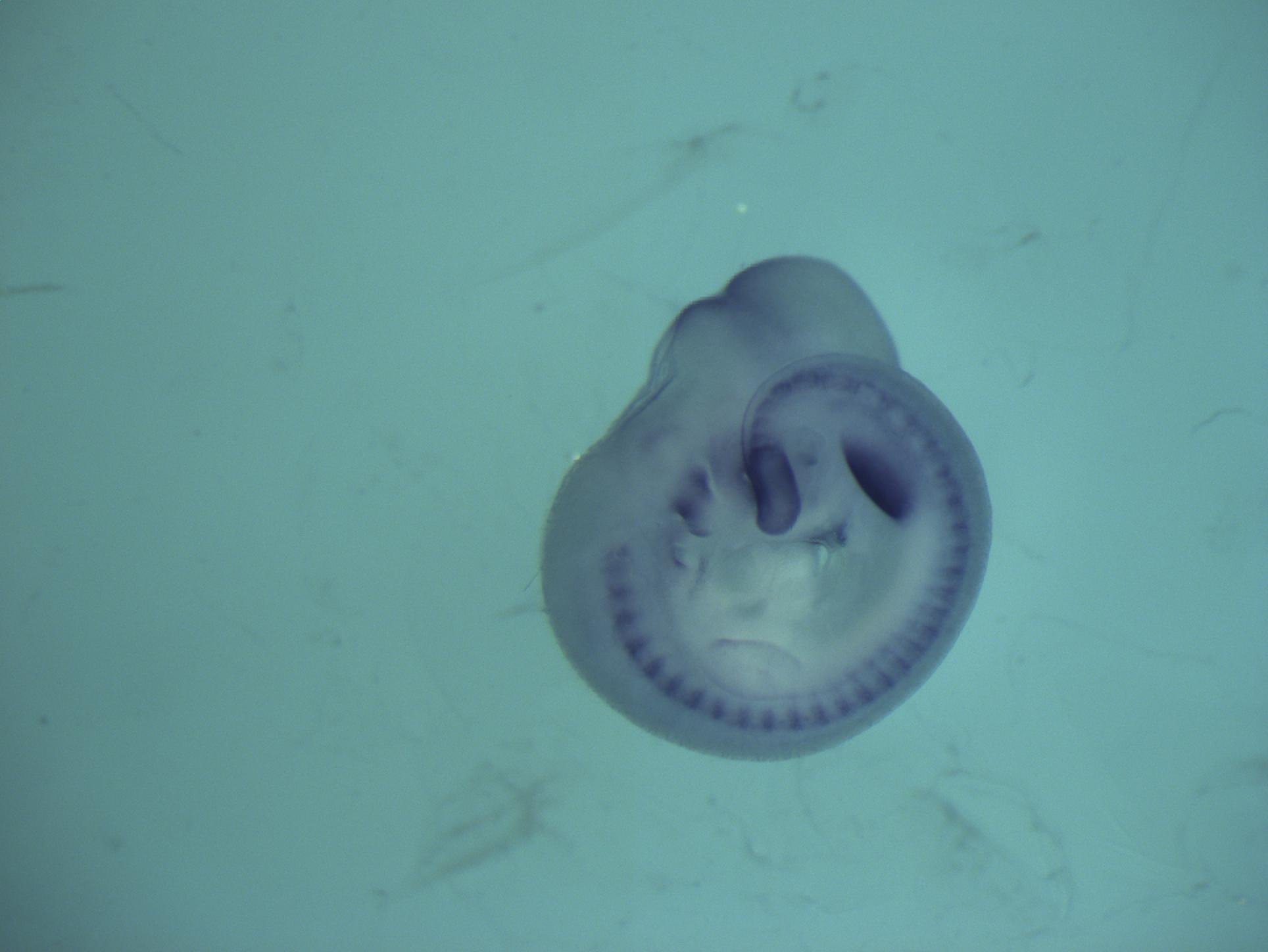

Supplement: Figure 2—source data 1. — This zip archive contains pictures, taken using a Leica MX16F microscope, of the right and left sides of the mouse embryos that underwent Dusp6 WMISH. Folders are organized by developmental stage and genotype. [file elife-36405-fig2-data1.zip › Figure 2 supplement 1-Source data 1/Dusp6 10.5 mut/Dusp6 10.5 mut4R.jpg]

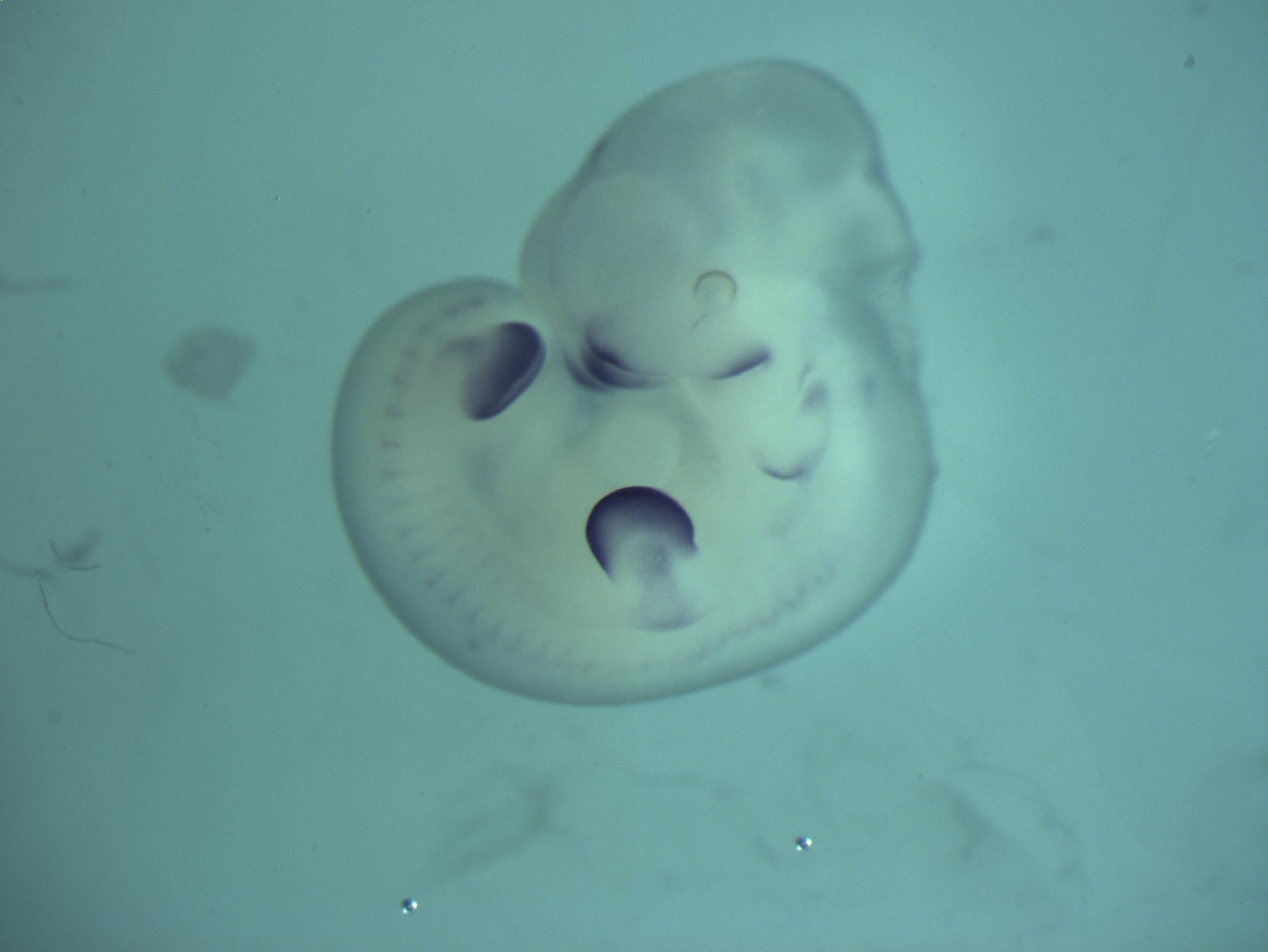

Supplement: Figure 2—source data 1. — This zip archive contains pictures, taken using a Leica MX16F microscope, of the right and left sides of the mouse embryos that underwent Dusp6 WMISH. Folders are organized by developmental stage and genotype. [file elife-36405-fig2-data1.zip › Figure 2 supplement 1-Source data 1/Dusp6 10.5 mut/Dusp6 10.5 mut5L.jpg]

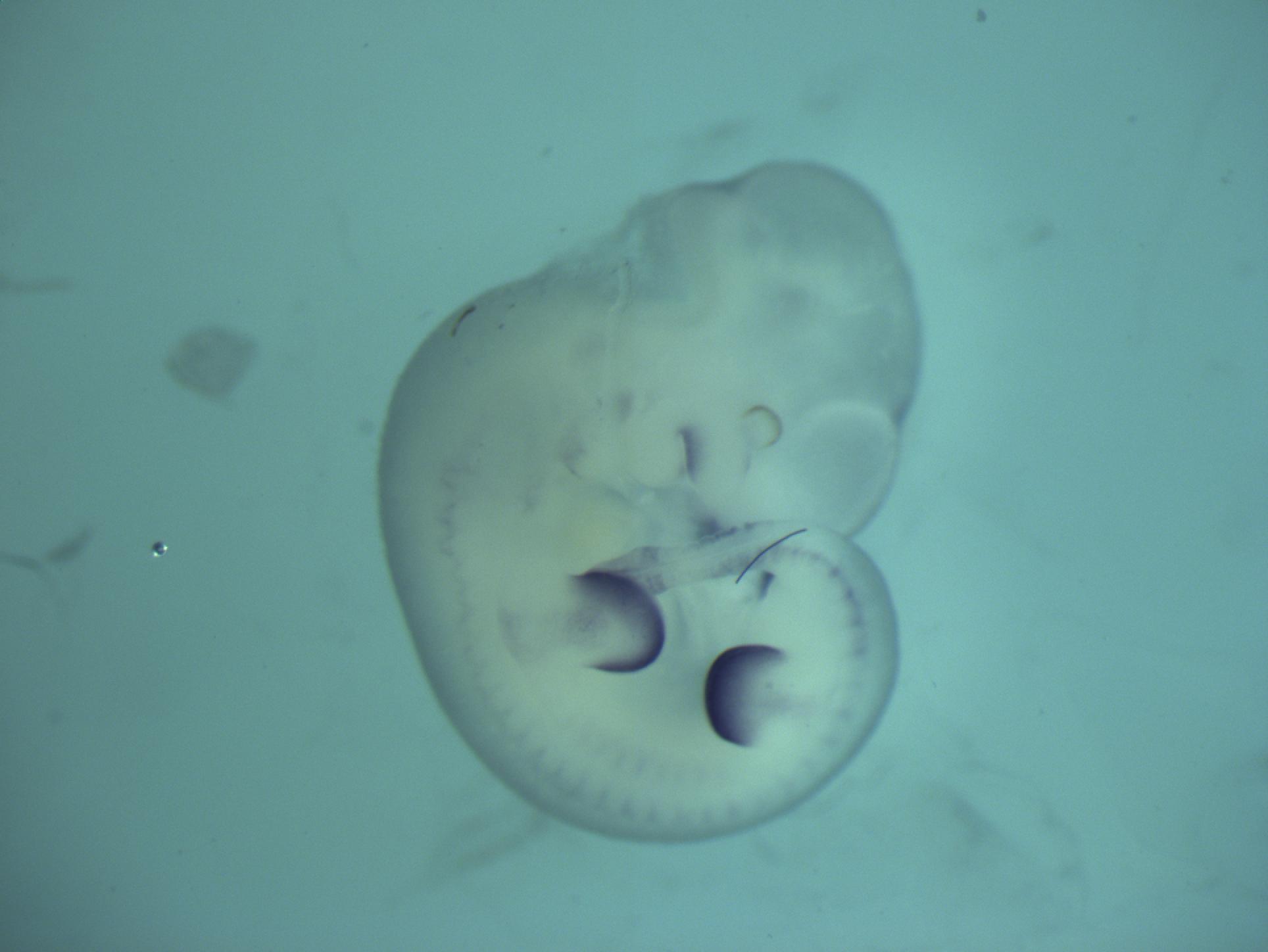

Supplement: Figure 2—source data 1. — This zip archive contains pictures, taken using a Leica MX16F microscope, of the right and left sides of the mouse embryos that underwent Dusp6 WMISH. Folders are organized by developmental stage and genotype. [file elife-36405-fig2-data1.zip › Figure 2 supplement 1-Source data 1/Dusp6 10.5 mut/Dusp6 10.5 mut5R.jpg]

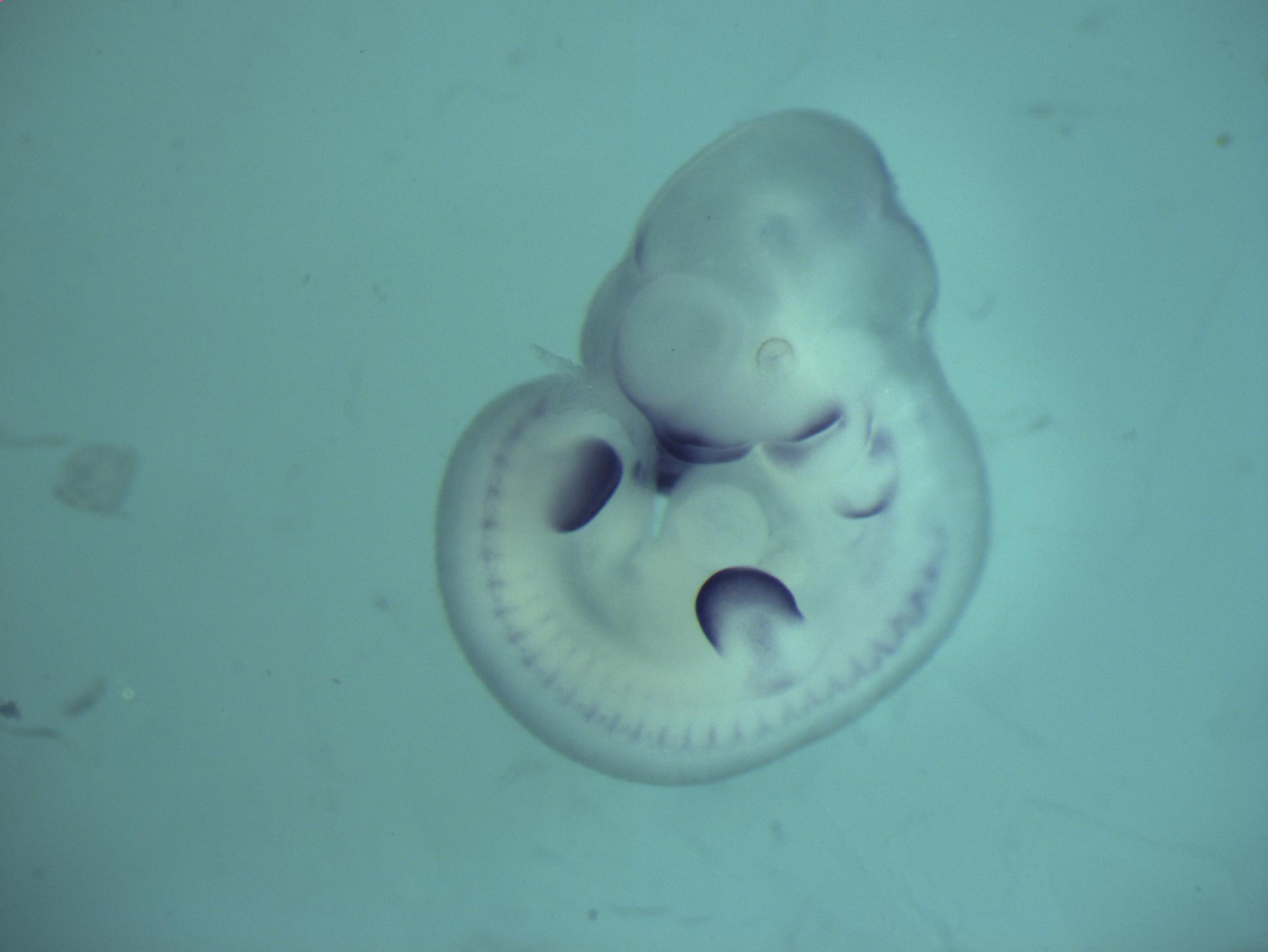

Supplement: Figure 2—source data 1. — This zip archive contains pictures, taken using a Leica MX16F microscope, of the right and left sides of the mouse embryos that underwent Dusp6 WMISH. Folders are organized by developmental stage and genotype. [file elife-36405-fig2-data1.zip › Figure 2 supplement 1-Source data 1/Dusp6 10.5 mut/Dusp6 10.5 mut6L.jpg]

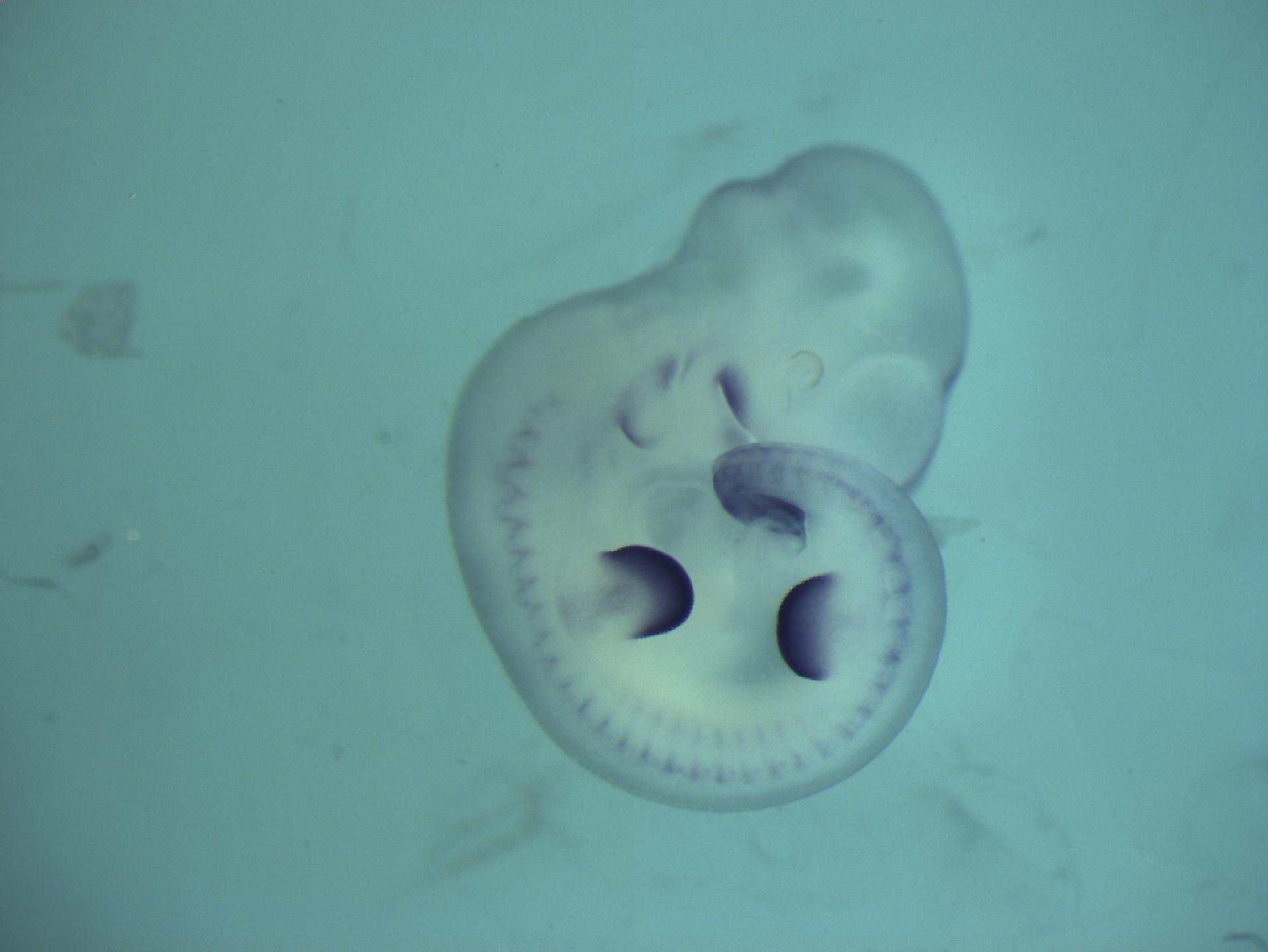

Supplement: Figure 2—source data 1. — This zip archive contains pictures, taken using a Leica MX16F microscope, of the right and left sides of the mouse embryos that underwent Dusp6 WMISH. Folders are organized by developmental stage and genotype. [file elife-36405-fig2-data1.zip › Figure 2 supplement 1-Source data 1/Dusp6 10.5 mut/Dusp6 10.5 mut6R.jpg]

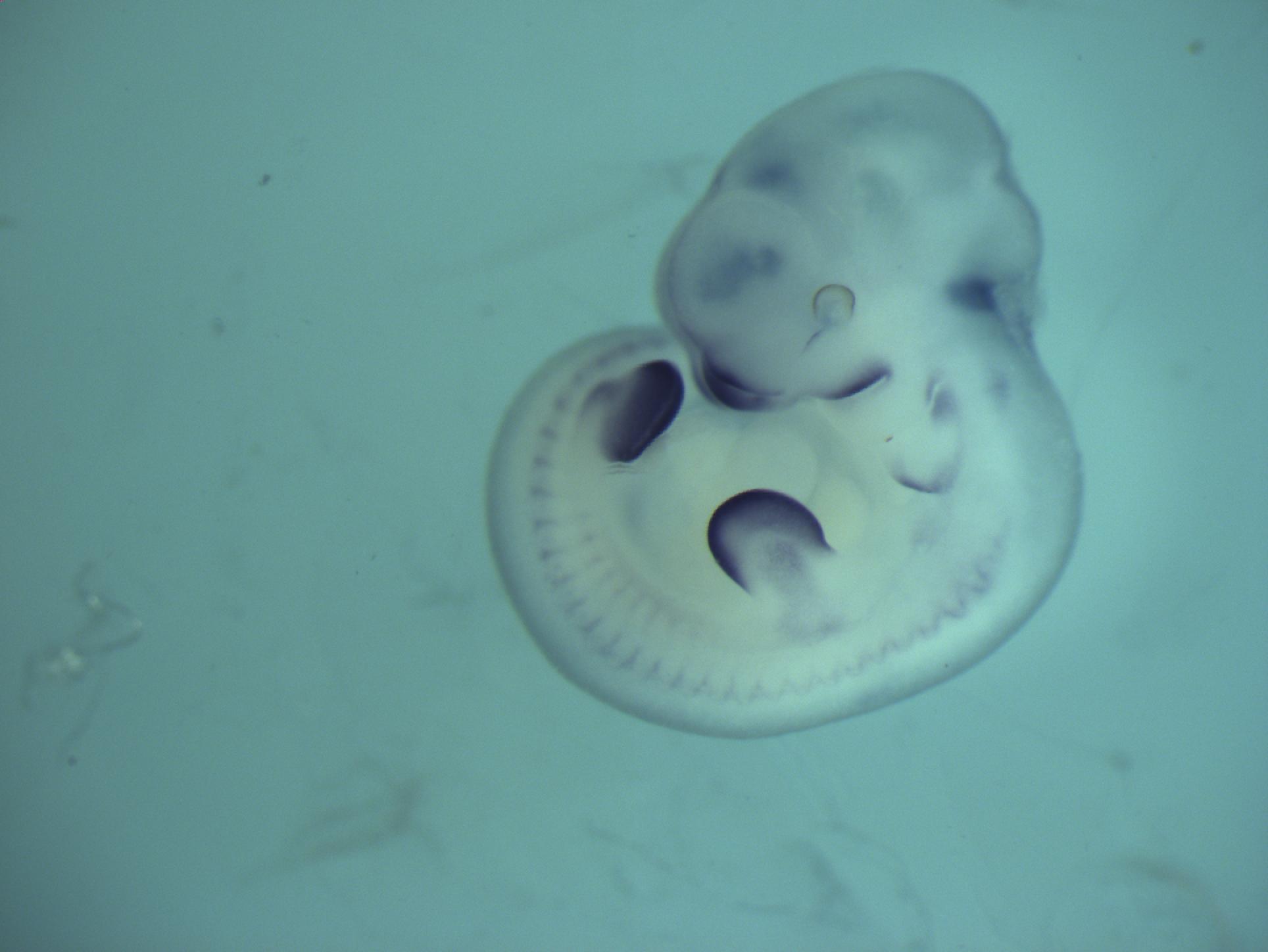

Supplement: Figure 2—source data 1. — This zip archive contains pictures, taken using a Leica MX16F microscope, of the right and left sides of the mouse embryos that underwent Dusp6 WMISH. Folders are organized by developmental stage and genotype. [file elife-36405-fig2-data1.zip › Figure 2 supplement 1-Source data 1/Dusp6 10.5 mut/Dusp6 10.5 mut7L.jpg]

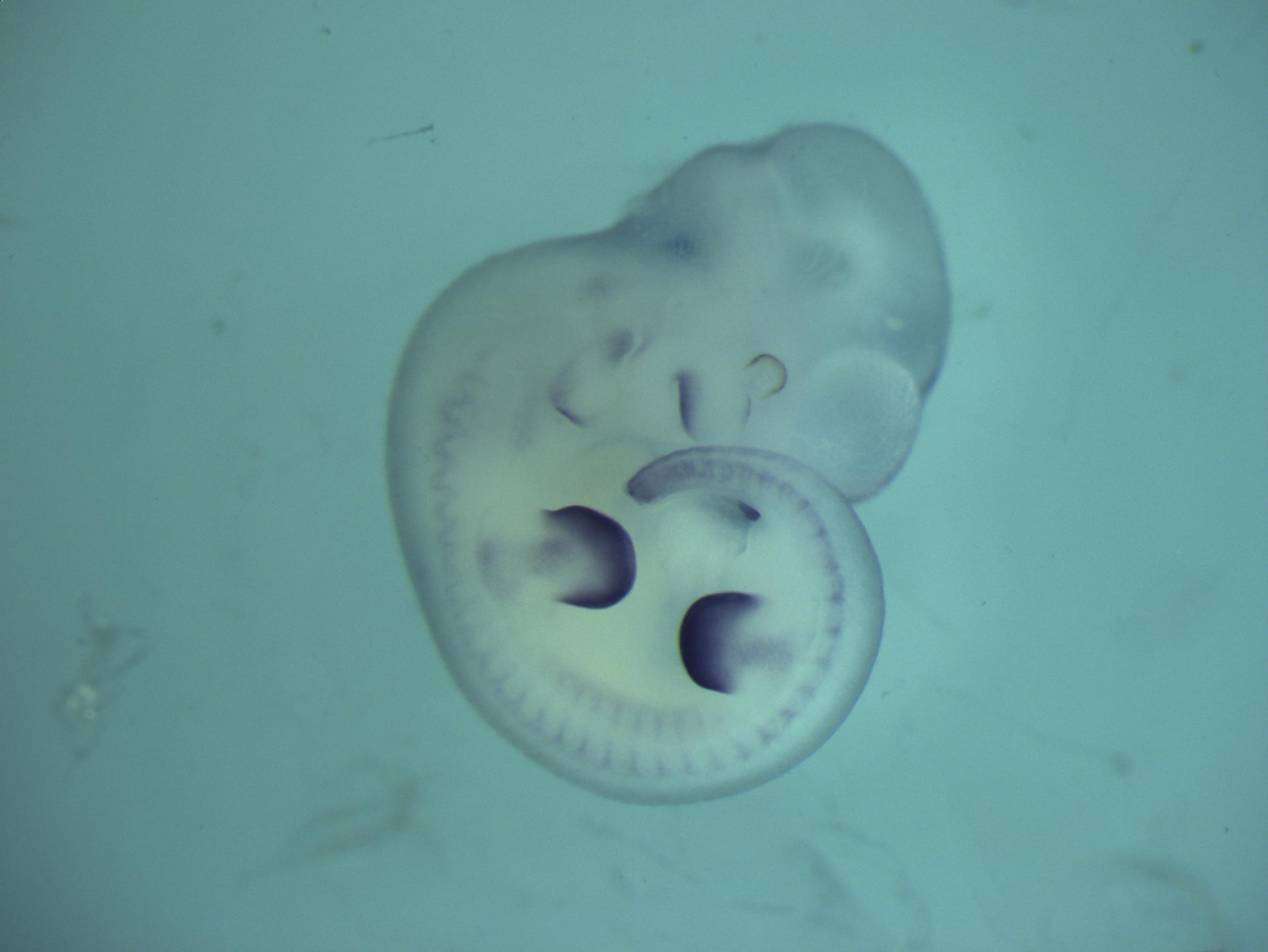

Supplement: Figure 2—source data 1. — This zip archive contains pictures, taken using a Leica MX16F microscope, of the right and left sides of the mouse embryos that underwent Dusp6 WMISH. Folders are organized by developmental stage and genotype. [file elife-36405-fig2-data1.zip › Figure 2 supplement 1-Source data 1/Dusp6 10.5 mut/Dusp6 10.5 mut7R.jpg]

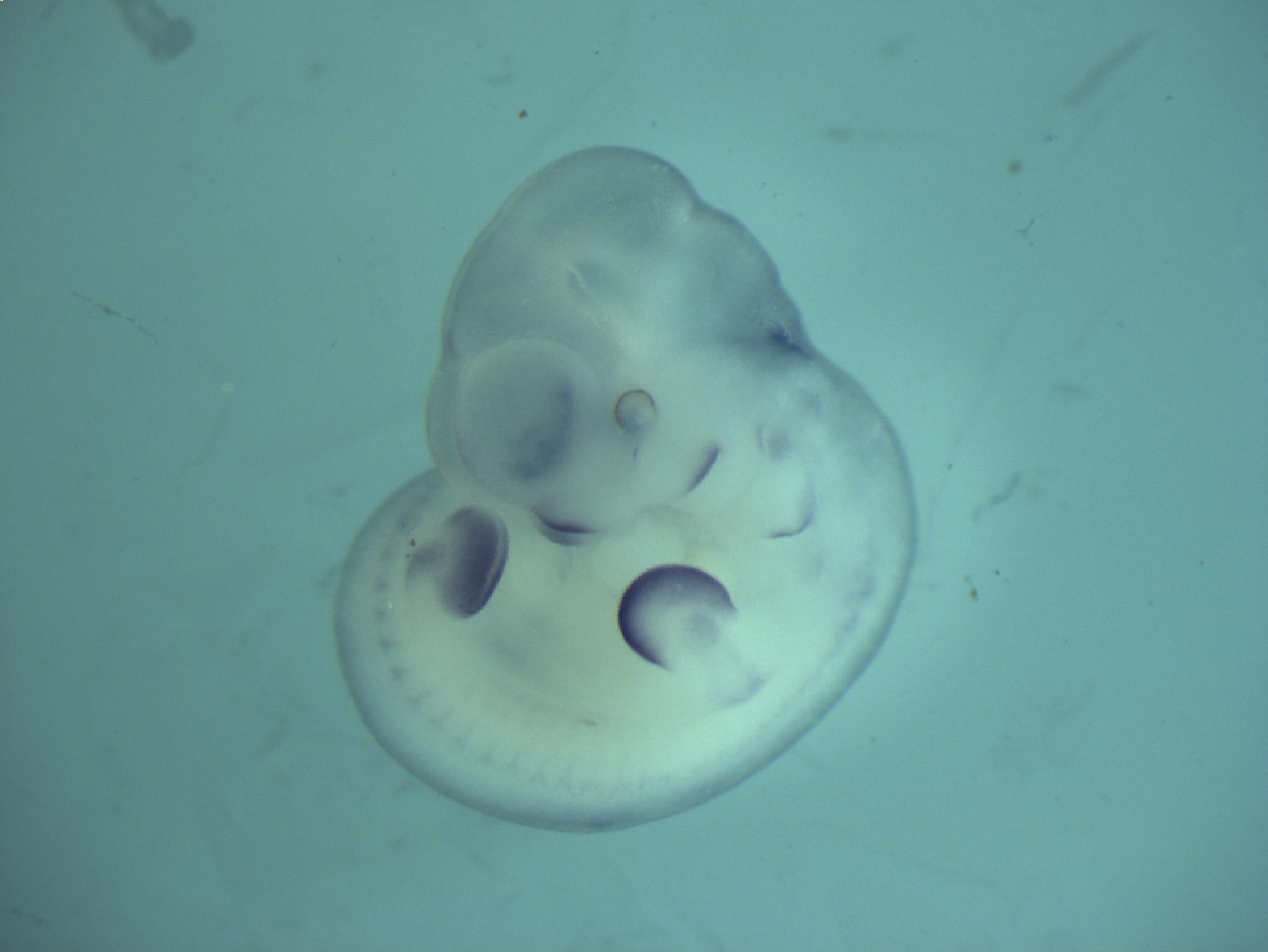

Supplement: Figure 2—source data 1. — This zip archive contains pictures, taken using a Leica MX16F microscope, of the right and left sides of the mouse embryos that underwent Dusp6 WMISH. Folders are organized by developmental stage and genotype. [file elife-36405-fig2-data1.zip › Figure 2 supplement 1-Source data 1/Dusp6 10.5 mut/Dusp6 10.5 mut8L.jpg]

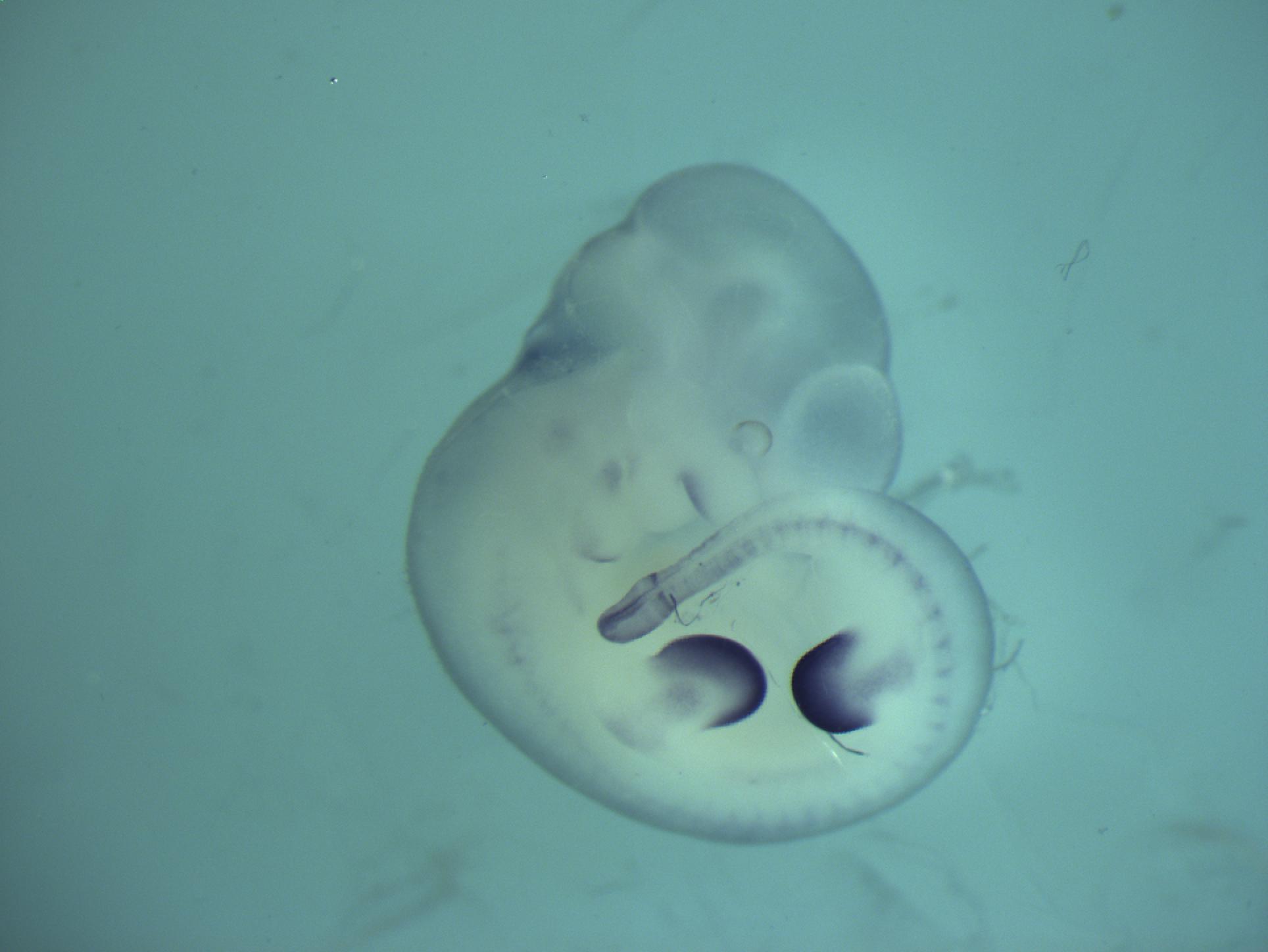

Supplement: Figure 2—source data 1. — This zip archive contains pictures, taken using a Leica MX16F microscope, of the right and left sides of the mouse embryos that underwent Dusp6 WMISH. Folders are organized by developmental stage and genotype. [file elife-36405-fig2-data1.zip › Figure 2 supplement 1-Source data 1/Dusp6 10.5 mut/Dusp6 10.5 mut8R.jpg]

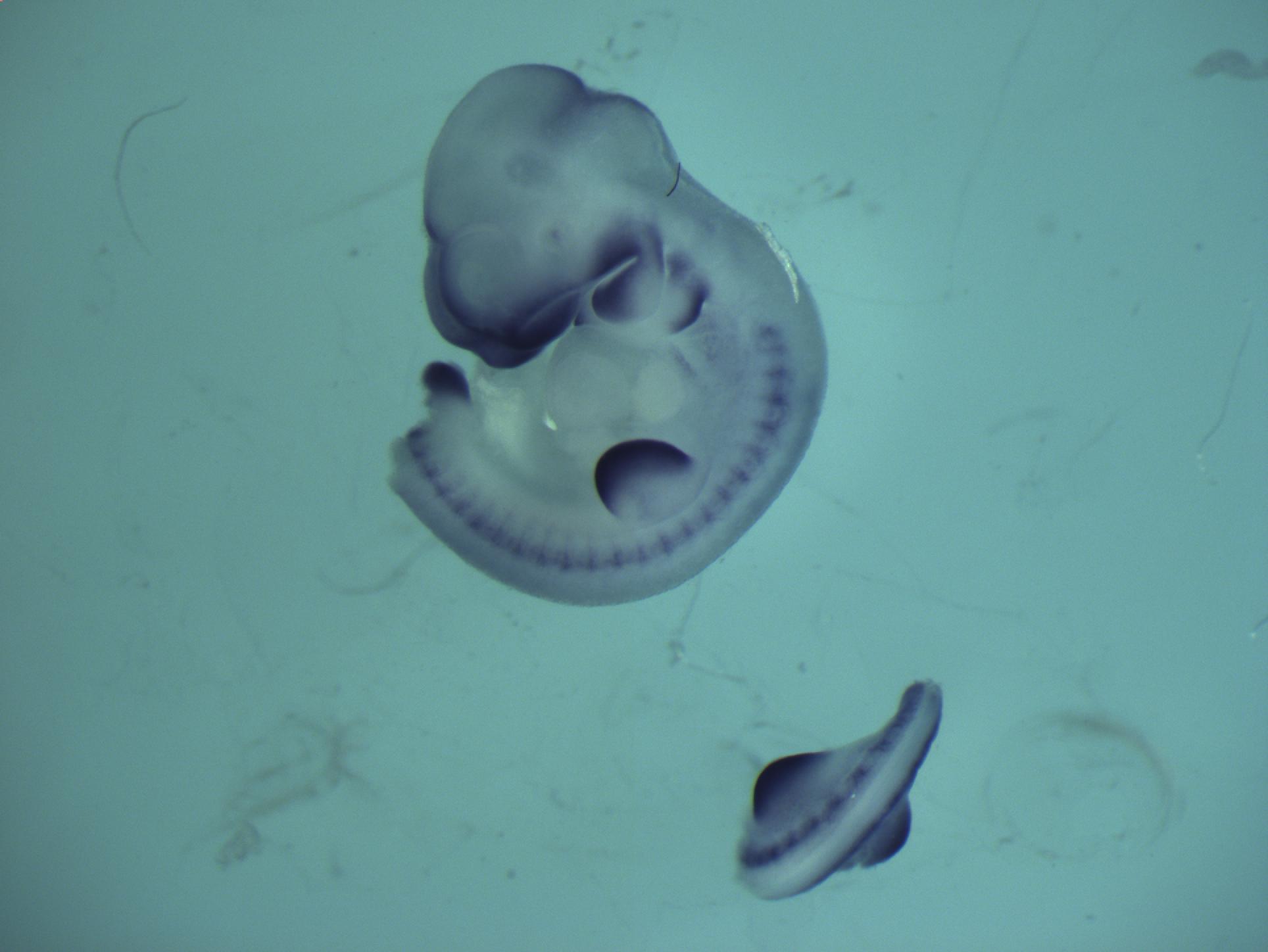

Supplement: Figure 2—source data 1. — This zip archive contains pictures, taken using a Leica MX16F microscope, of the right and left sides of the mouse embryos that underwent Dusp6 WMISH. Folders are organized by developmental stage and genotype. [file elife-36405-fig2-data1.zip › Figure 2 supplement 1-Source data 1/Dusp6 10.5 wt/Dusp6 10.5 wt10L.jpg]

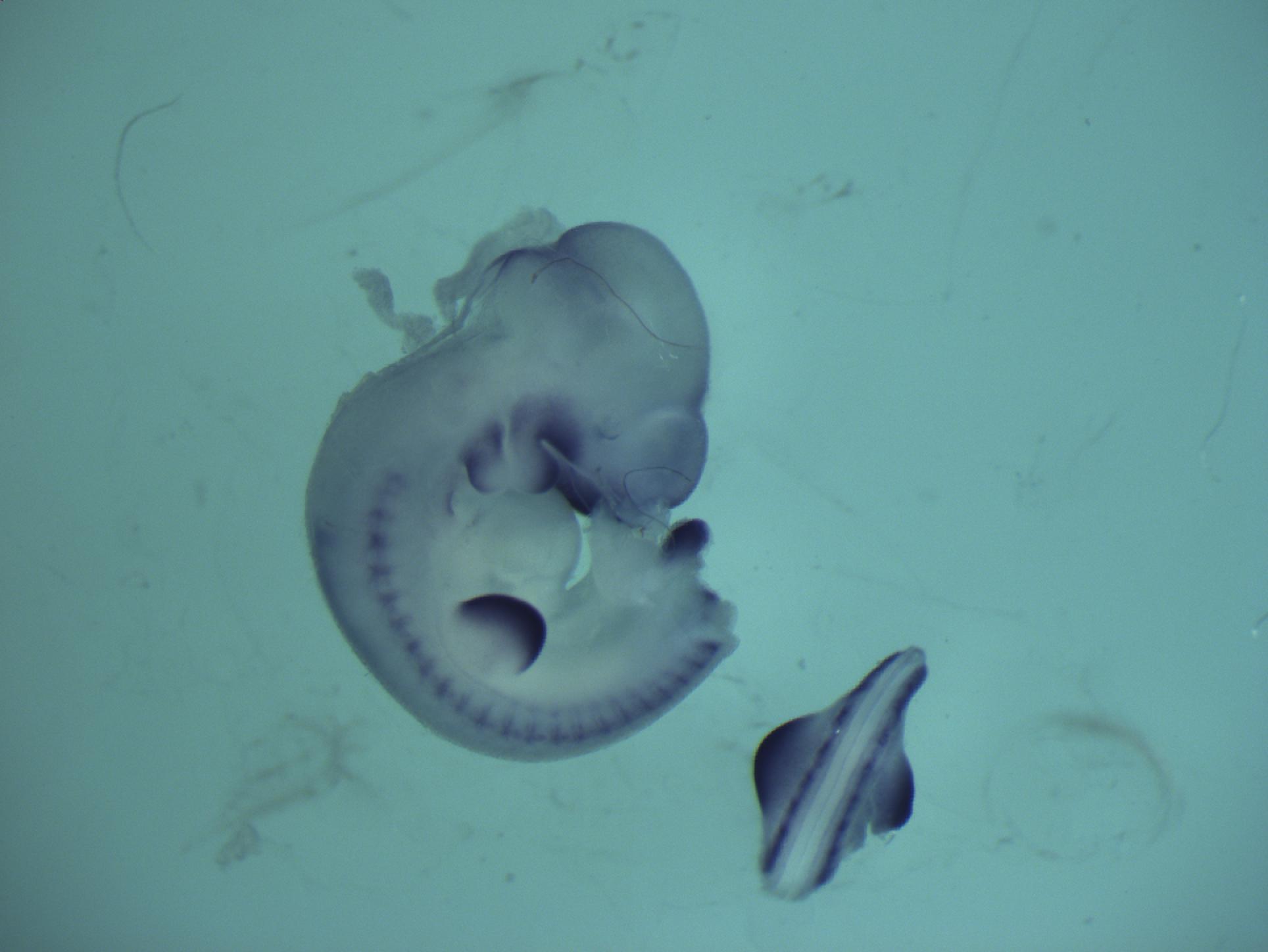

Supplement: Figure 2—source data 1. — This zip archive contains pictures, taken using a Leica MX16F microscope, of the right and left sides of the mouse embryos that underwent Dusp6 WMISH. Folders are organized by developmental stage and genotype. [file elife-36405-fig2-data1.zip › Figure 2 supplement 1-Source data 1/Dusp6 10.5 wt/Dusp6 10.5 wt10R.jpg]

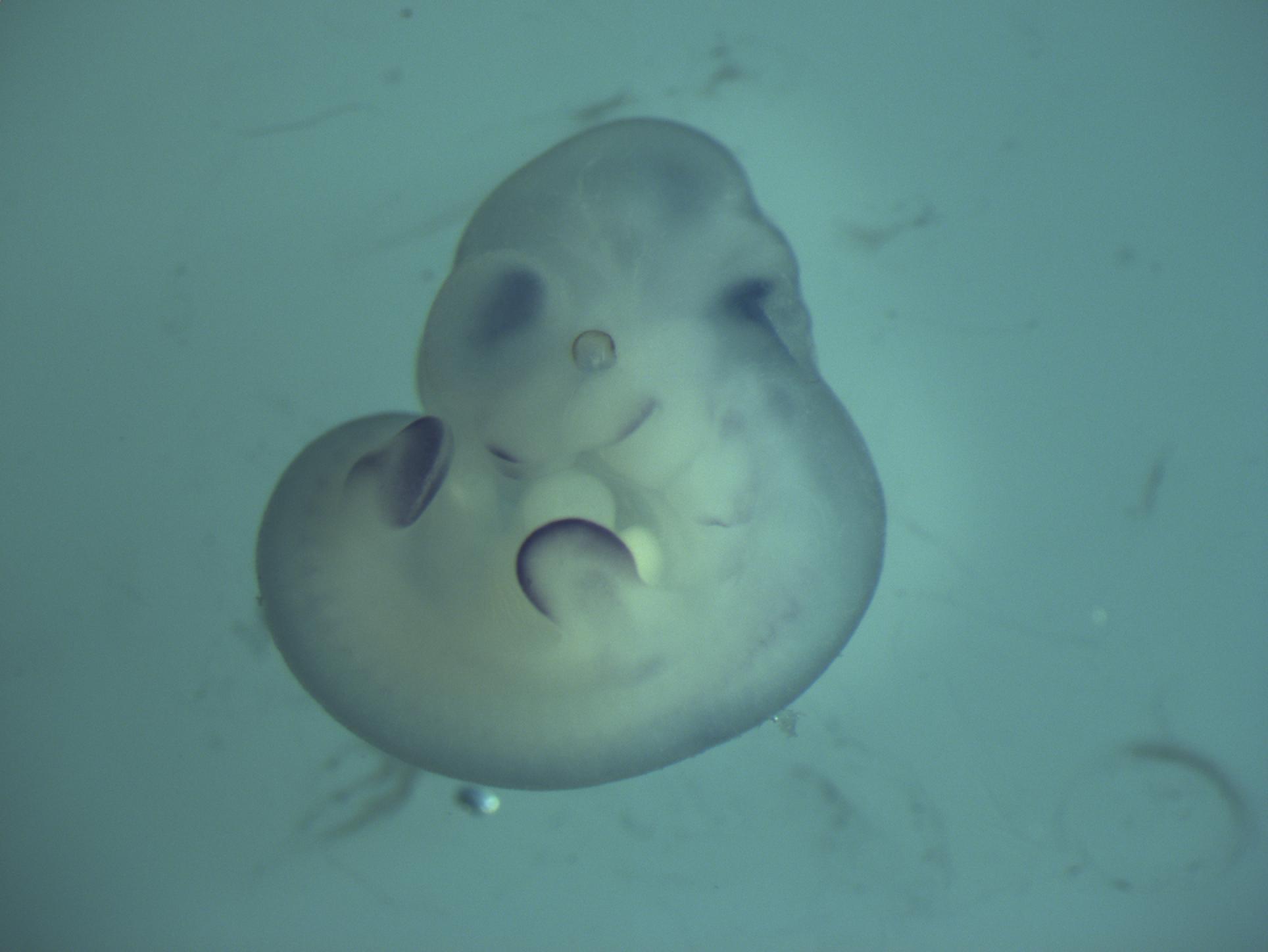

Supplement: Figure 2—source data 1. — This zip archive contains pictures, taken using a Leica MX16F microscope, of the right and left sides of the mouse embryos that underwent Dusp6 WMISH. Folders are organized by developmental stage and genotype. [file elife-36405-fig2-data1.zip › Figure 2 supplement 1-Source data 1/Dusp6 10.5 wt/Dusp6 10.5 wt1L.jpg]

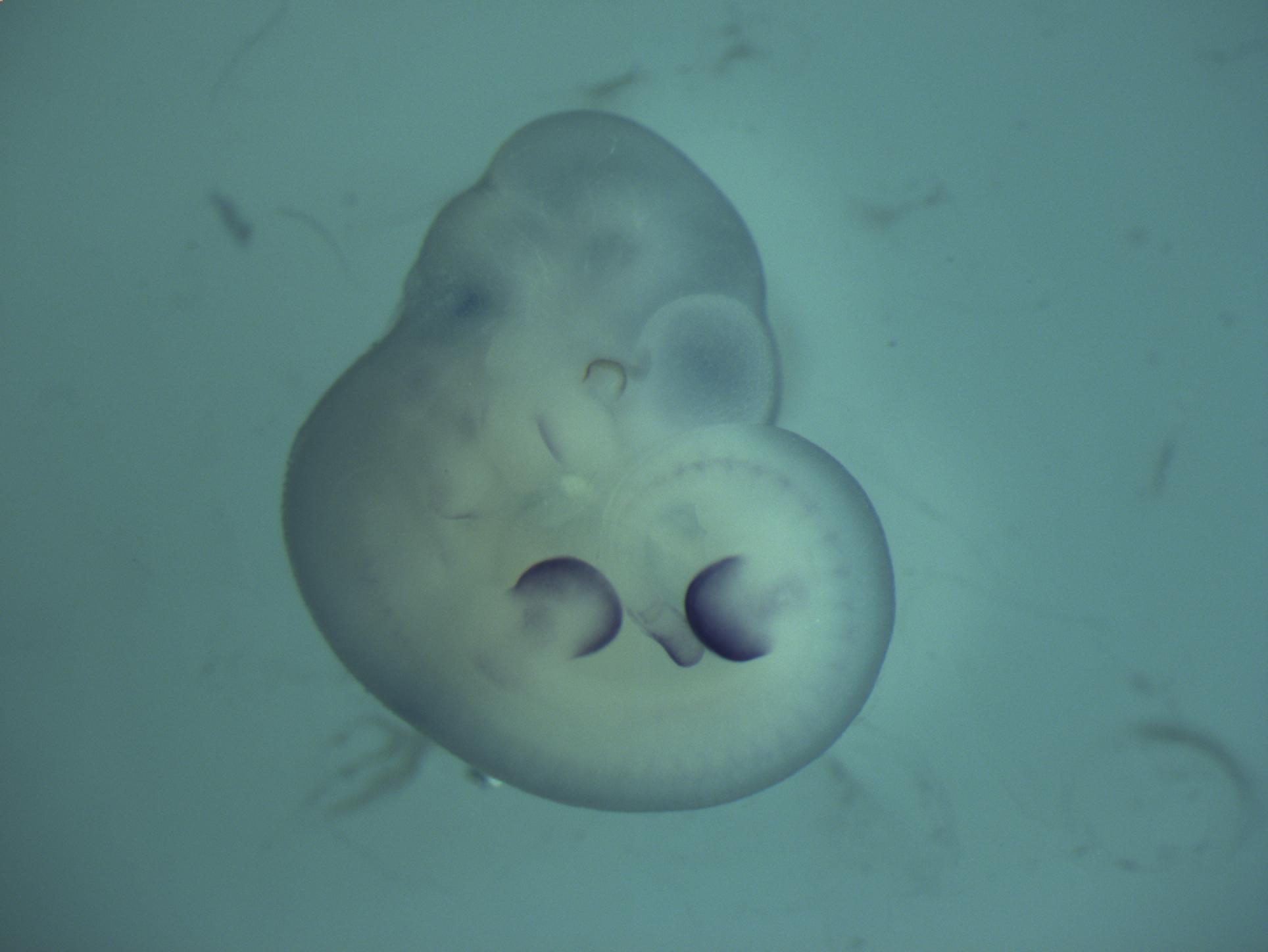

Supplement: Figure 2—source data 1. — This zip archive contains pictures, taken using a Leica MX16F microscope, of the right and left sides of the mouse embryos that underwent Dusp6 WMISH. Folders are organized by developmental stage and genotype. [file elife-36405-fig2-data1.zip › Figure 2 supplement 1-Source data 1/Dusp6 10.5 wt/Dusp6 10.5 wt1R.jpg]

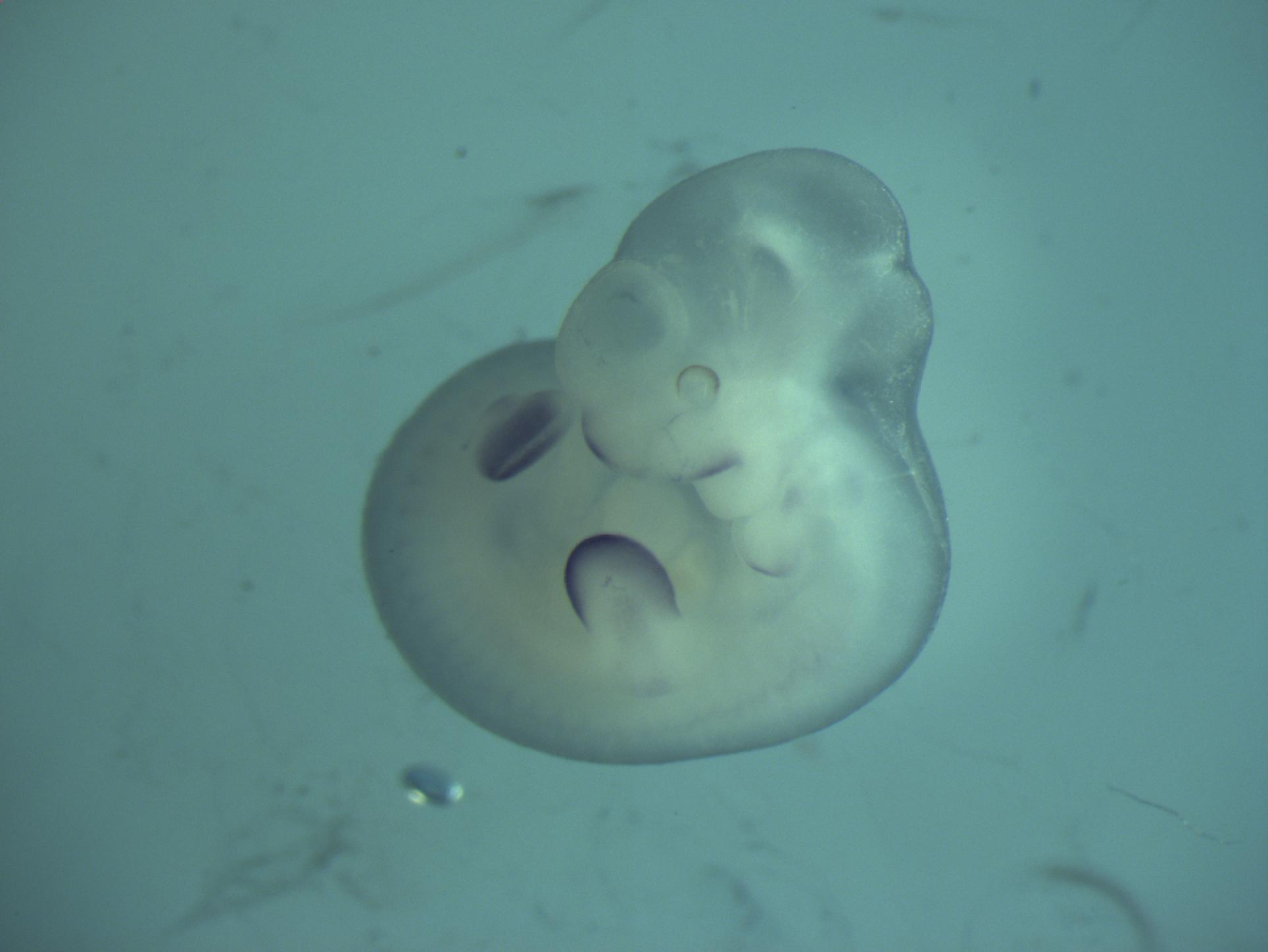

Supplement: Figure 2—source data 1. — This zip archive contains pictures, taken using a Leica MX16F microscope, of the right and left sides of the mouse embryos that underwent Dusp6 WMISH. Folders are organized by developmental stage and genotype. [file elife-36405-fig2-data1.zip › Figure 2 supplement 1-Source data 1/Dusp6 10.5 wt/Dusp6 10.5 wt2L.jpg]

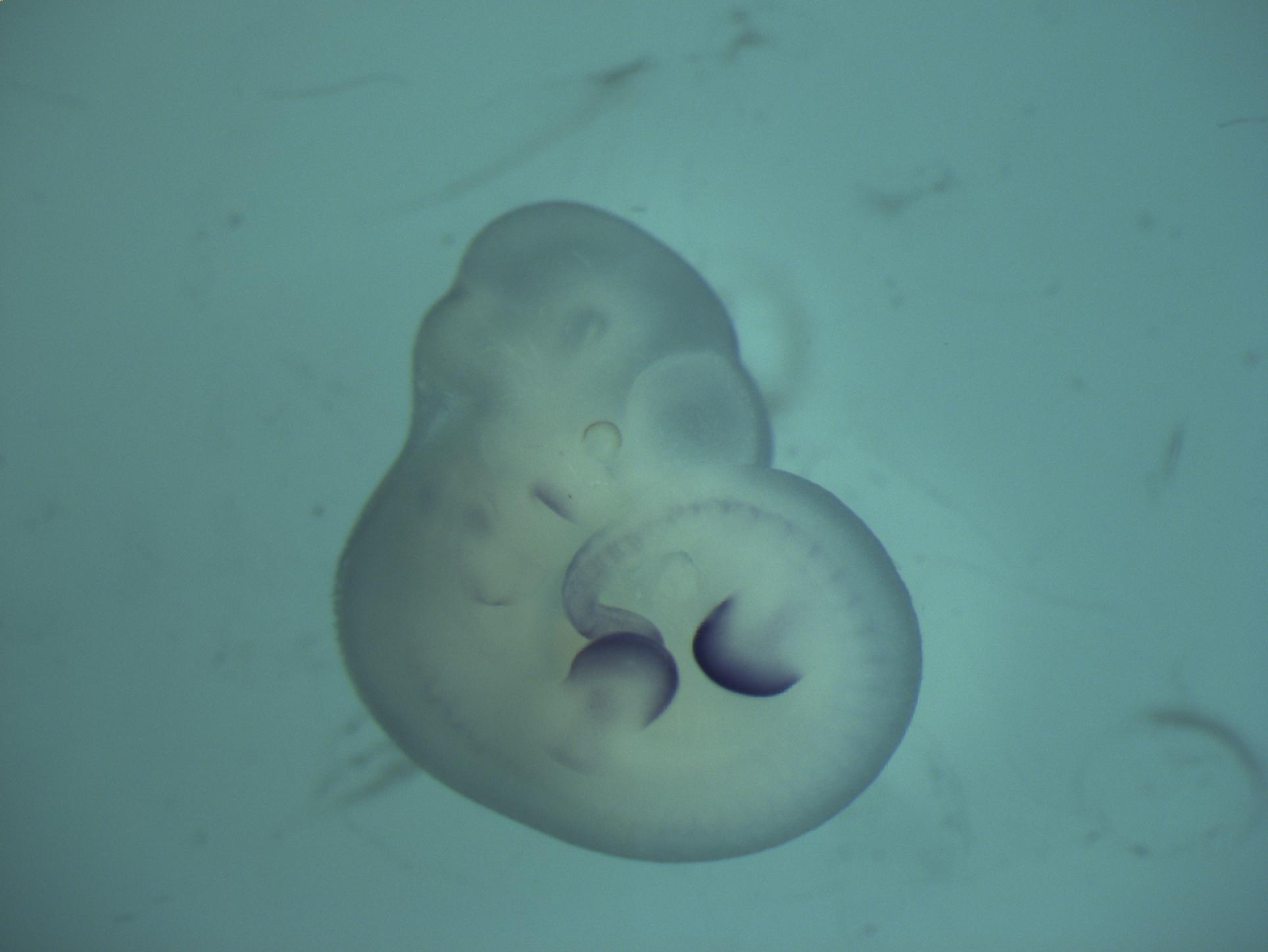

Supplement: Figure 2—source data 1. — This zip archive contains pictures, taken using a Leica MX16F microscope, of the right and left sides of the mouse embryos that underwent Dusp6 WMISH. Folders are organized by developmental stage and genotype. [file elife-36405-fig2-data1.zip › Figure 2 supplement 1-Source data 1/Dusp6 10.5 wt/Dusp6 10.5 wt2R.jpg]

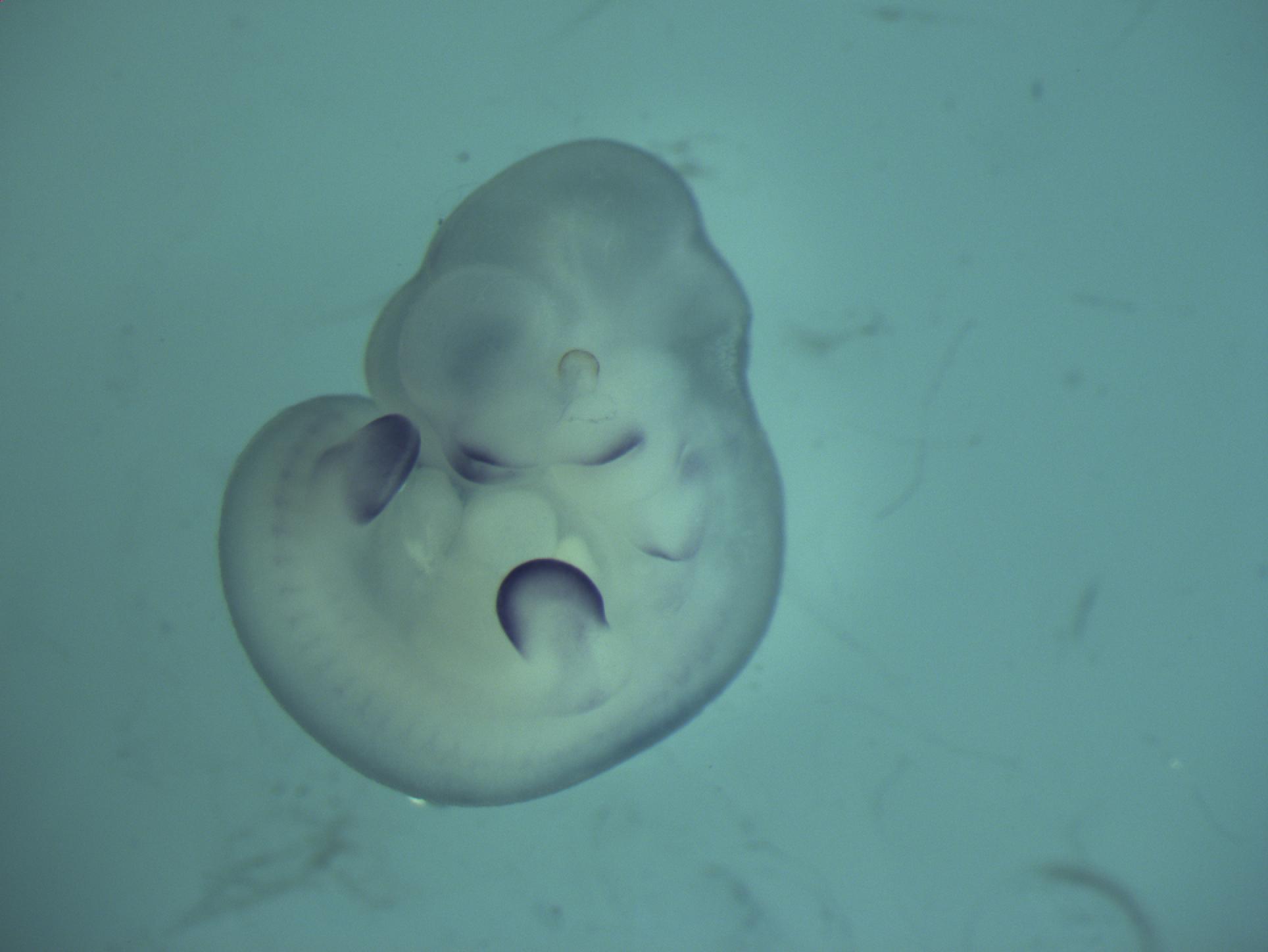

Supplement: Figure 2—source data 1. — This zip archive contains pictures, taken using a Leica MX16F microscope, of the right and left sides of the mouse embryos that underwent Dusp6 WMISH. Folders are organized by developmental stage and genotype. [file elife-36405-fig2-data1.zip › Figure 2 supplement 1-Source data 1/Dusp6 10.5 wt/Dusp6 10.5 wt3L.jpg]

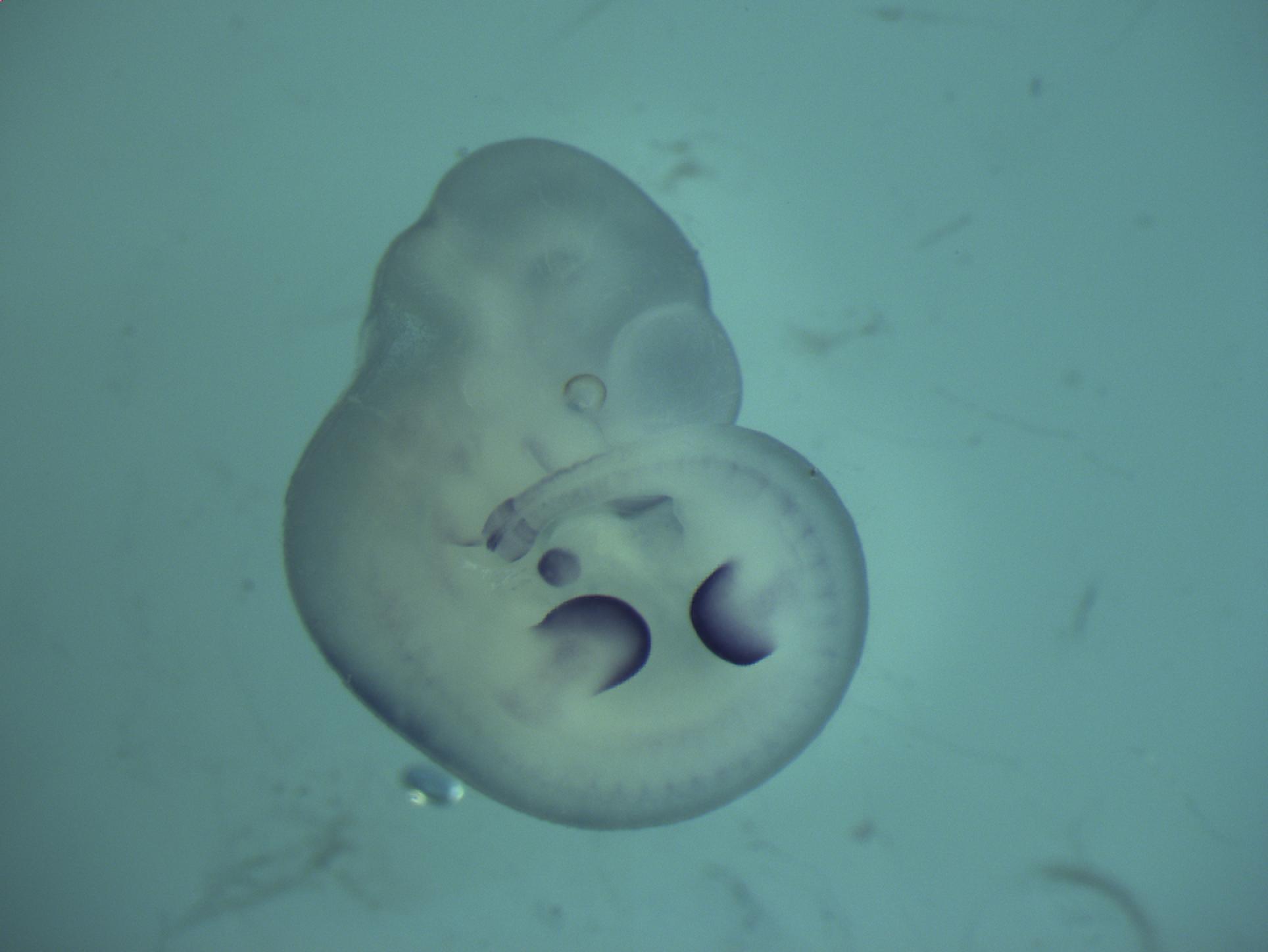

Supplement: Figure 2—source data 1. — This zip archive contains pictures, taken using a Leica MX16F microscope, of the right and left sides of the mouse embryos that underwent Dusp6 WMISH. Folders are organized by developmental stage and genotype. [file elife-36405-fig2-data1.zip › Figure 2 supplement 1-Source data 1/Dusp6 10.5 wt/Dusp6 10.5 wt3R.jpg]

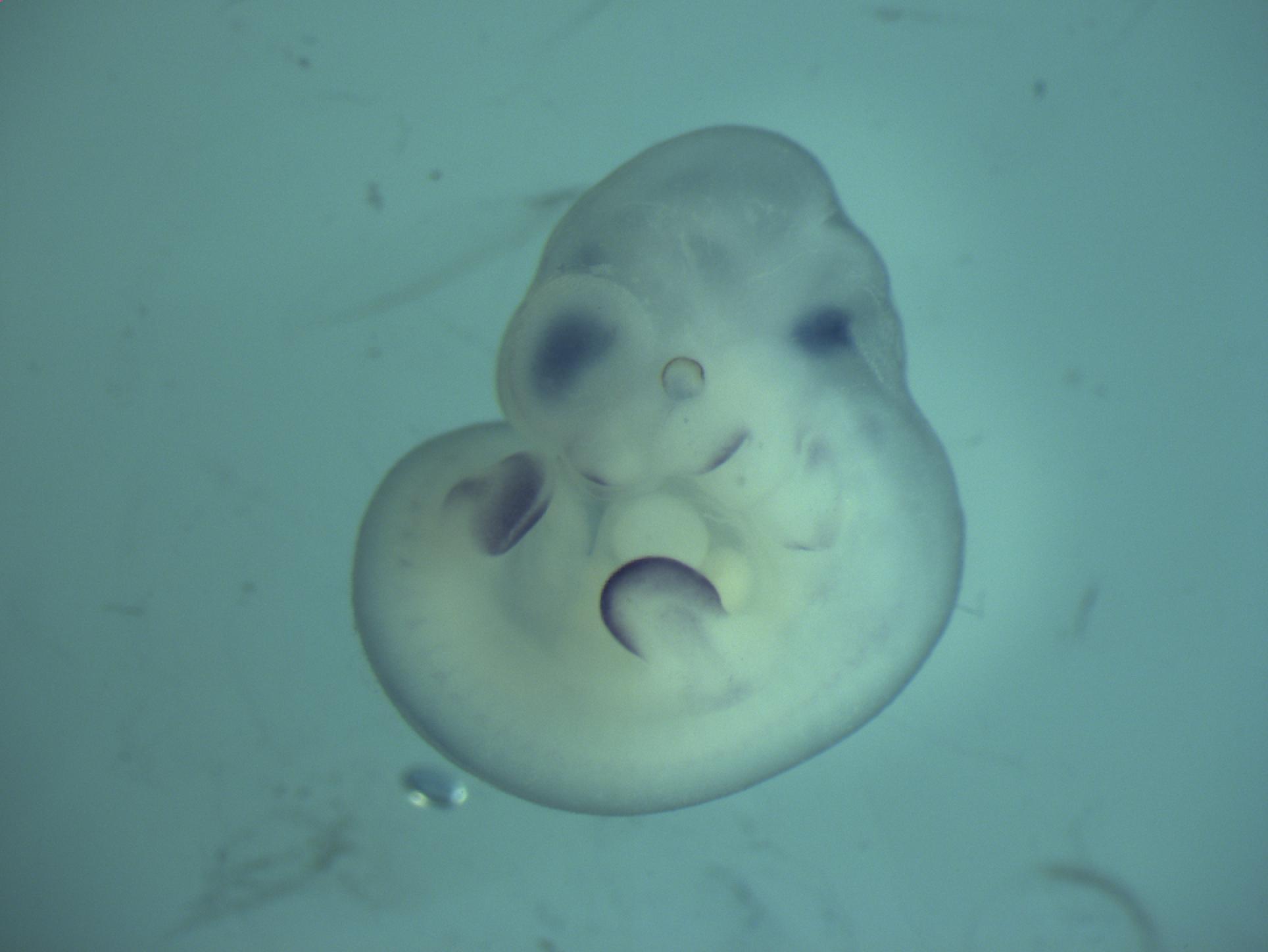

Supplement: Figure 2—source data 1. — This zip archive contains pictures, taken using a Leica MX16F microscope, of the right and left sides of the mouse embryos that underwent Dusp6 WMISH. Folders are organized by developmental stage and genotype. [file elife-36405-fig2-data1.zip › Figure 2 supplement 1-Source data 1/Dusp6 10.5 wt/Dusp6 10.5 wt4L.jpg]

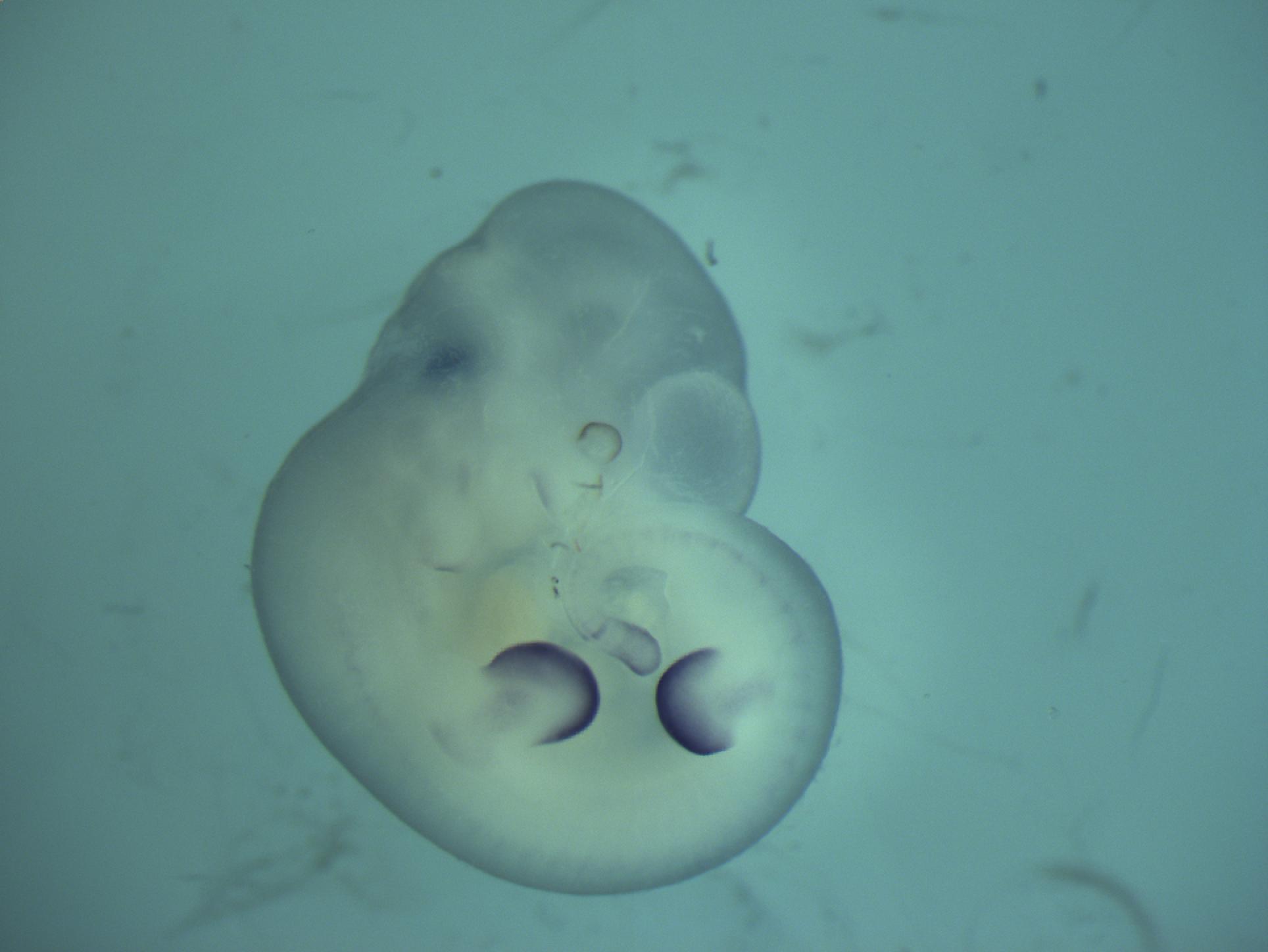

Supplement: Figure 2—source data 1. — This zip archive contains pictures, taken using a Leica MX16F microscope, of the right and left sides of the mouse embryos that underwent Dusp6 WMISH. Folders are organized by developmental stage and genotype. [file elife-36405-fig2-data1.zip › Figure 2 supplement 1-Source data 1/Dusp6 10.5 wt/Dusp6 10.5 wt4R.jpg]

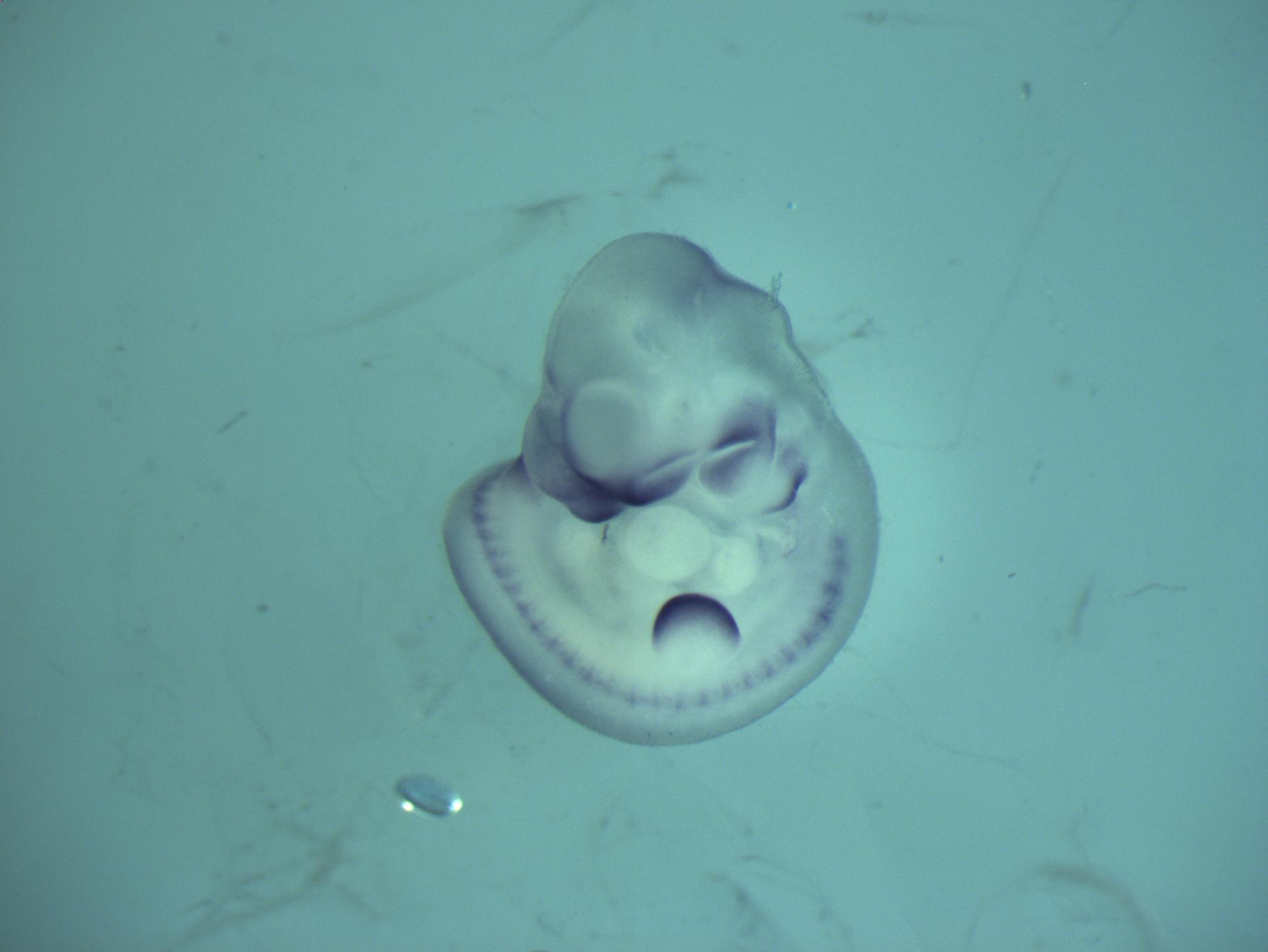

Supplement: Figure 2—source data 1. — This zip archive contains pictures, taken using a Leica MX16F microscope, of the right and left sides of the mouse embryos that underwent Dusp6 WMISH. Folders are organized by developmental stage and genotype. [file elife-36405-fig2-data1.zip › Figure 2 supplement 1-Source data 1/Dusp6 10.5 wt/Dusp6 10.5 wt5L.jpg]

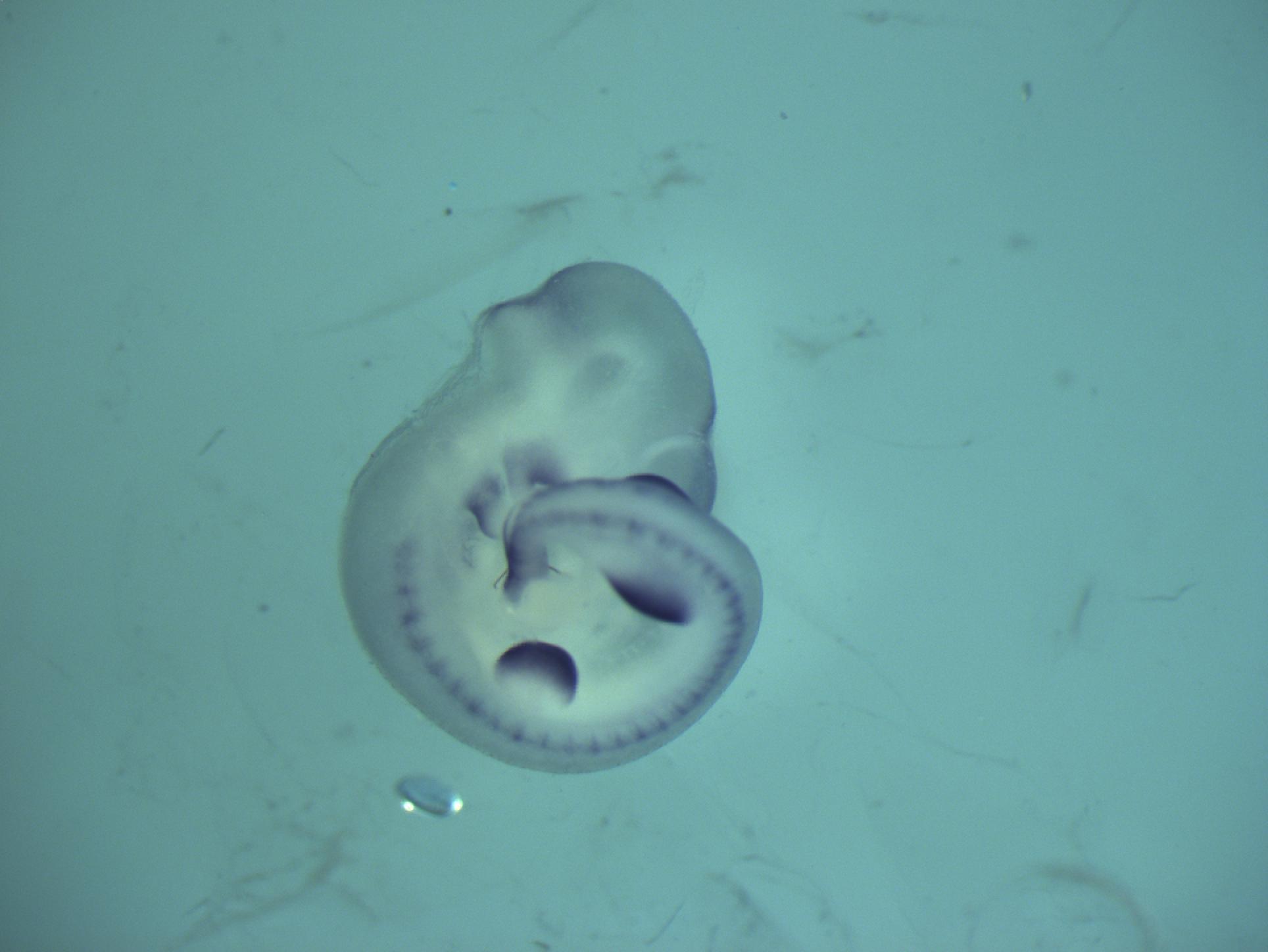

Supplement: Figure 2—source data 1. — This zip archive contains pictures, taken using a Leica MX16F microscope, of the right and left sides of the mouse embryos that underwent Dusp6 WMISH. Folders are organized by developmental stage and genotype. [file elife-36405-fig2-data1.zip › Figure 2 supplement 1-Source data 1/Dusp6 10.5 wt/Dusp6 10.5 wt5R.jpg]

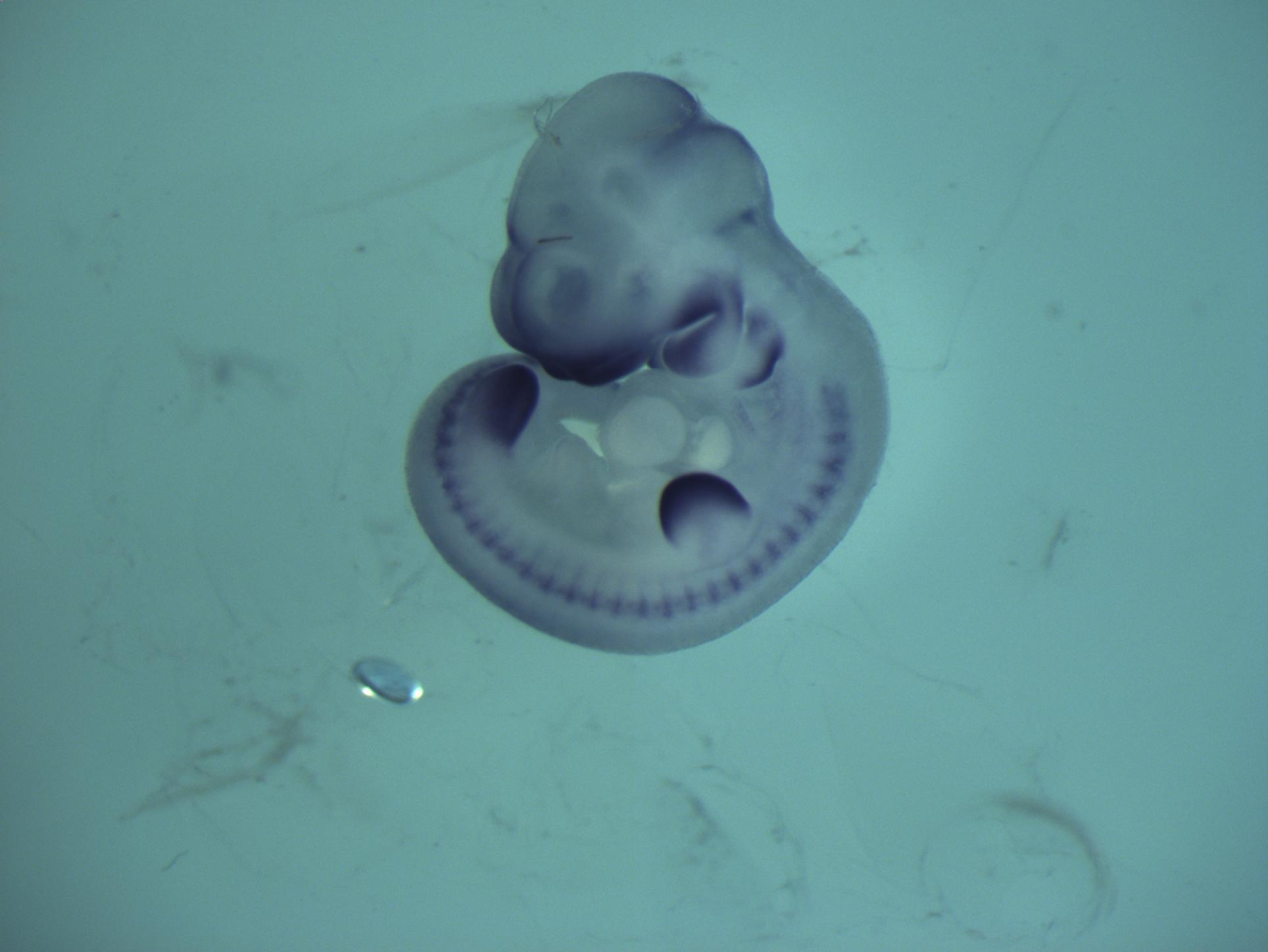

Supplement: Figure 2—source data 1. — This zip archive contains pictures, taken using a Leica MX16F microscope, of the right and left sides of the mouse embryos that underwent Dusp6 WMISH. Folders are organized by developmental stage and genotype. [file elife-36405-fig2-data1.zip › Figure 2 supplement 1-Source data 1/Dusp6 10.5 wt/Dusp6 10.5 wt6L.jpg]

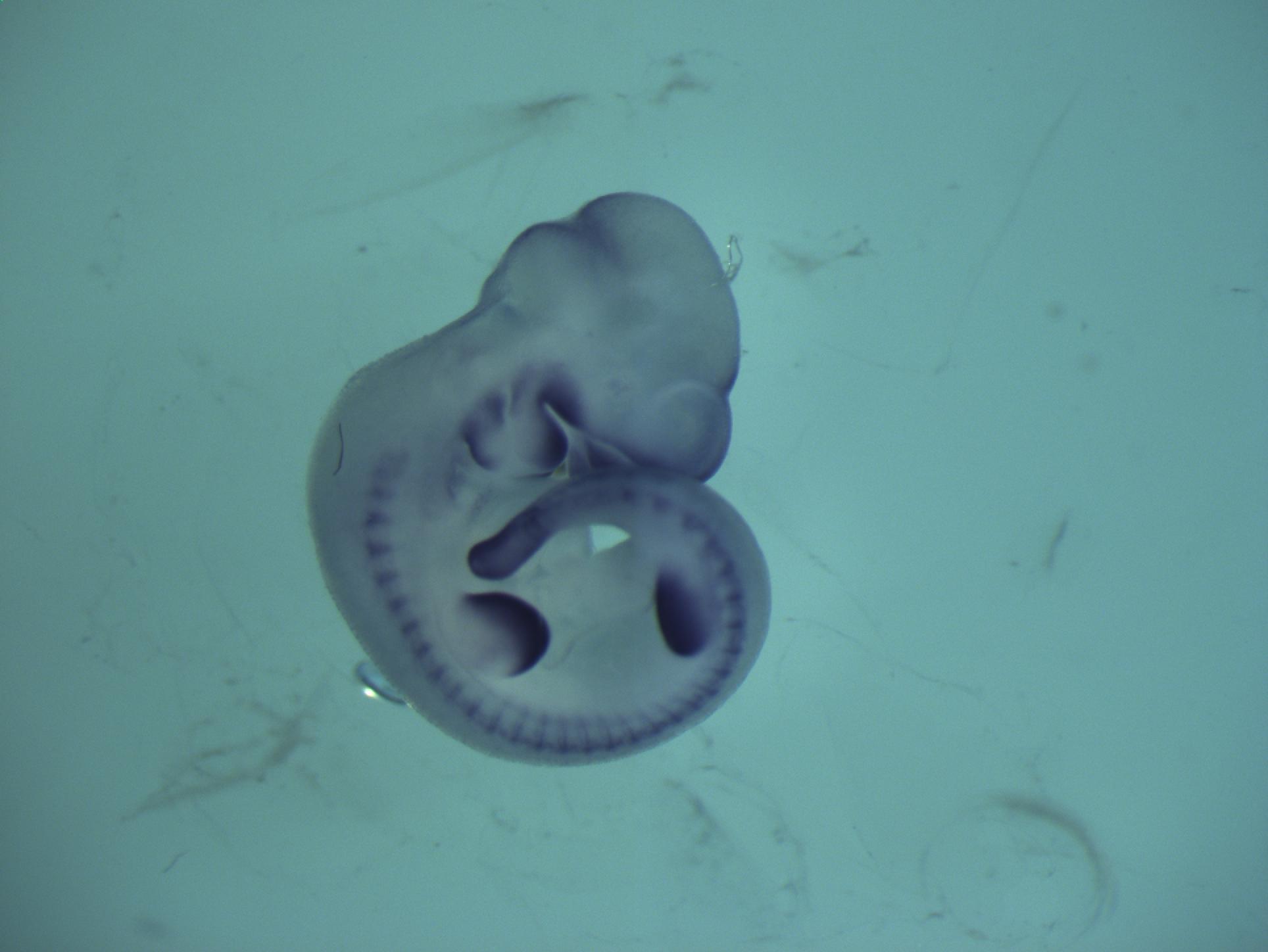

Supplement: Figure 2—source data 1. — This zip archive contains pictures, taken using a Leica MX16F microscope, of the right and left sides of the mouse embryos that underwent Dusp6 WMISH. Folders are organized by developmental stage and genotype. [file elife-36405-fig2-data1.zip › Figure 2 supplement 1-Source data 1/Dusp6 10.5 wt/Dusp6 10.5 wt6R.jpg]

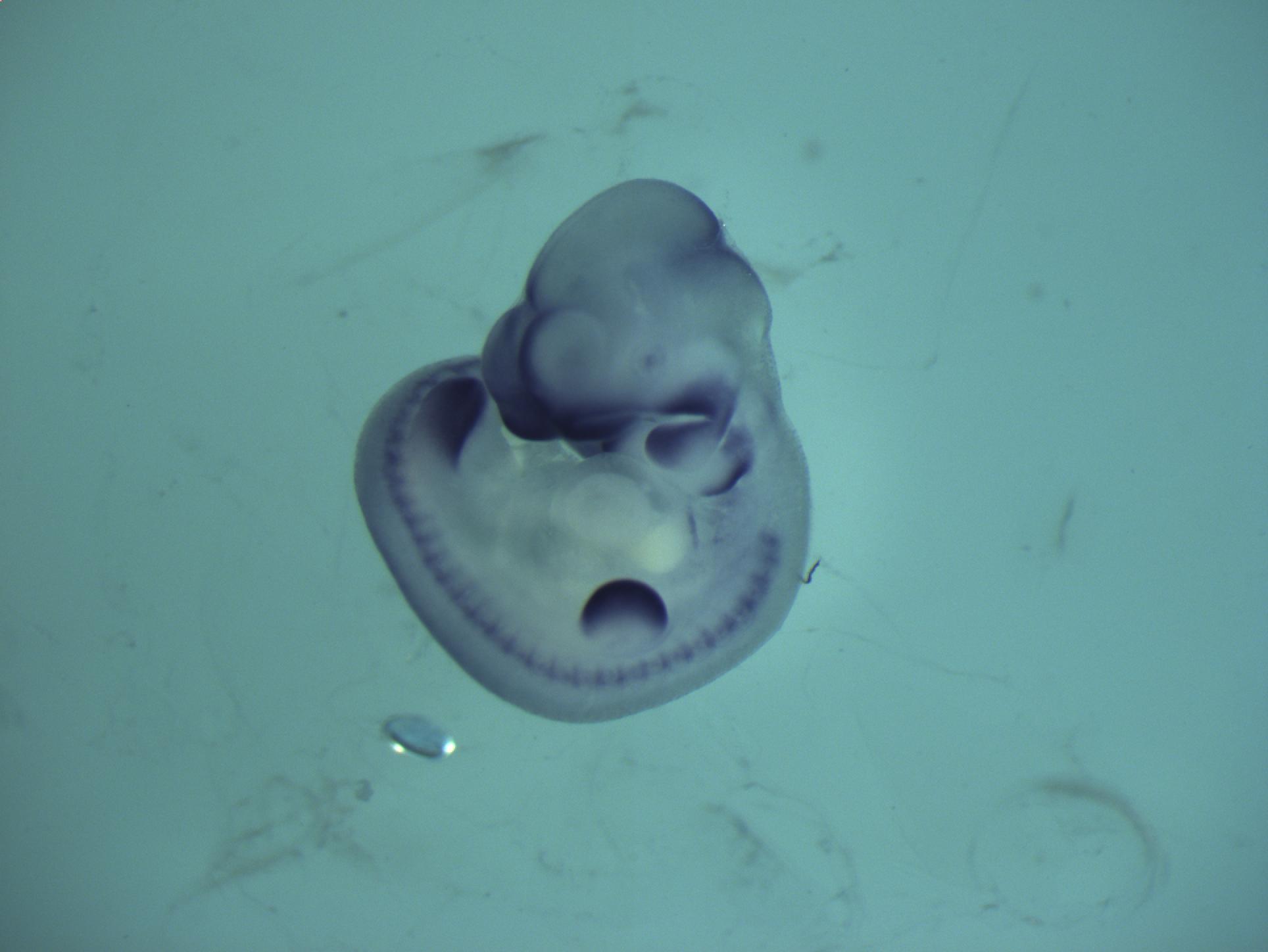

Supplement: Figure 2—source data 1. — This zip archive contains pictures, taken using a Leica MX16F microscope, of the right and left sides of the mouse embryos that underwent Dusp6 WMISH. Folders are organized by developmental stage and genotype. [file elife-36405-fig2-data1.zip › Figure 2 supplement 1-Source data 1/Dusp6 10.5 wt/Dusp6 10.5 wt7L.jpg]

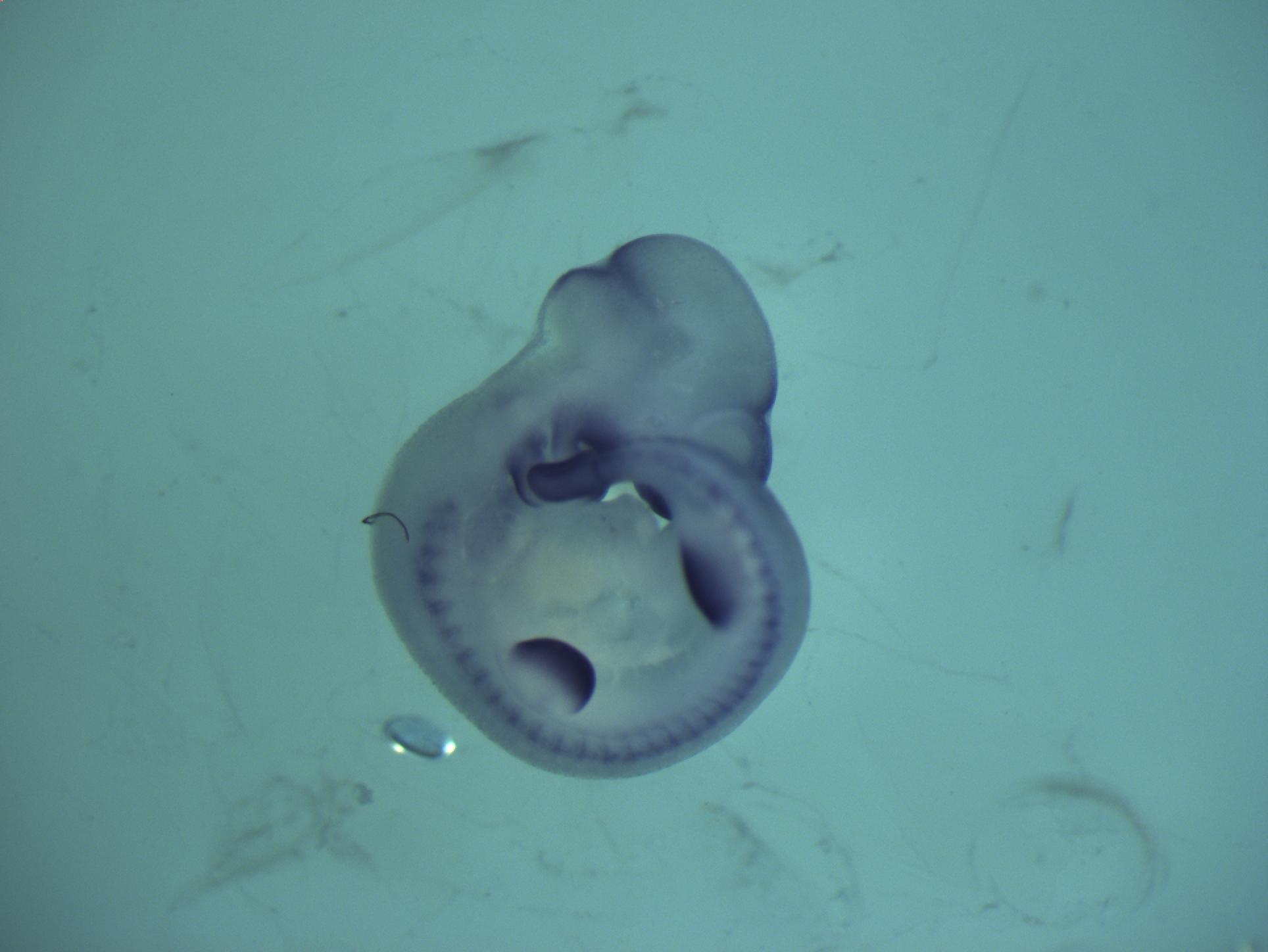

Supplement: Figure 2—source data 1. — This zip archive contains pictures, taken using a Leica MX16F microscope, of the right and left sides of the mouse embryos that underwent Dusp6 WMISH. Folders are organized by developmental stage and genotype. [file elife-36405-fig2-data1.zip › Figure 2 supplement 1-Source data 1/Dusp6 10.5 wt/Dusp6 10.5 wt7R.jpg]

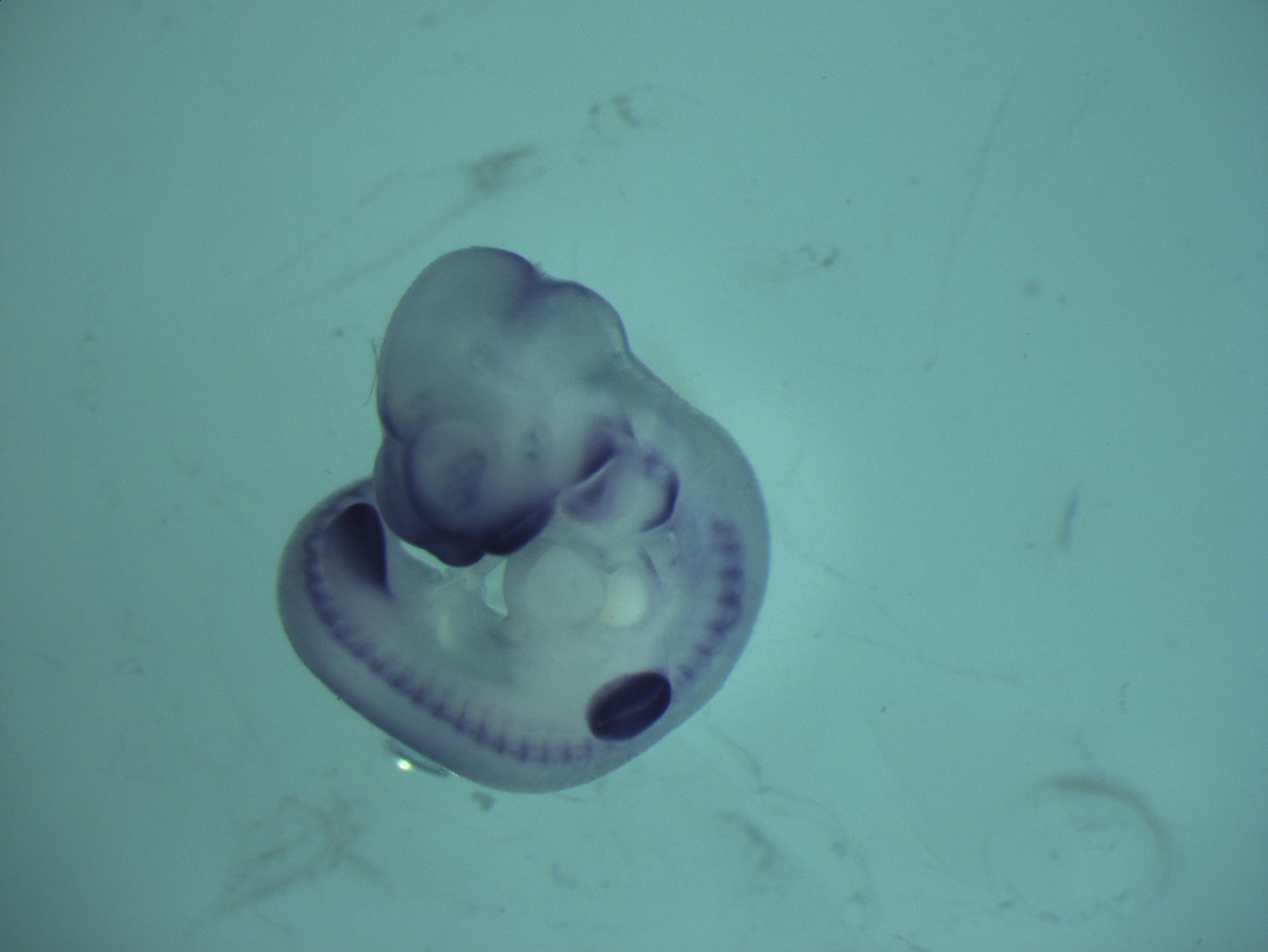

Supplement: Figure 2—source data 1. — This zip archive contains pictures, taken using a Leica MX16F microscope, of the right and left sides of the mouse embryos that underwent Dusp6 WMISH. Folders are organized by developmental stage and genotype. [file elife-36405-fig2-data1.zip › Figure 2 supplement 1-Source data 1/Dusp6 10.5 wt/Dusp6 10.5 wt8L.jpg]

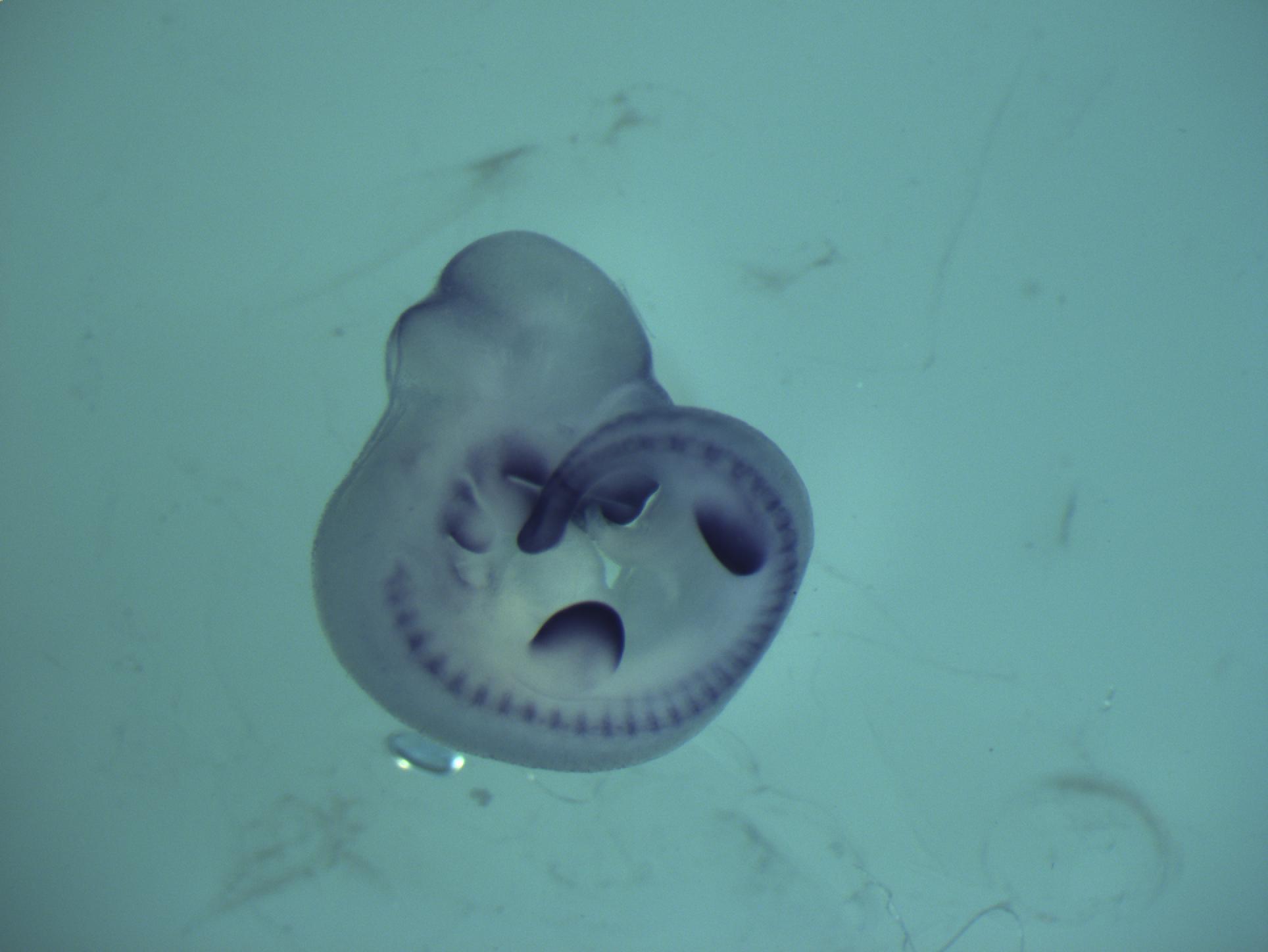

Supplement: Figure 2—source data 1. — This zip archive contains pictures, taken using a Leica MX16F microscope, of the right and left sides of the mouse embryos that underwent Dusp6 WMISH. Folders are organized by developmental stage and genotype. [file elife-36405-fig2-data1.zip › Figure 2 supplement 1-Source data 1/Dusp6 10.5 wt/Dusp6 10.5 wt8R.jpg]

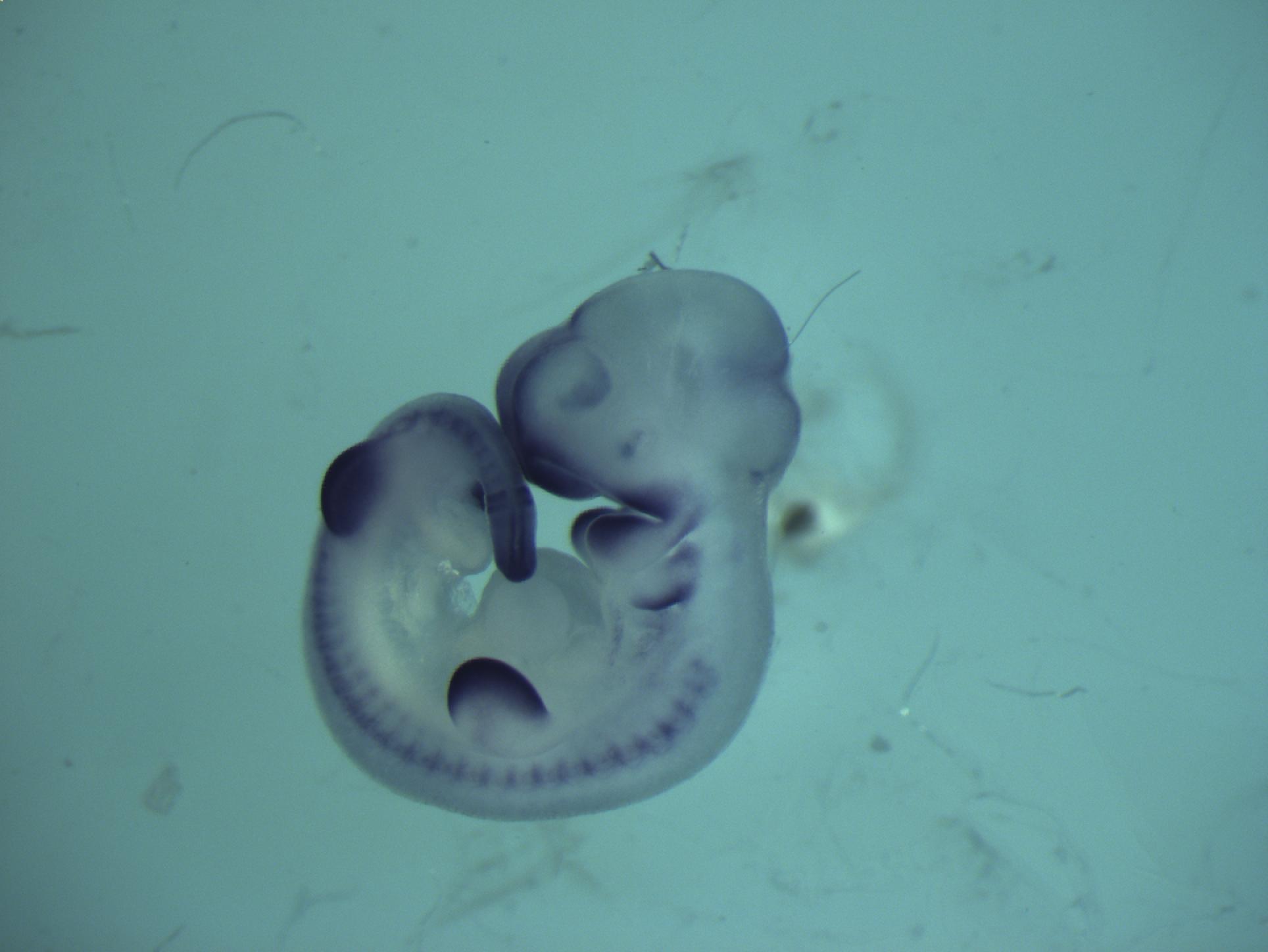

Supplement: Figure 2—source data 1. — This zip archive contains pictures, taken using a Leica MX16F microscope, of the right and left sides of the mouse embryos that underwent Dusp6 WMISH. Folders are organized by developmental stage and genotype. [file elife-36405-fig2-data1.zip › Figure 2 supplement 1-Source data 1/Dusp6 10.5 wt/Dusp6 10.5 wt9L.jpg]

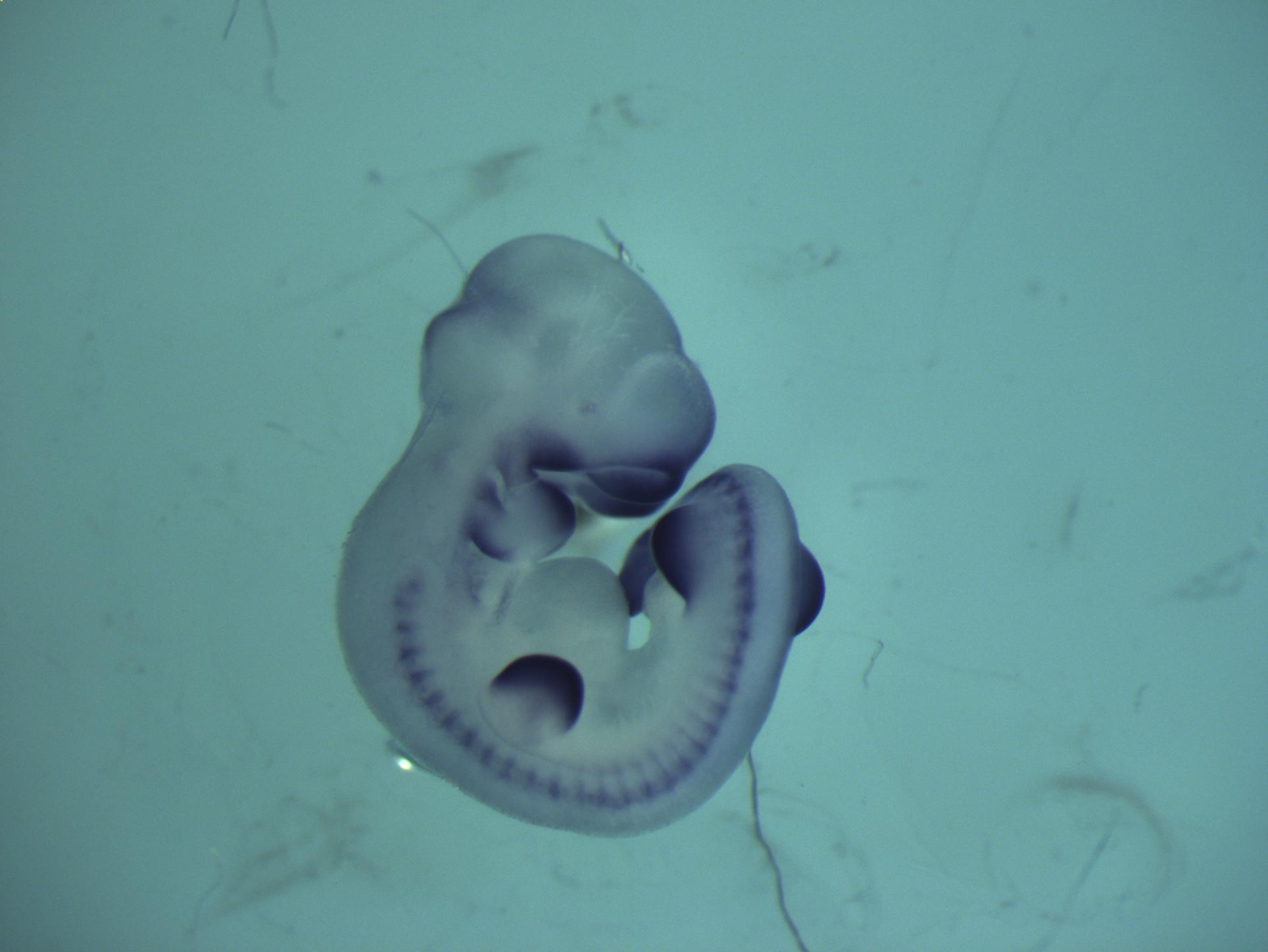

Supplement: Figure 2—source data 1. — This zip archive contains pictures, taken using a Leica MX16F microscope, of the right and left sides of the mouse embryos that underwent Dusp6 WMISH. Folders are organized by developmental stage and genotype. [file elife-36405-fig2-data1.zip › Figure 2 supplement 1-Source data 1/Dusp6 10.5 wt/Dusp6 10.5 wt9R.jpg]

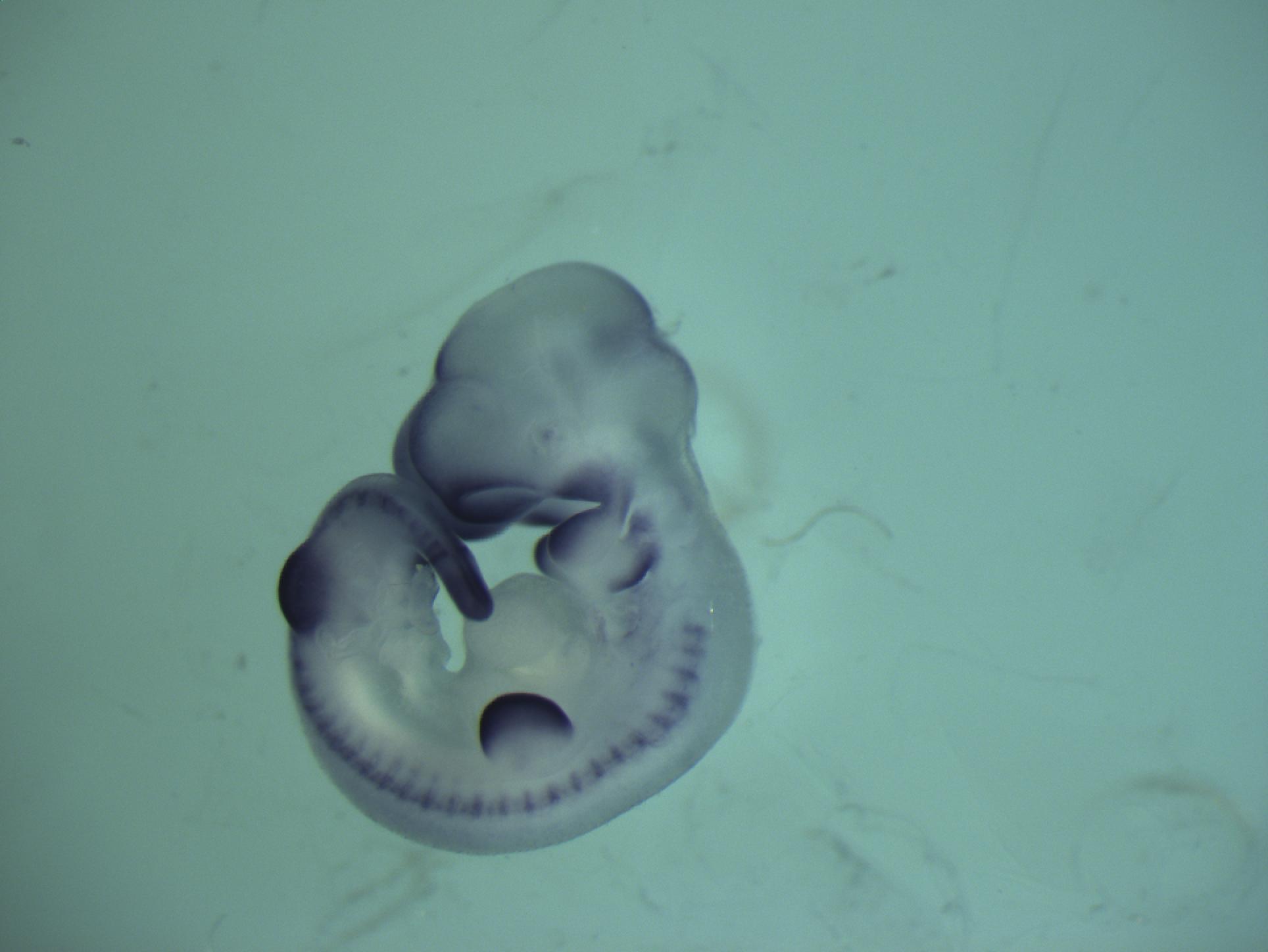

Supplement: Figure 2—source data 1. — This zip archive contains pictures, taken using a Leica MX16F microscope, of the right and left sides of the mouse embryos that underwent Dusp6 WMISH. Folders are organized by developmental stage and genotype. [file elife-36405-fig2-data1.zip › Figure 2 supplement 1-Source data 1/Dusp6 11.5 mut/Dusp6 11.5 mut1L.jpg]

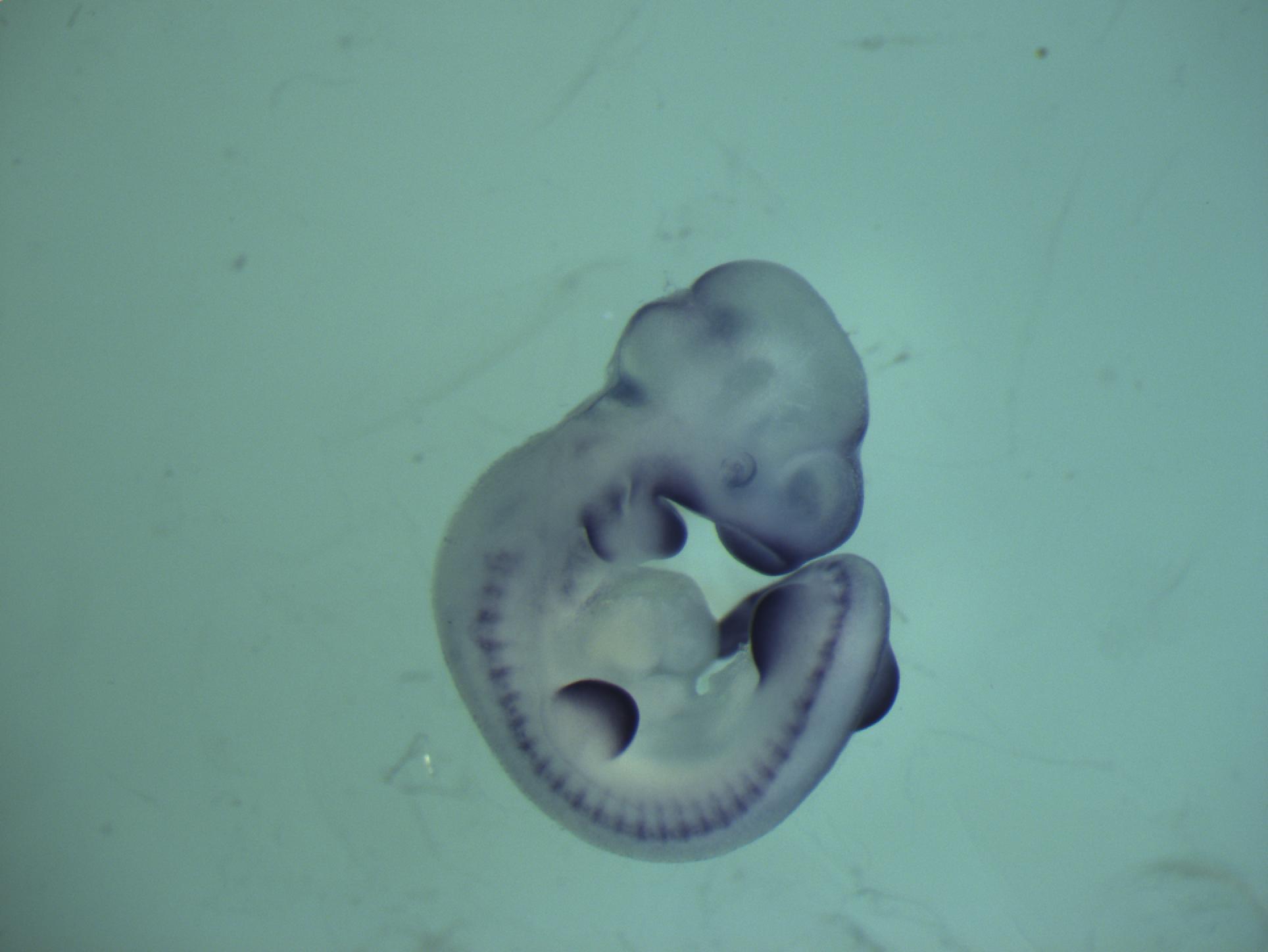

Supplement: Figure 2—source data 1. — This zip archive contains pictures, taken using a Leica MX16F microscope, of the right and left sides of the mouse embryos that underwent Dusp6 WMISH. Folders are organized by developmental stage and genotype. [file elife-36405-fig2-data1.zip › Figure 2 supplement 1-Source data 1/Dusp6 11.5 mut/Dusp6 11.5 mut1R.jpg]

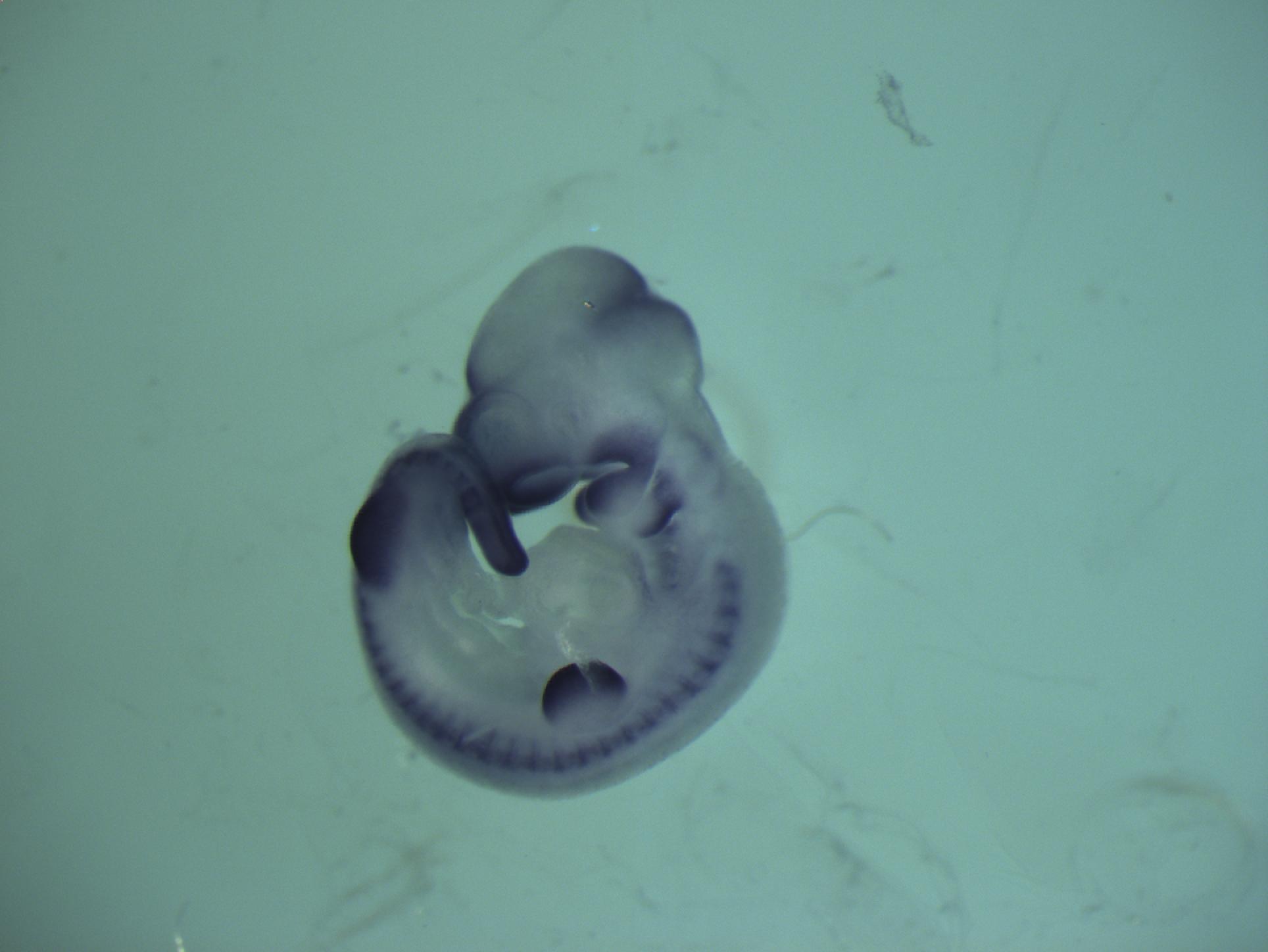

Supplement: Figure 2—source data 1. — This zip archive contains pictures, taken using a Leica MX16F microscope, of the right and left sides of the mouse embryos that underwent Dusp6 WMISH. Folders are organized by developmental stage and genotype. [file elife-36405-fig2-data1.zip › Figure 2 supplement 1-Source data 1/Dusp6 11.5 mut/Dusp6 11.5 mut2L.jpg]

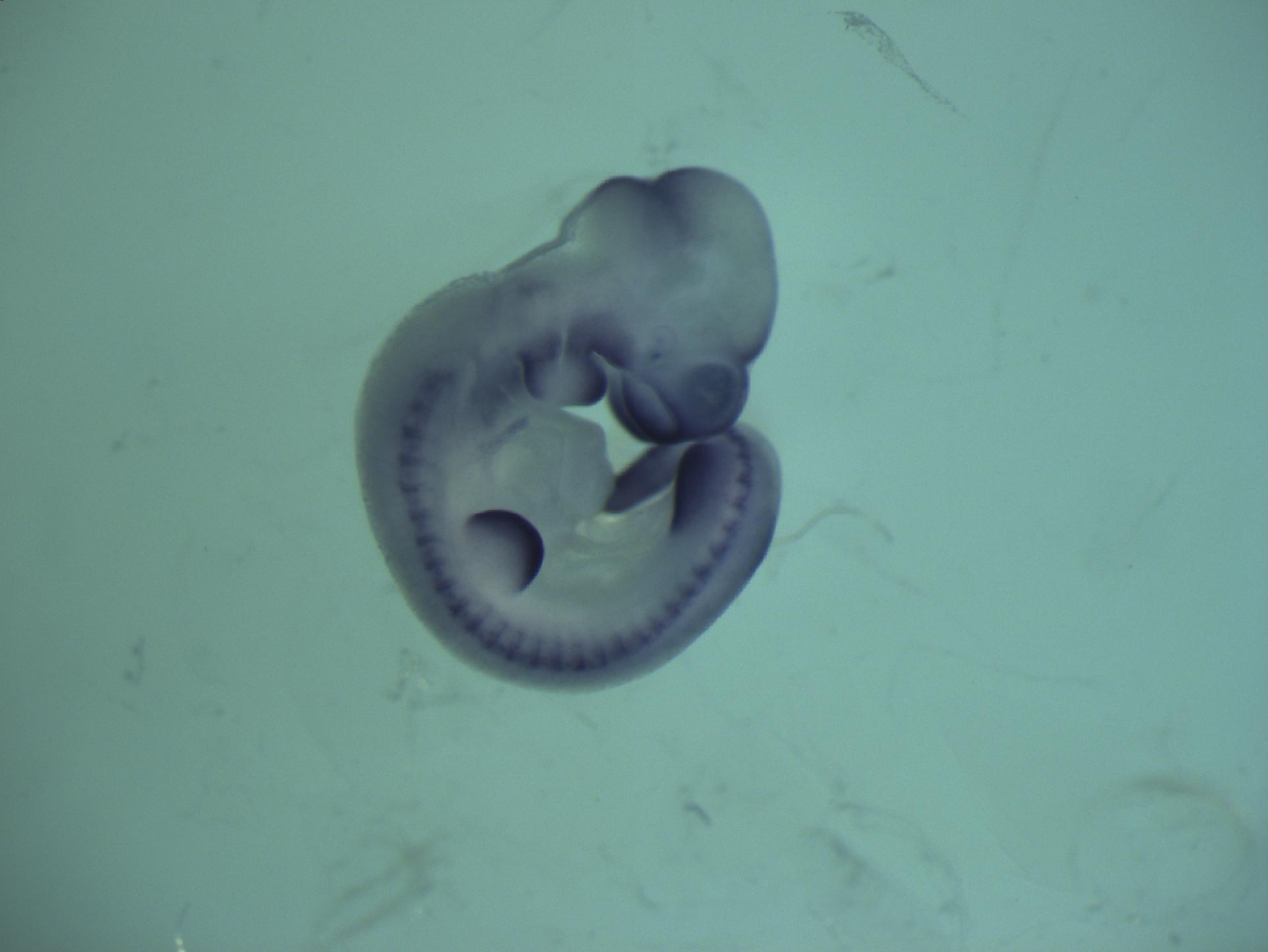

Supplement: Figure 2—source data 1. — This zip archive contains pictures, taken using a Leica MX16F microscope, of the right and left sides of the mouse embryos that underwent Dusp6 WMISH. Folders are organized by developmental stage and genotype. [file elife-36405-fig2-data1.zip › Figure 2 supplement 1-Source data 1/Dusp6 11.5 mut/Dusp6 11.5 mut2R.jpg]

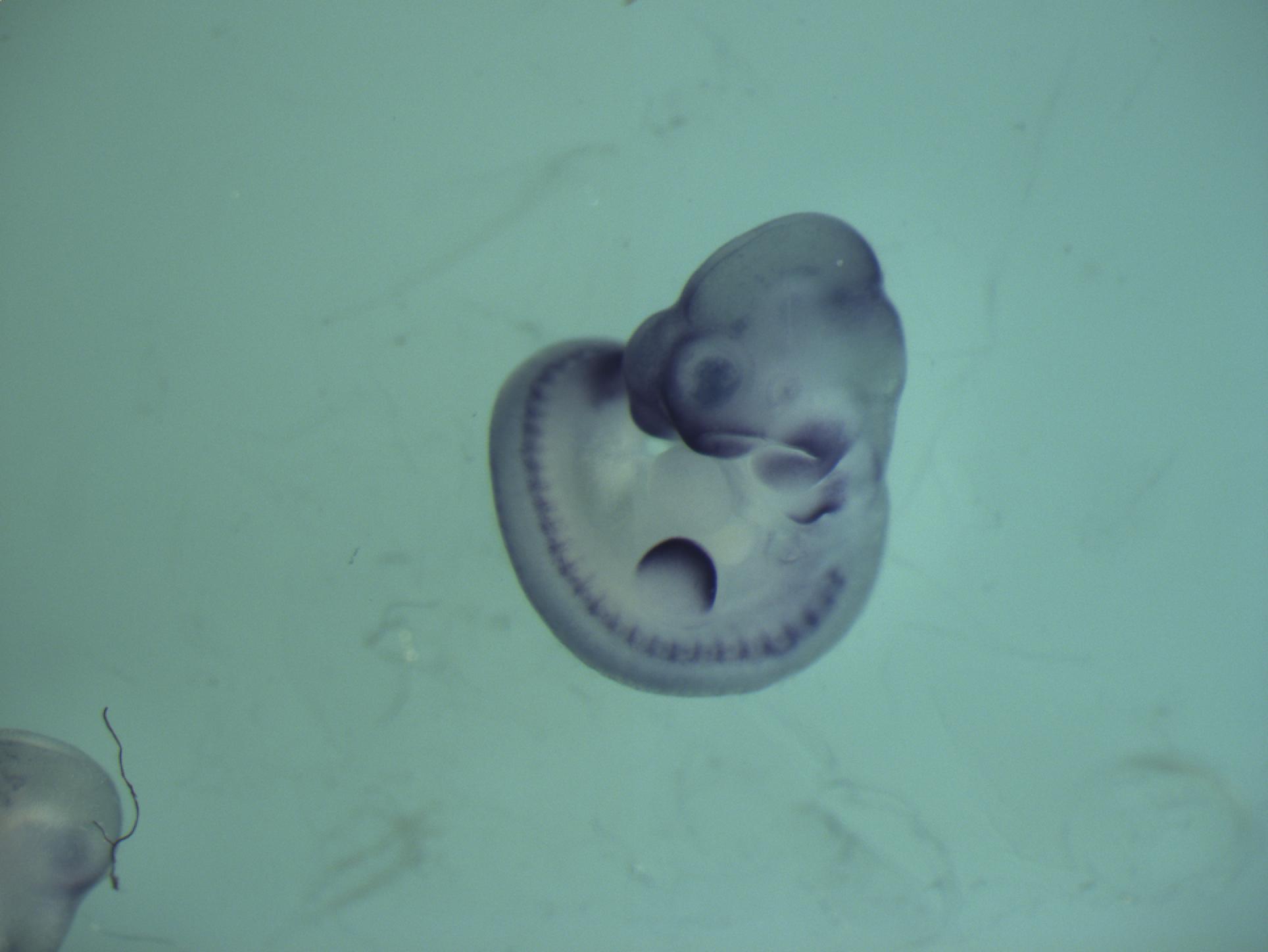

Supplement: Figure 2—source data 1. — This zip archive contains pictures, taken using a Leica MX16F microscope, of the right and left sides of the mouse embryos that underwent Dusp6 WMISH. Folders are organized by developmental stage and genotype. [file elife-36405-fig2-data1.zip › Figure 2 supplement 1-Source data 1/Dusp6 11.5 mut/Dusp6 11.5 mut3L.jpg]

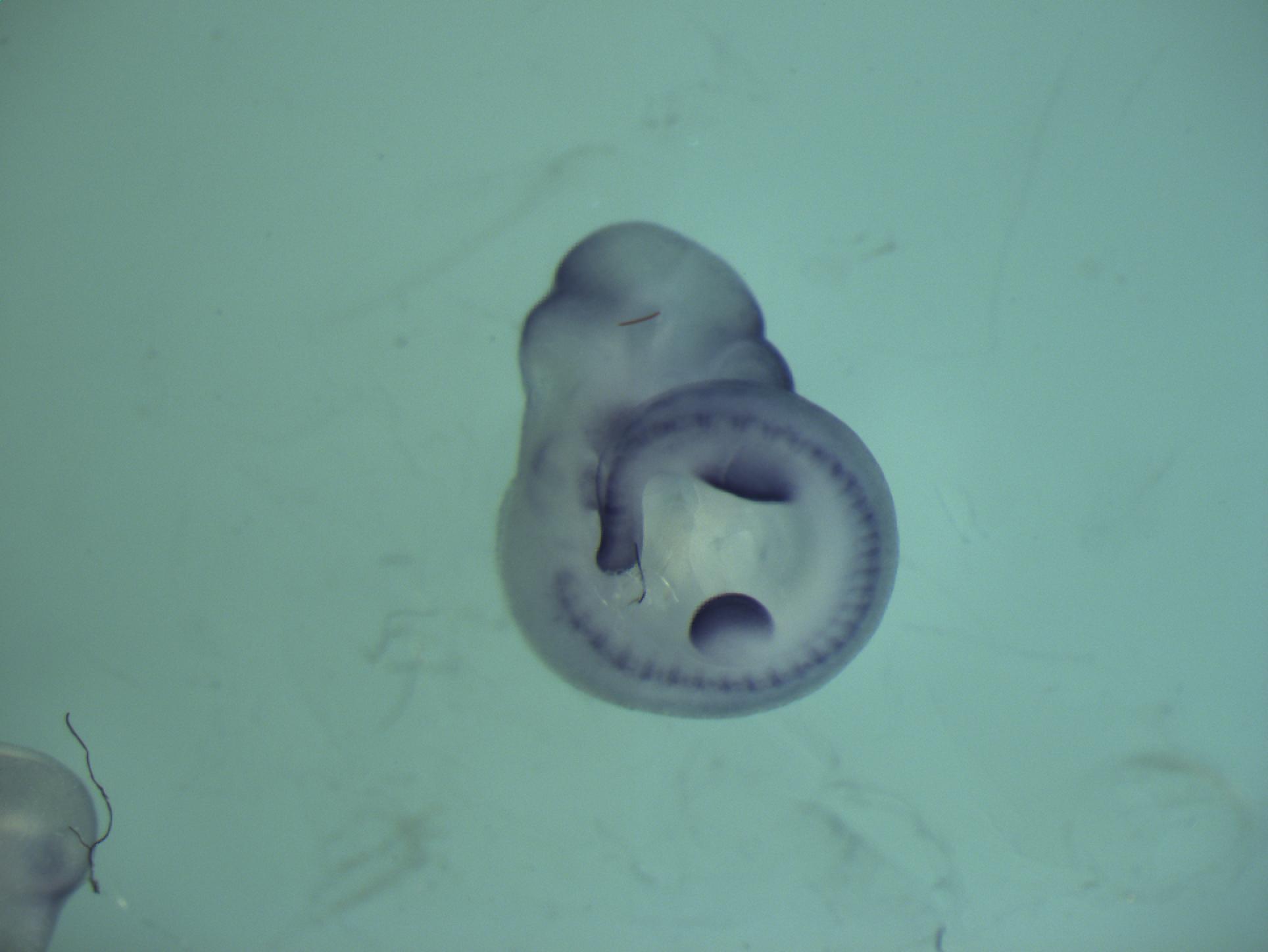

Supplement: Figure 2—source data 1. — This zip archive contains pictures, taken using a Leica MX16F microscope, of the right and left sides of the mouse embryos that underwent Dusp6 WMISH. Folders are organized by developmental stage and genotype. [file elife-36405-fig2-data1.zip › Figure 2 supplement 1-Source data 1/Dusp6 11.5 mut/Dusp6 11.5 mut3R.jpg]

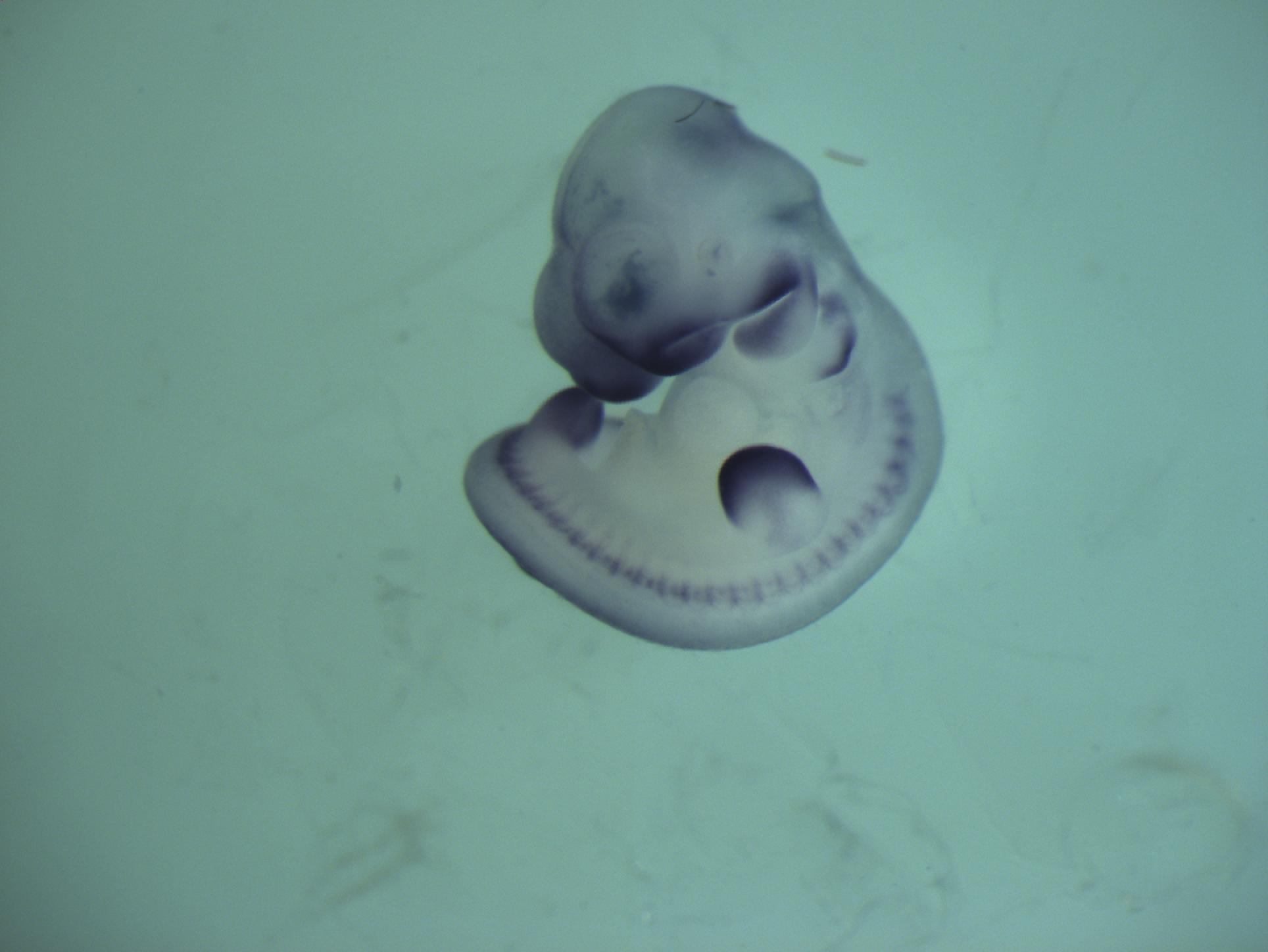

Supplement: Figure 2—source data 1. — This zip archive contains pictures, taken using a Leica MX16F microscope, of the right and left sides of the mouse embryos that underwent Dusp6 WMISH. Folders are organized by developmental stage and genotype. [file elife-36405-fig2-data1.zip › Figure 2 supplement 1-Source data 1/Dusp6 11.5 mut/Dusp6 11.5 mut4L.jpg]

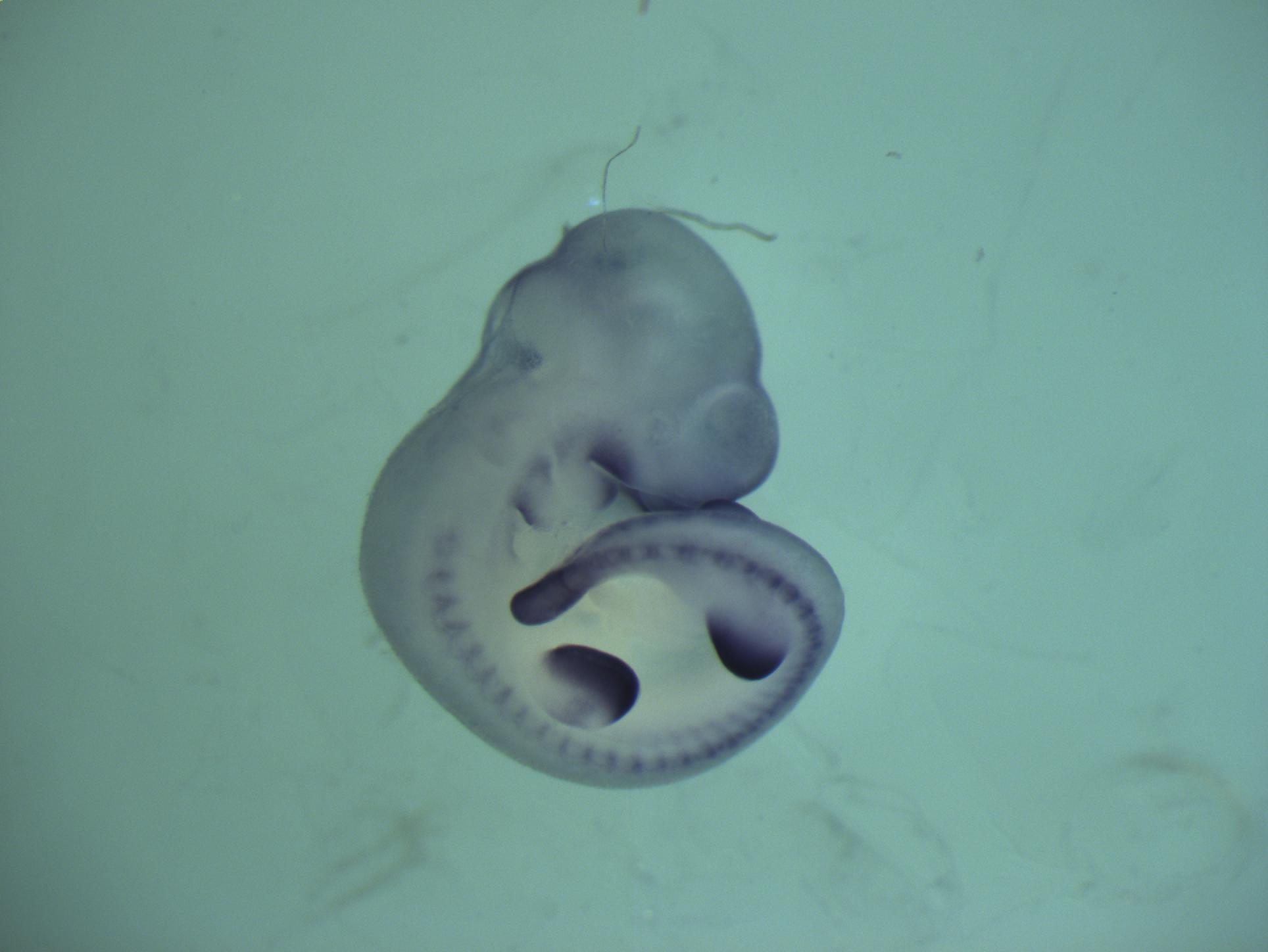

Supplement: Figure 2—source data 1. — This zip archive contains pictures, taken using a Leica MX16F microscope, of the right and left sides of the mouse embryos that underwent Dusp6 WMISH. Folders are organized by developmental stage and genotype. [file elife-36405-fig2-data1.zip › Figure 2 supplement 1-Source data 1/Dusp6 11.5 mut/Dusp6 11.5 mut4R.jpg]

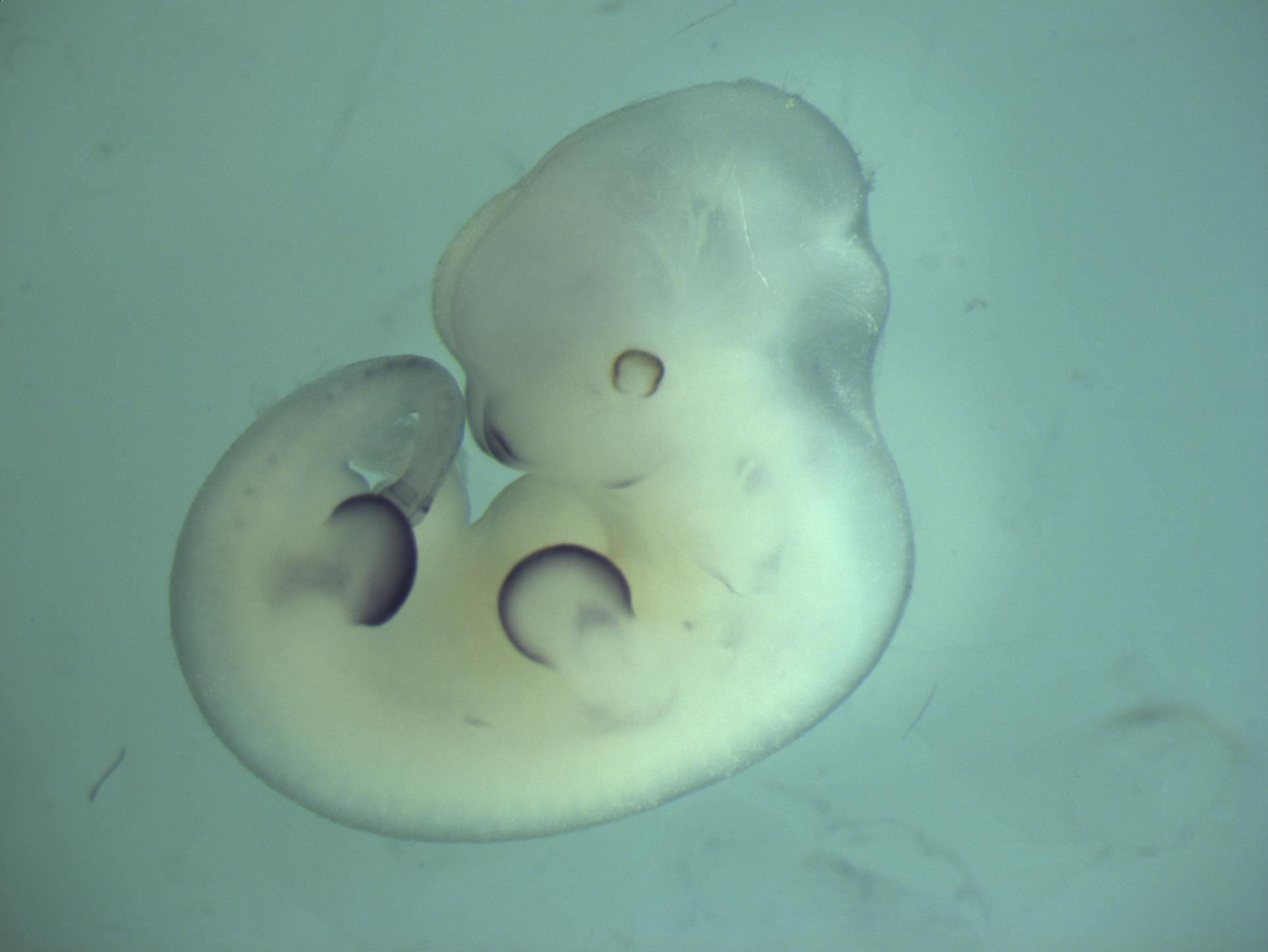

Supplement: Figure 2—source data 1. — This zip archive contains pictures, taken using a Leica MX16F microscope, of the right and left sides of the mouse embryos that underwent Dusp6 WMISH. Folders are organized by developmental stage and genotype. [file elife-36405-fig2-data1.zip › Figure 2 supplement 1-Source data 1/Dusp6 11.5 mut/Dusp6 11.5 mut5L.jpg]

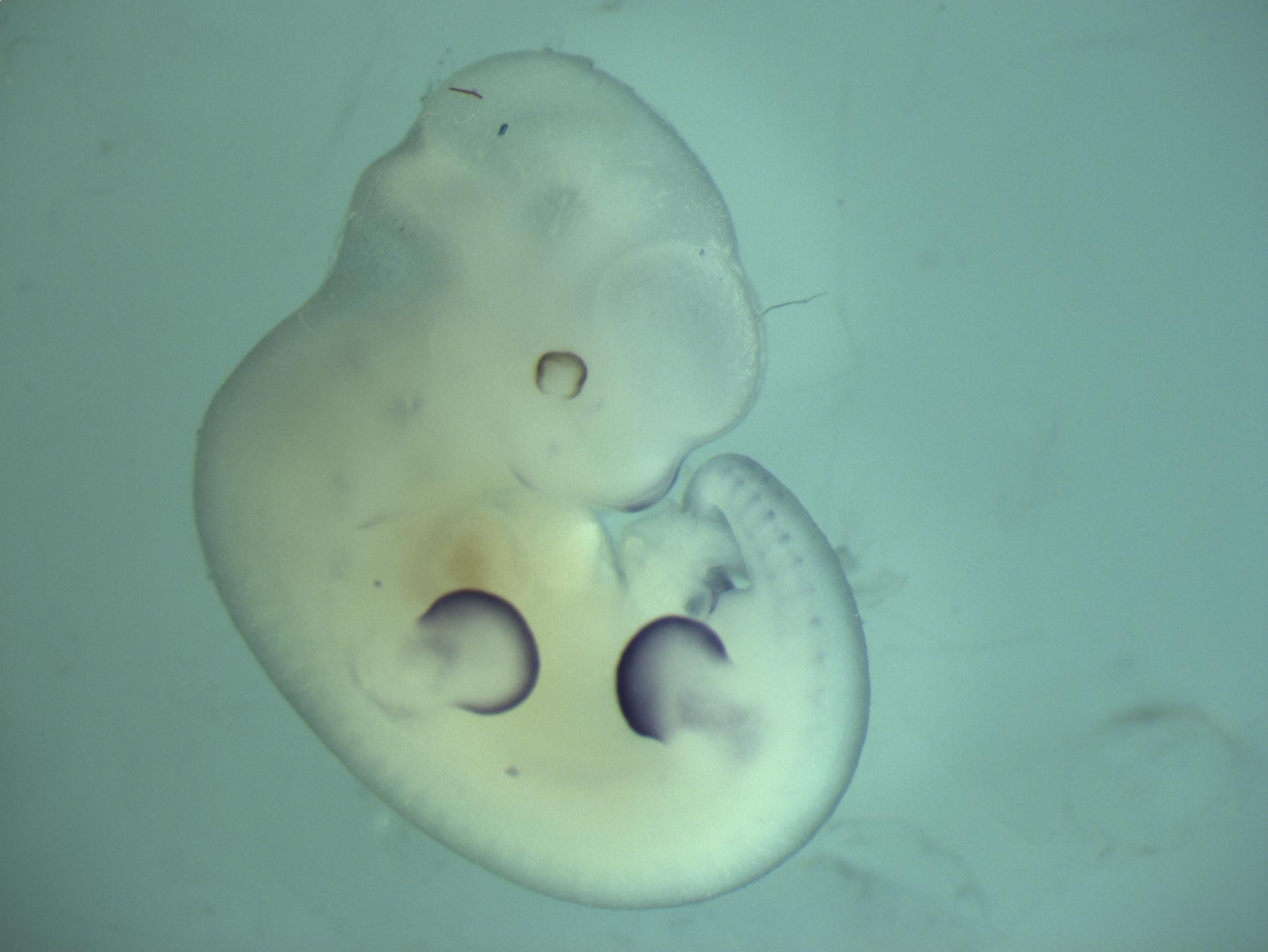

Supplement: Figure 2—source data 1. — This zip archive contains pictures, taken using a Leica MX16F microscope, of the right and left sides of the mouse embryos that underwent Dusp6 WMISH. Folders are organized by developmental stage and genotype. [file elife-36405-fig2-data1.zip › Figure 2 supplement 1-Source data 1/Dusp6 11.5 mut/Dusp6 11.5 mut5R.jpg]

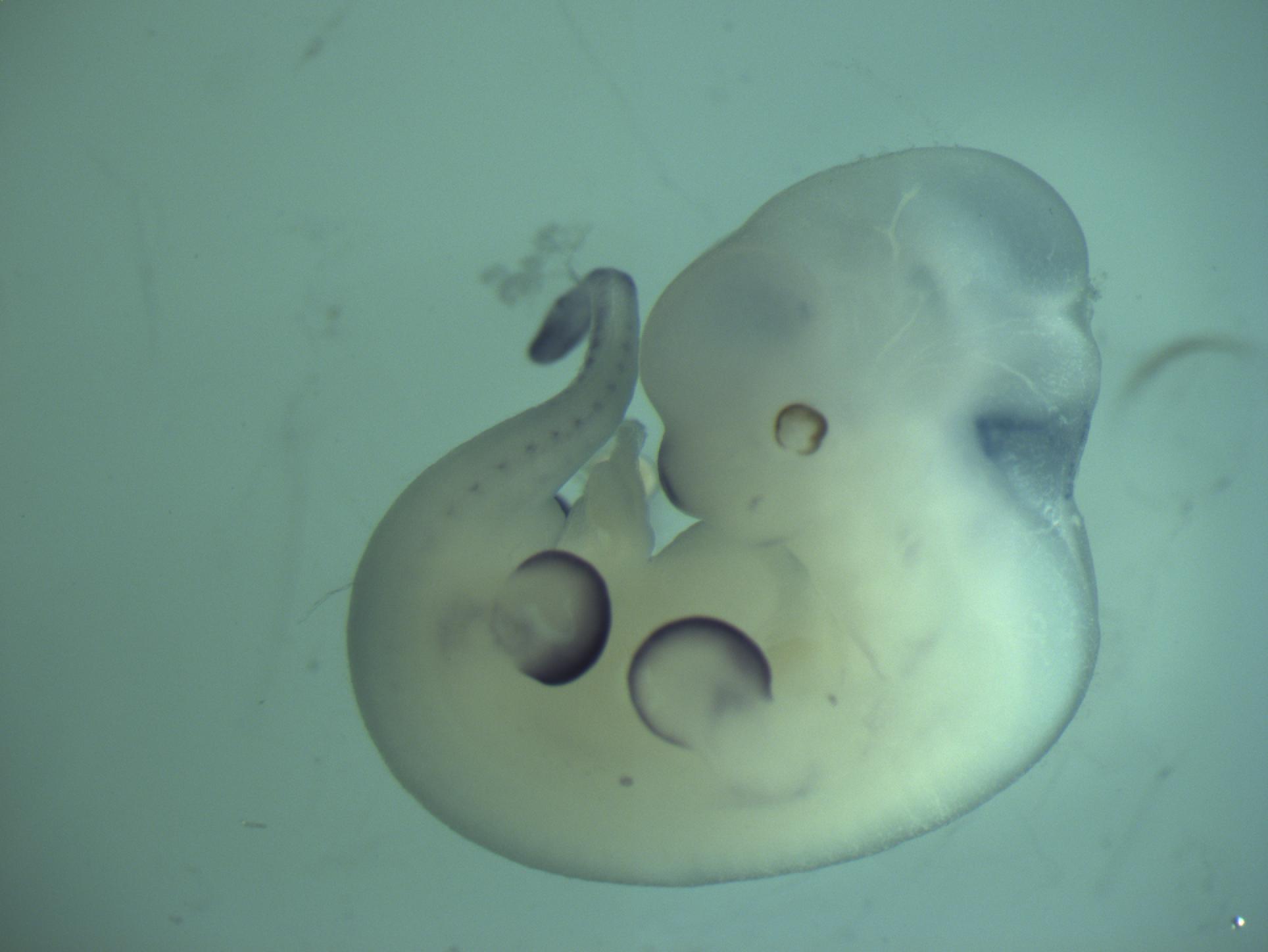

Supplement: Figure 2—source data 1. — This zip archive contains pictures, taken using a Leica MX16F microscope, of the right and left sides of the mouse embryos that underwent Dusp6 WMISH. Folders are organized by developmental stage and genotype. [file elife-36405-fig2-data1.zip › Figure 2 supplement 1-Source data 1/Dusp6 11.5 mut/Dusp6 11.5 mut6L.jpg]

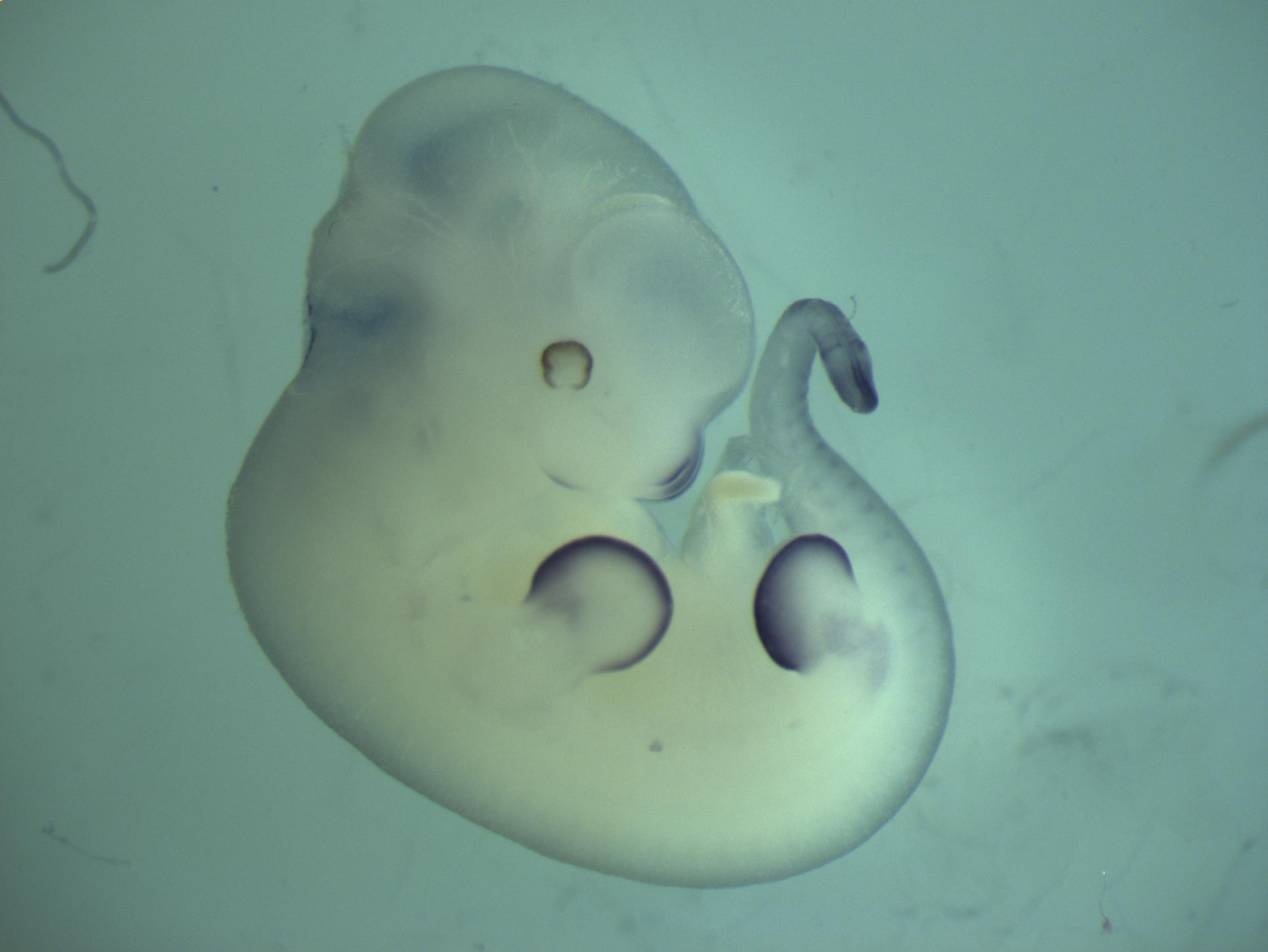

Supplement: Figure 2—source data 1. — This zip archive contains pictures, taken using a Leica MX16F microscope, of the right and left sides of the mouse embryos that underwent Dusp6 WMISH. Folders are organized by developmental stage and genotype. [file elife-36405-fig2-data1.zip › Figure 2 supplement 1-Source data 1/Dusp6 11.5 mut/Dusp6 11.5 mut6R.jpg]

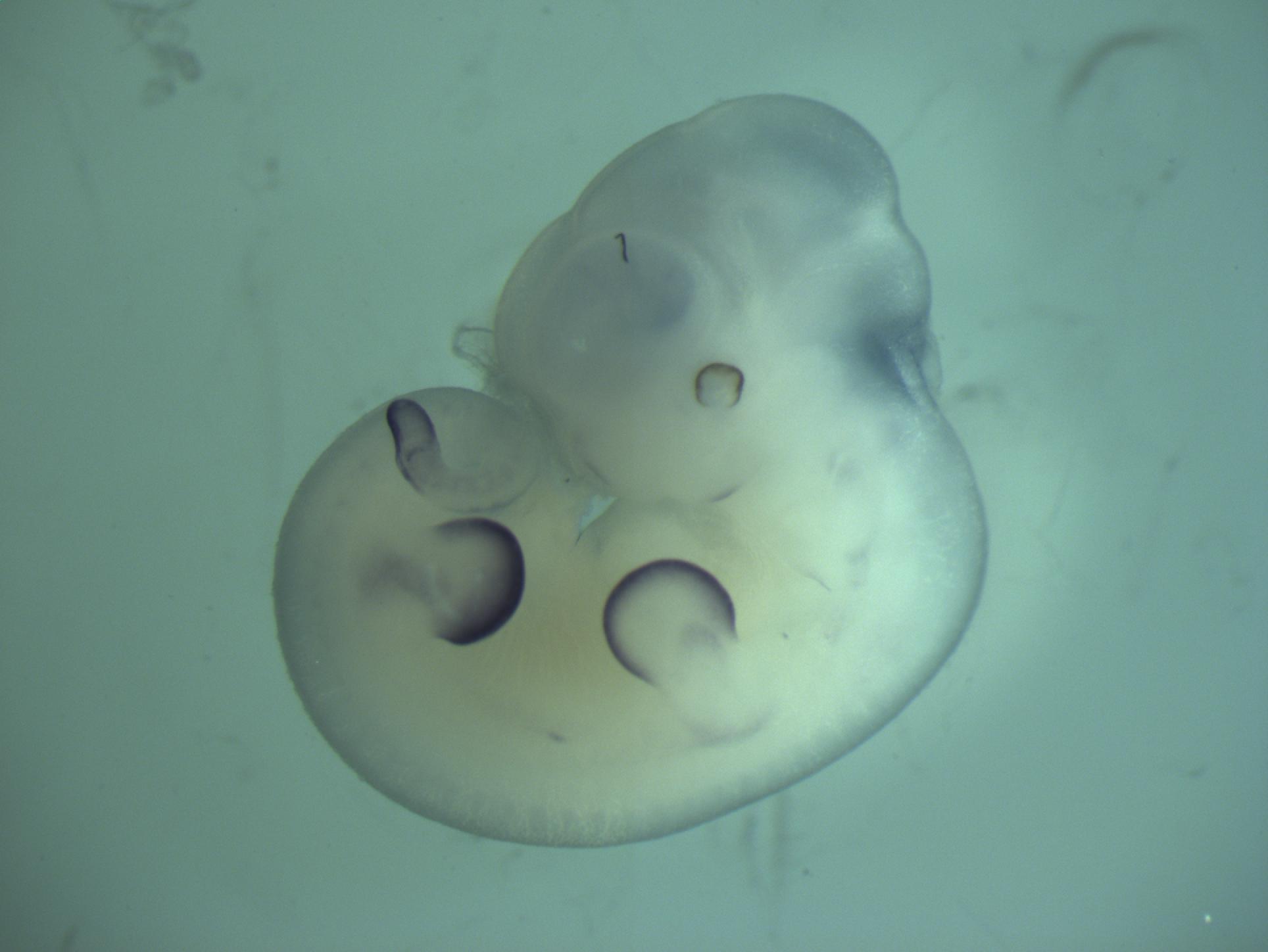

Supplement: Figure 2—source data 1. — This zip archive contains pictures, taken using a Leica MX16F microscope, of the right and left sides of the mouse embryos that underwent Dusp6 WMISH. Folders are organized by developmental stage and genotype. [file elife-36405-fig2-data1.zip › Figure 2 supplement 1-Source data 1/Dusp6 11.5 mut/Dusp6 11.5 mut7L.jpg]

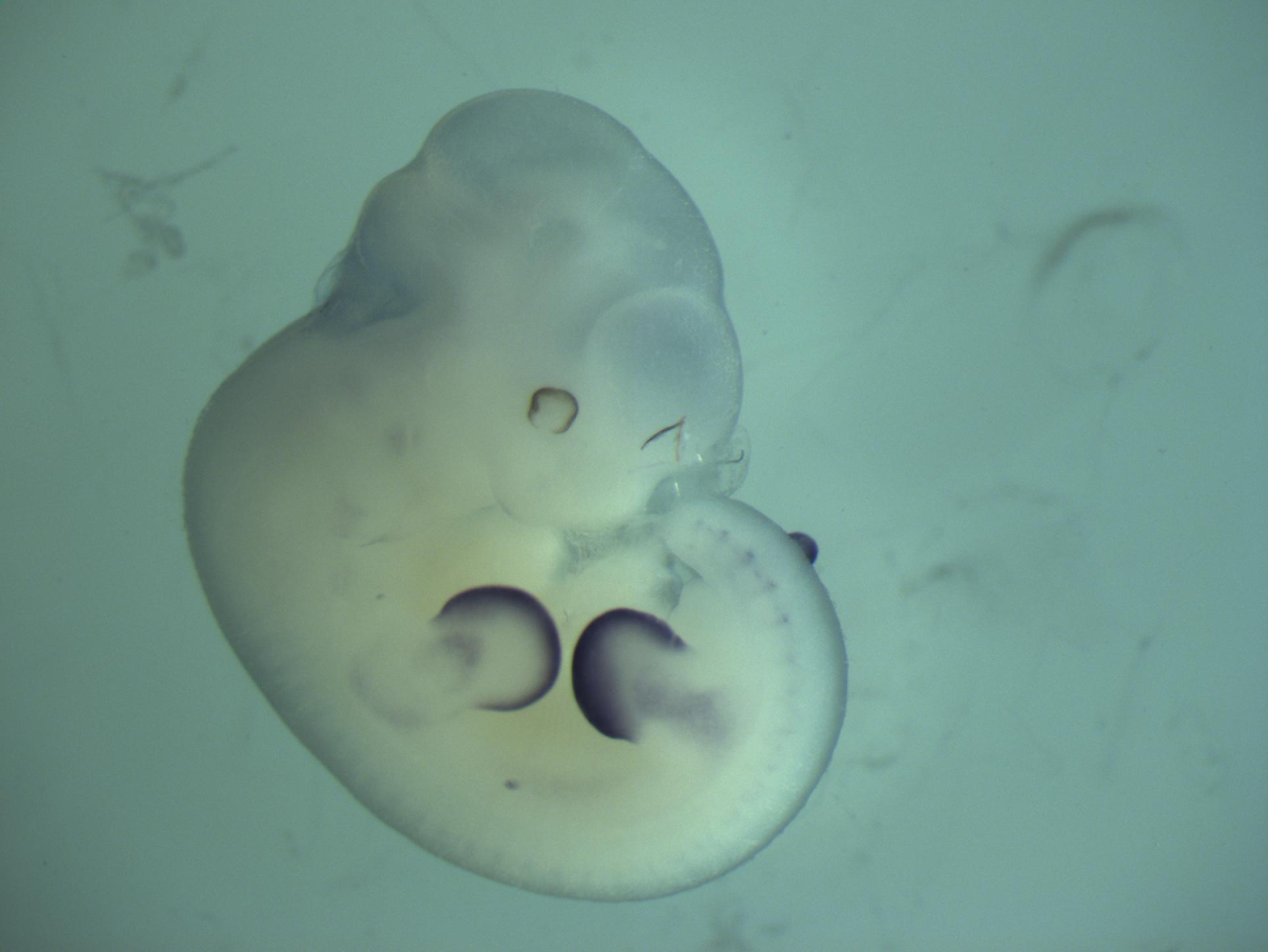

Supplement: Figure 2—source data 1. — This zip archive contains pictures, taken using a Leica MX16F microscope, of the right and left sides of the mouse embryos that underwent Dusp6 WMISH. Folders are organized by developmental stage and genotype. [file elife-36405-fig2-data1.zip › Figure 2 supplement 1-Source data 1/Dusp6 11.5 mut/Dusp6 11.5 mut7R.jpg]

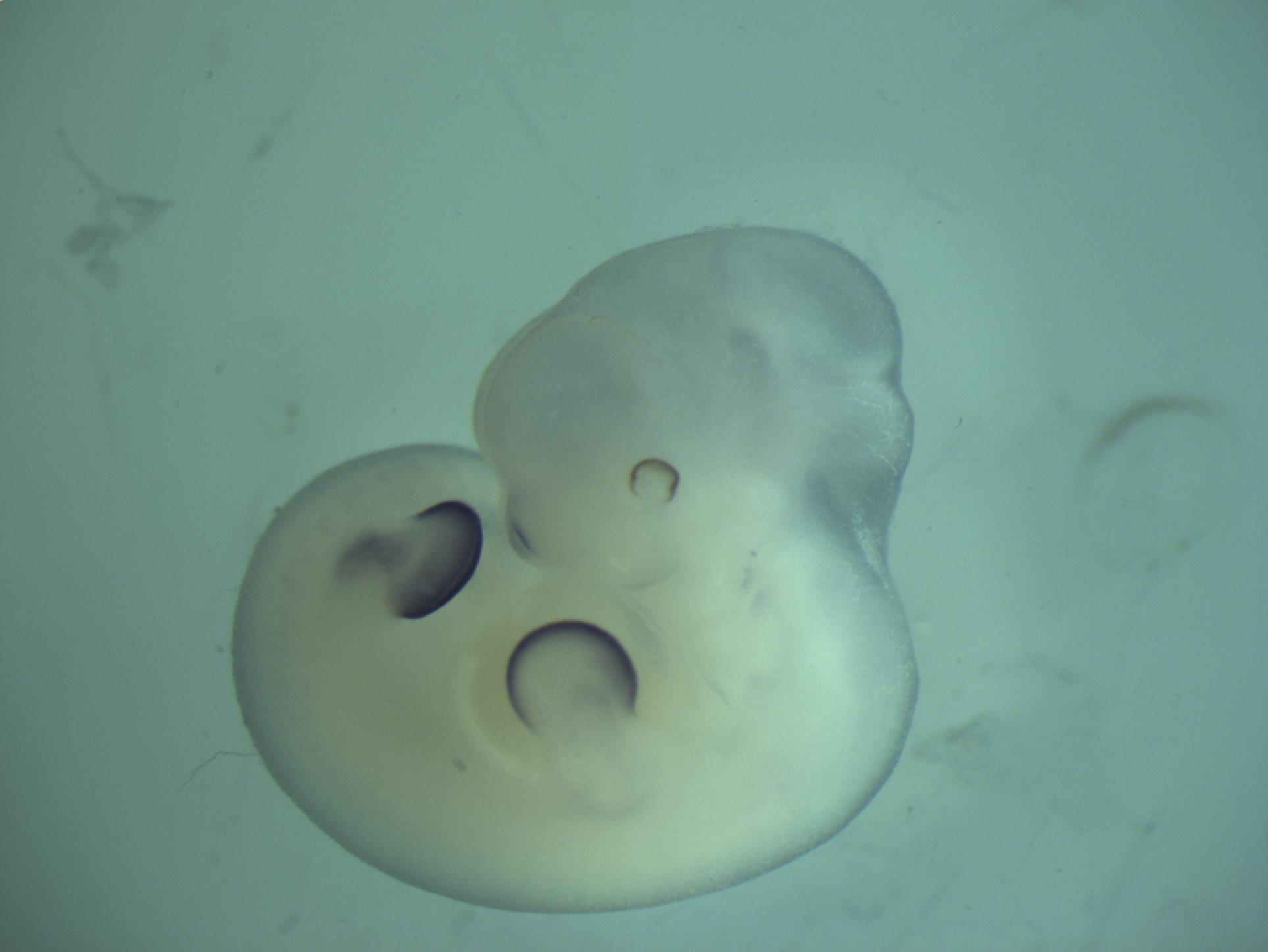

Supplement: Figure 2—source data 1. — This zip archive contains pictures, taken using a Leica MX16F microscope, of the right and left sides of the mouse embryos that underwent Dusp6 WMISH. Folders are organized by developmental stage and genotype. [file elife-36405-fig2-data1.zip › Figure 2 supplement 1-Source data 1/Dusp6 11.5 mut/Dusp6 11.5 mut8L.jpg]

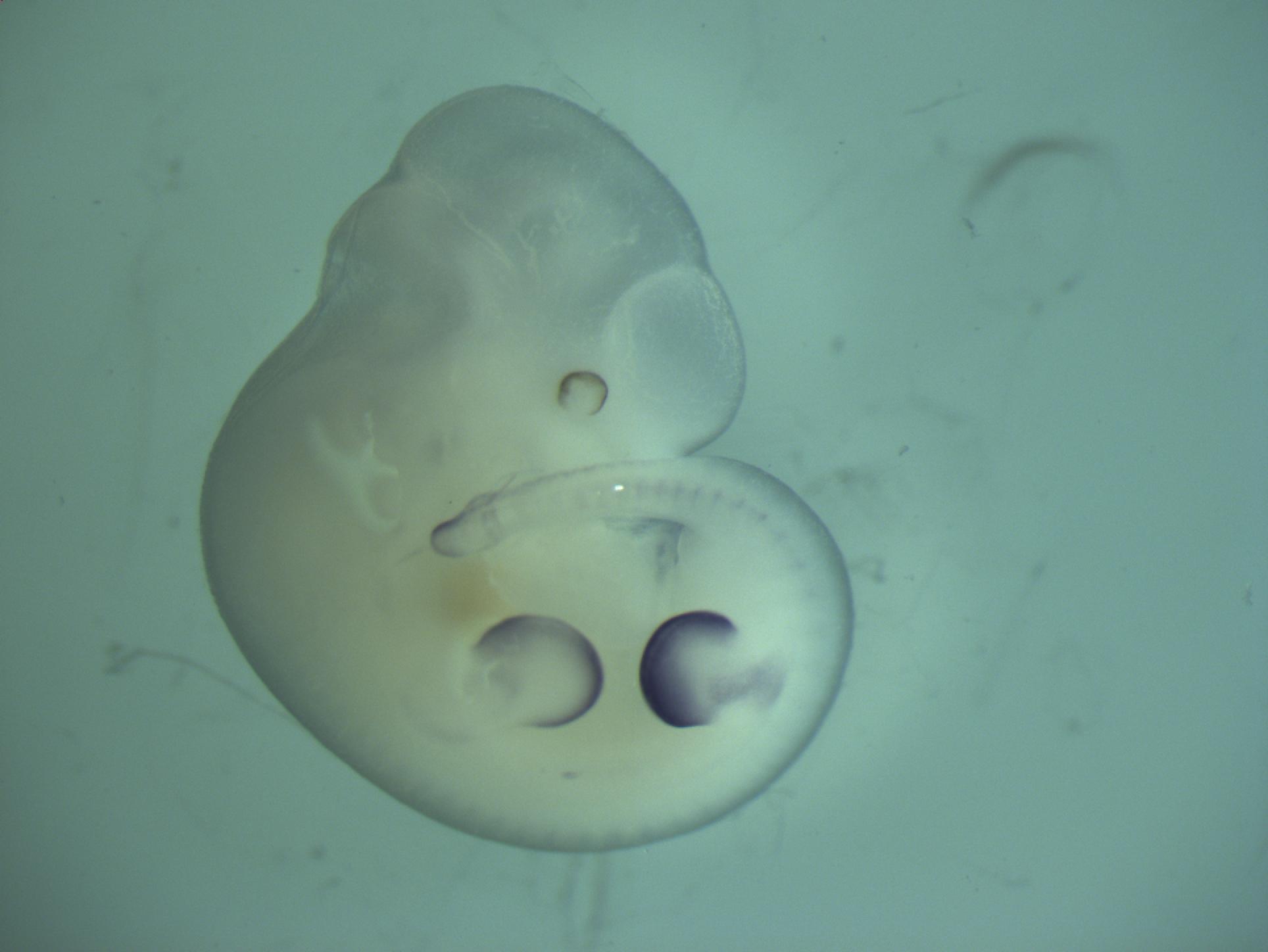

Supplement: Figure 2—source data 1. — This zip archive contains pictures, taken using a Leica MX16F microscope, of the right and left sides of the mouse embryos that underwent Dusp6 WMISH. Folders are organized by developmental stage and genotype. [file elife-36405-fig2-data1.zip › Figure 2 supplement 1-Source data 1/Dusp6 11.5 mut/Dusp6 11.5 mut8R.jpg]

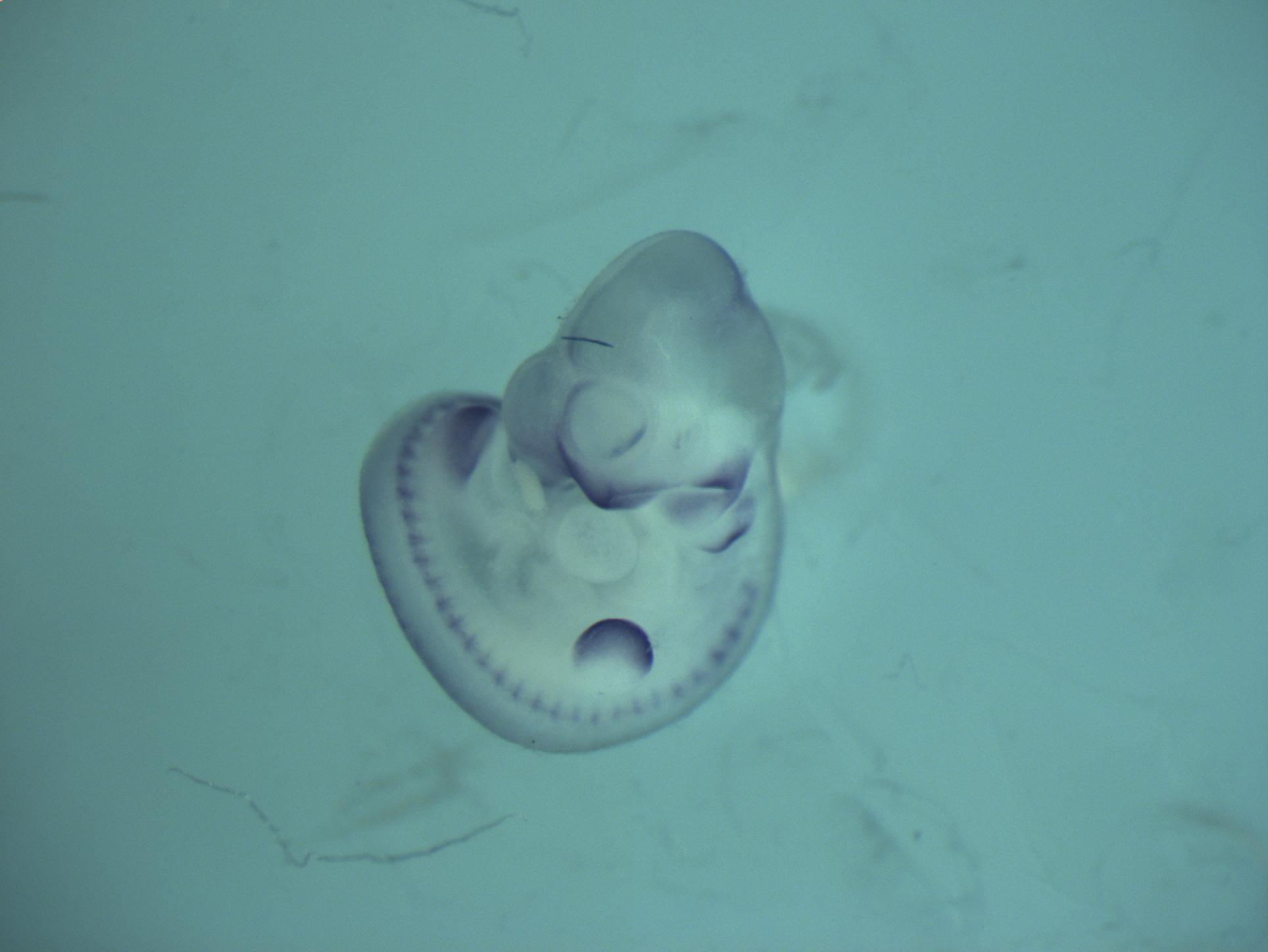

Supplement: Figure 2—source data 1. — This zip archive contains pictures, taken using a Leica MX16F microscope, of the right and left sides of the mouse embryos that underwent Dusp6 WMISH. Folders are organized by developmental stage and genotype. [file elife-36405-fig2-data1.zip › Figure 2 supplement 1-Source data 1/Dusp6 11.5 wt/Dusp6 11.5 wt1L.jpg]

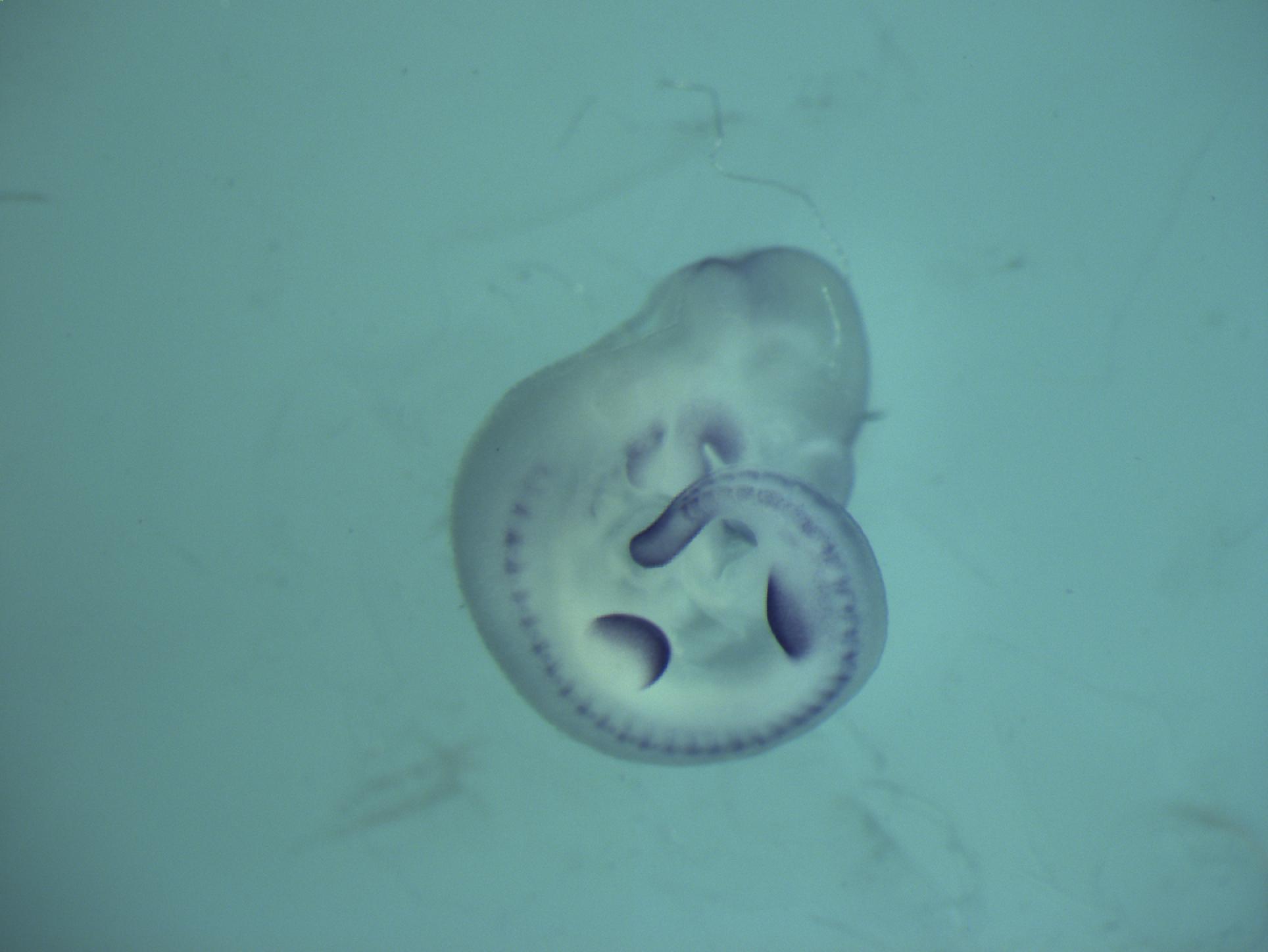

Supplement: Figure 2—source data 1. — This zip archive contains pictures, taken using a Leica MX16F microscope, of the right and left sides of the mouse embryos that underwent Dusp6 WMISH. Folders are organized by developmental stage and genotype. [file elife-36405-fig2-data1.zip › Figure 2 supplement 1-Source data 1/Dusp6 11.5 wt/Dusp6 11.5 wt1R.jpg]

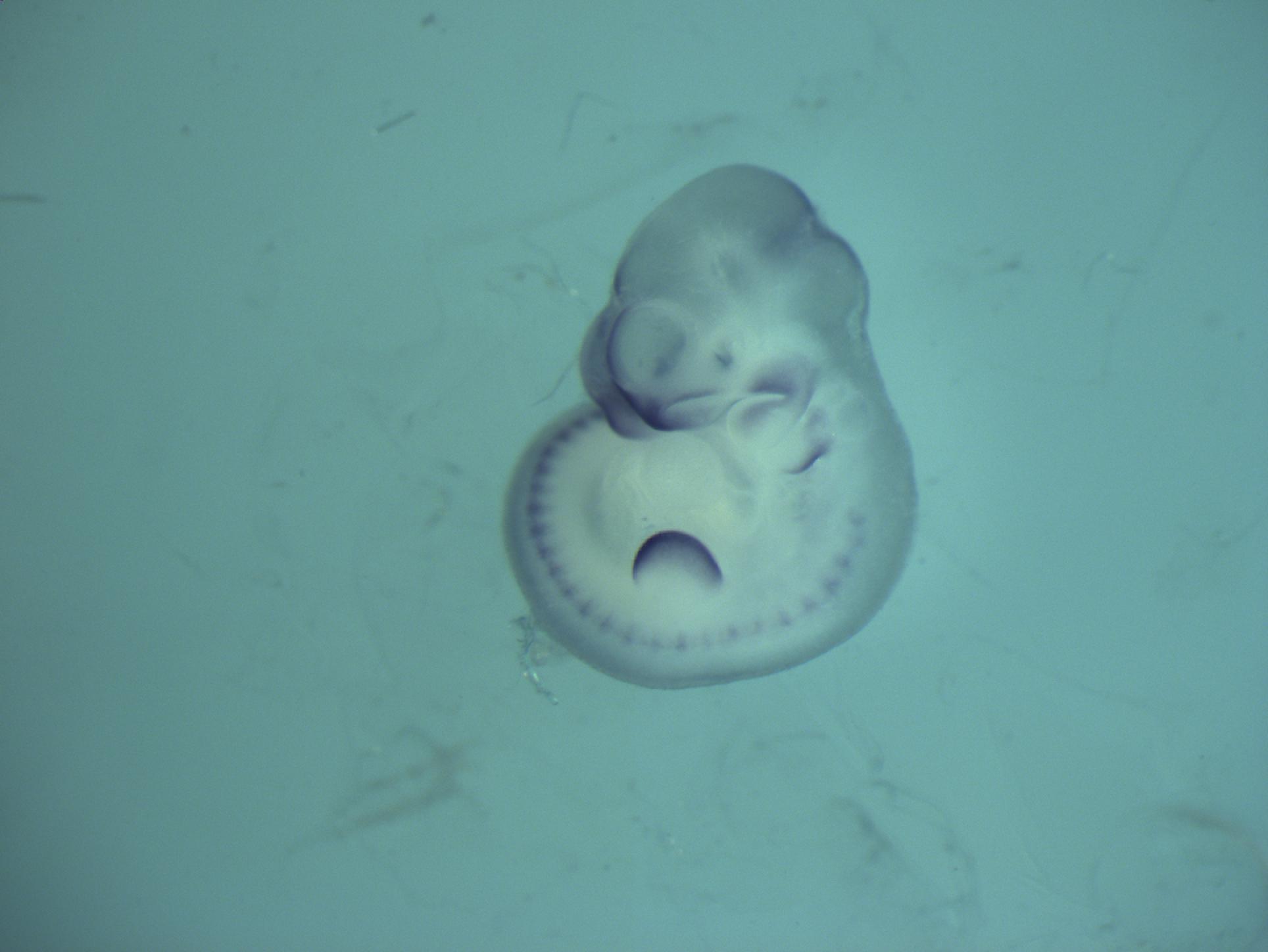

Supplement: Figure 2—source data 1. — This zip archive contains pictures, taken using a Leica MX16F microscope, of the right and left sides of the mouse embryos that underwent Dusp6 WMISH. Folders are organized by developmental stage and genotype. [file elife-36405-fig2-data1.zip › Figure 2 supplement 1-Source data 1/Dusp6 11.5 wt/Dusp6 11.5 wt2L.jpg]

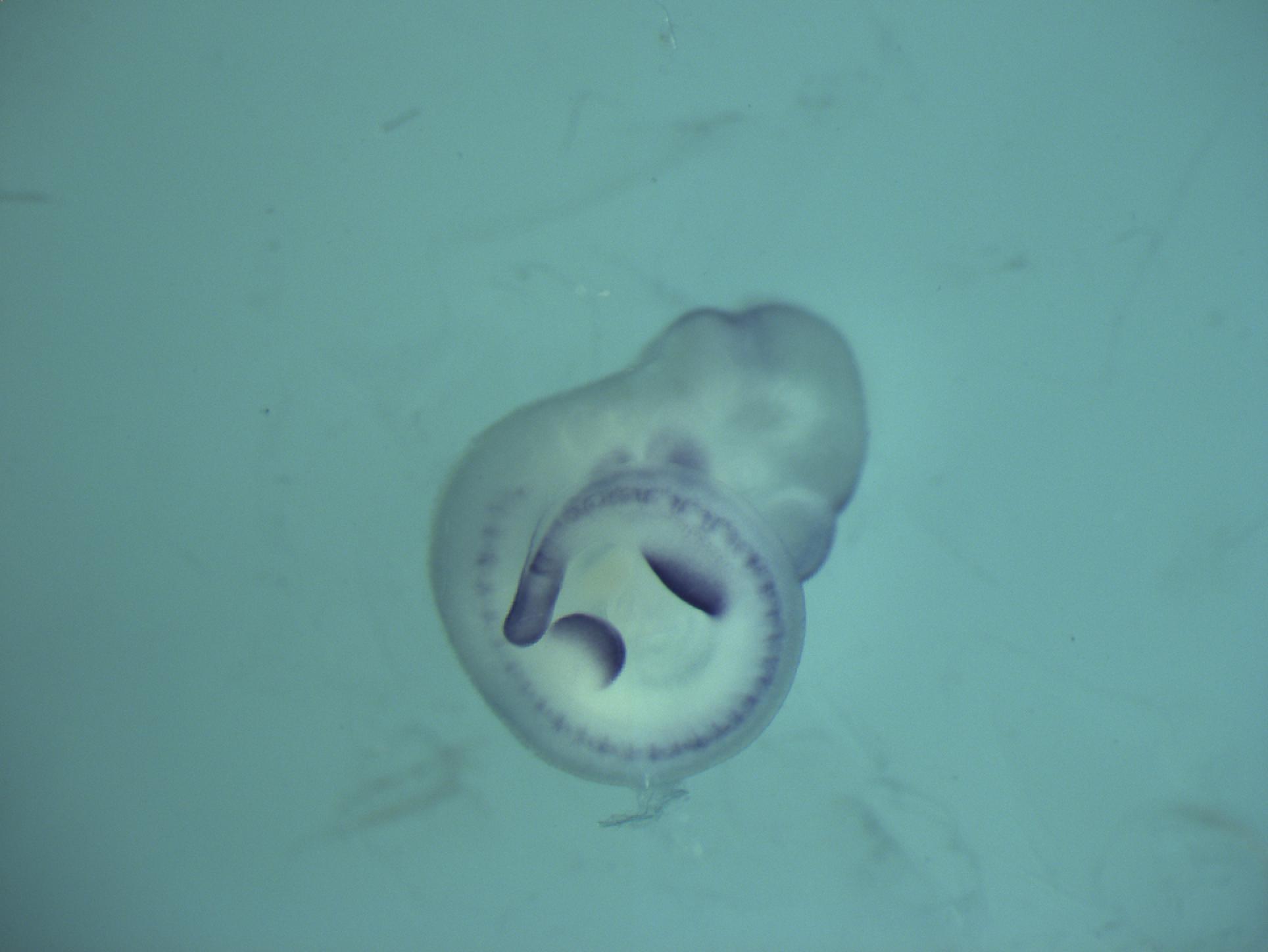

Supplement: Figure 2—source data 1. — This zip archive contains pictures, taken using a Leica MX16F microscope, of the right and left sides of the mouse embryos that underwent Dusp6 WMISH. Folders are organized by developmental stage and genotype. [file elife-36405-fig2-data1.zip › Figure 2 supplement 1-Source data 1/Dusp6 11.5 wt/Dusp6 11.5 wt2R.jpg]

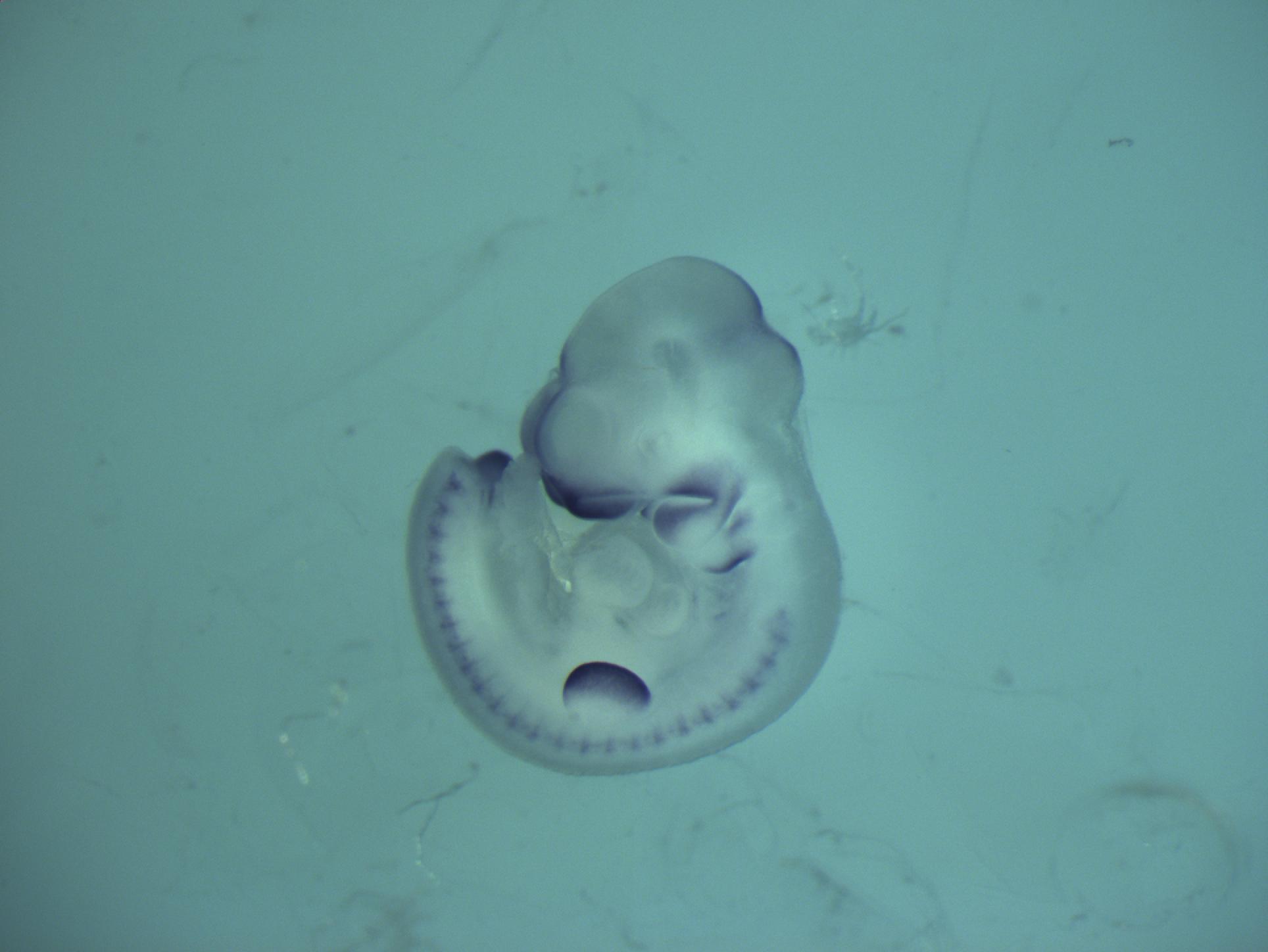

Supplement: Figure 2—source data 1. — This zip archive contains pictures, taken using a Leica MX16F microscope, of the right and left sides of the mouse embryos that underwent Dusp6 WMISH. Folders are organized by developmental stage and genotype. [file elife-36405-fig2-data1.zip › Figure 2 supplement 1-Source data 1/Dusp6 11.5 wt/Dusp6 11.5 wt3L.jpg]

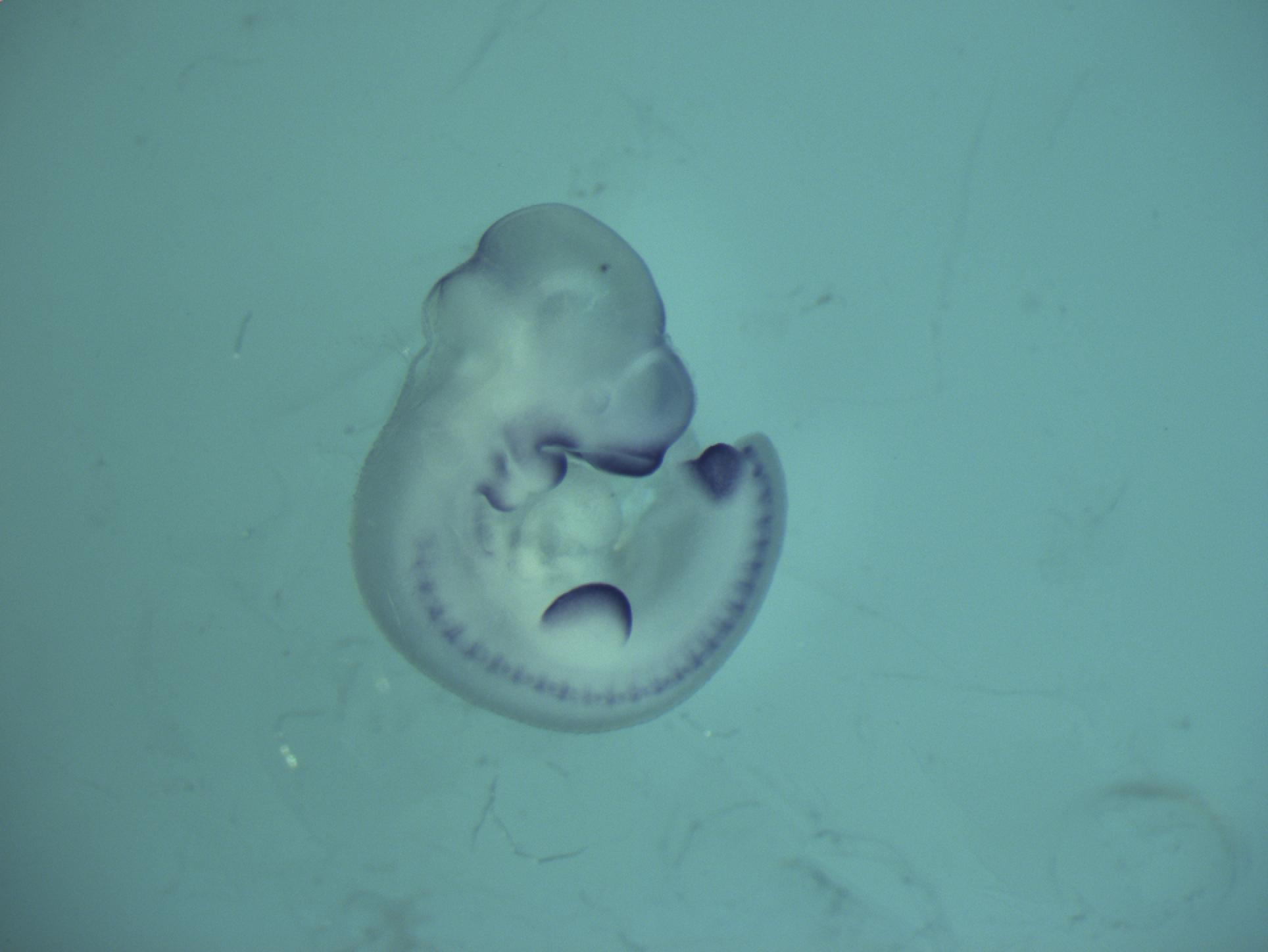

Supplement: Figure 2—source data 1. — This zip archive contains pictures, taken using a Leica MX16F microscope, of the right and left sides of the mouse embryos that underwent Dusp6 WMISH. Folders are organized by developmental stage and genotype. [file elife-36405-fig2-data1.zip › Figure 2 supplement 1-Source data 1/Dusp6 11.5 wt/Dusp6 11.5 wt3R.jpg]

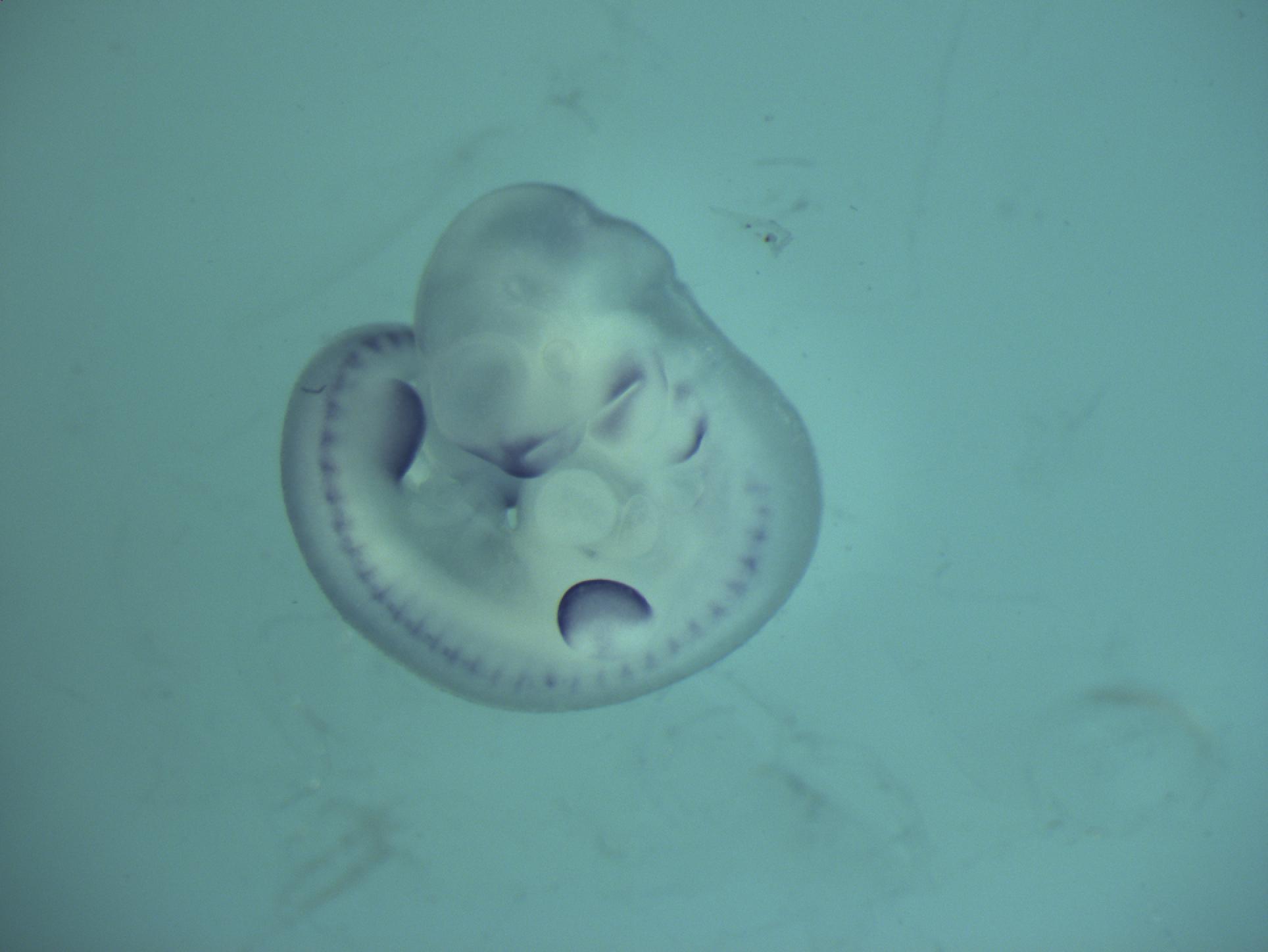

Supplement: Figure 2—source data 1. — This zip archive contains pictures, taken using a Leica MX16F microscope, of the right and left sides of the mouse embryos that underwent Dusp6 WMISH. Folders are organized by developmental stage and genotype. [file elife-36405-fig2-data1.zip › Figure 2 supplement 1-Source data 1/Dusp6 11.5 wt/Dusp6 11.5 wt4L.jpg]

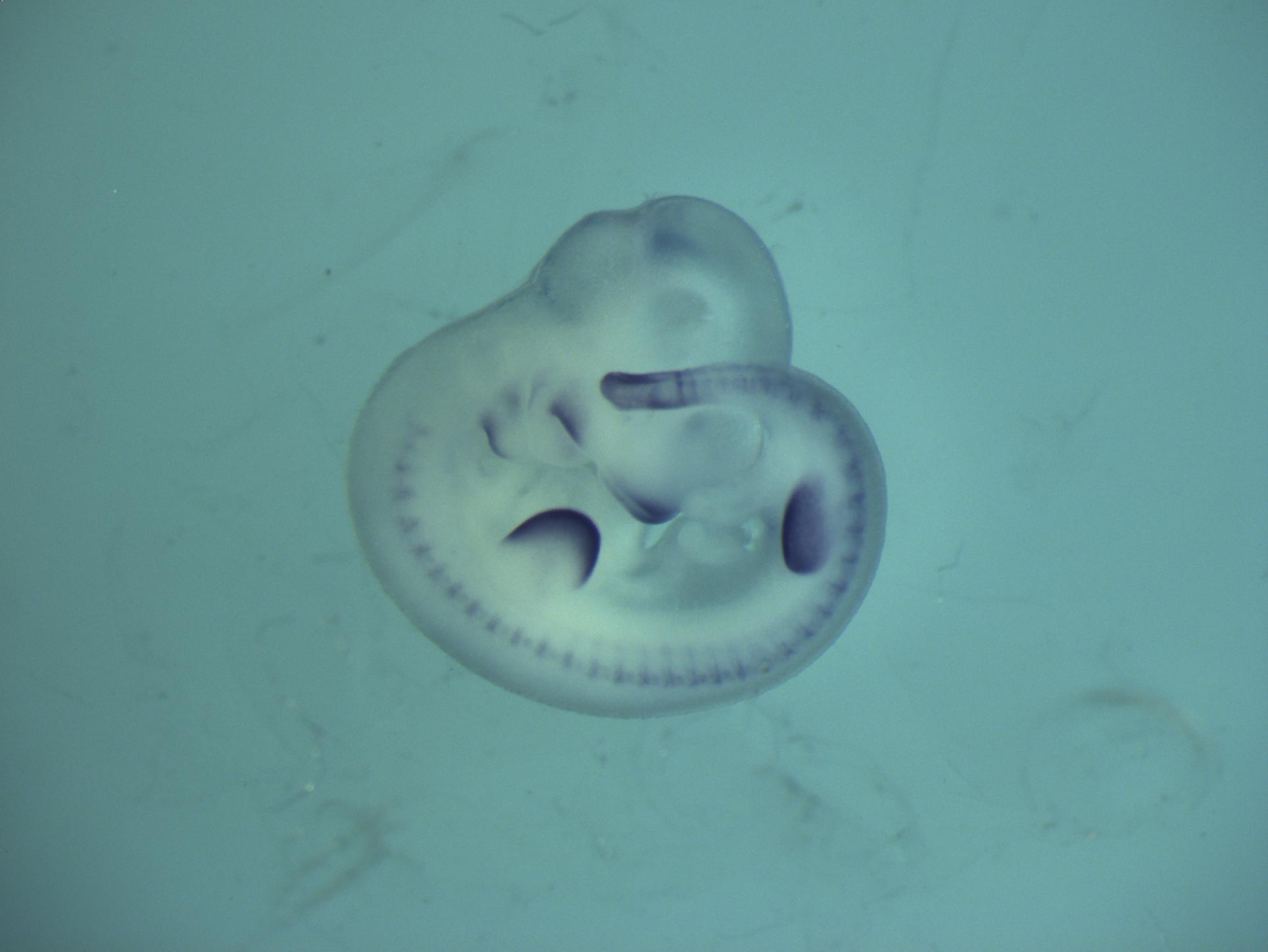

Supplement: Figure 2—source data 1. — This zip archive contains pictures, taken using a Leica MX16F microscope, of the right and left sides of the mouse embryos that underwent Dusp6 WMISH. Folders are organized by developmental stage and genotype. [file elife-36405-fig2-data1.zip › Figure 2 supplement 1-Source data 1/Dusp6 11.5 wt/Dusp6 11.5 wt4R.jpg]

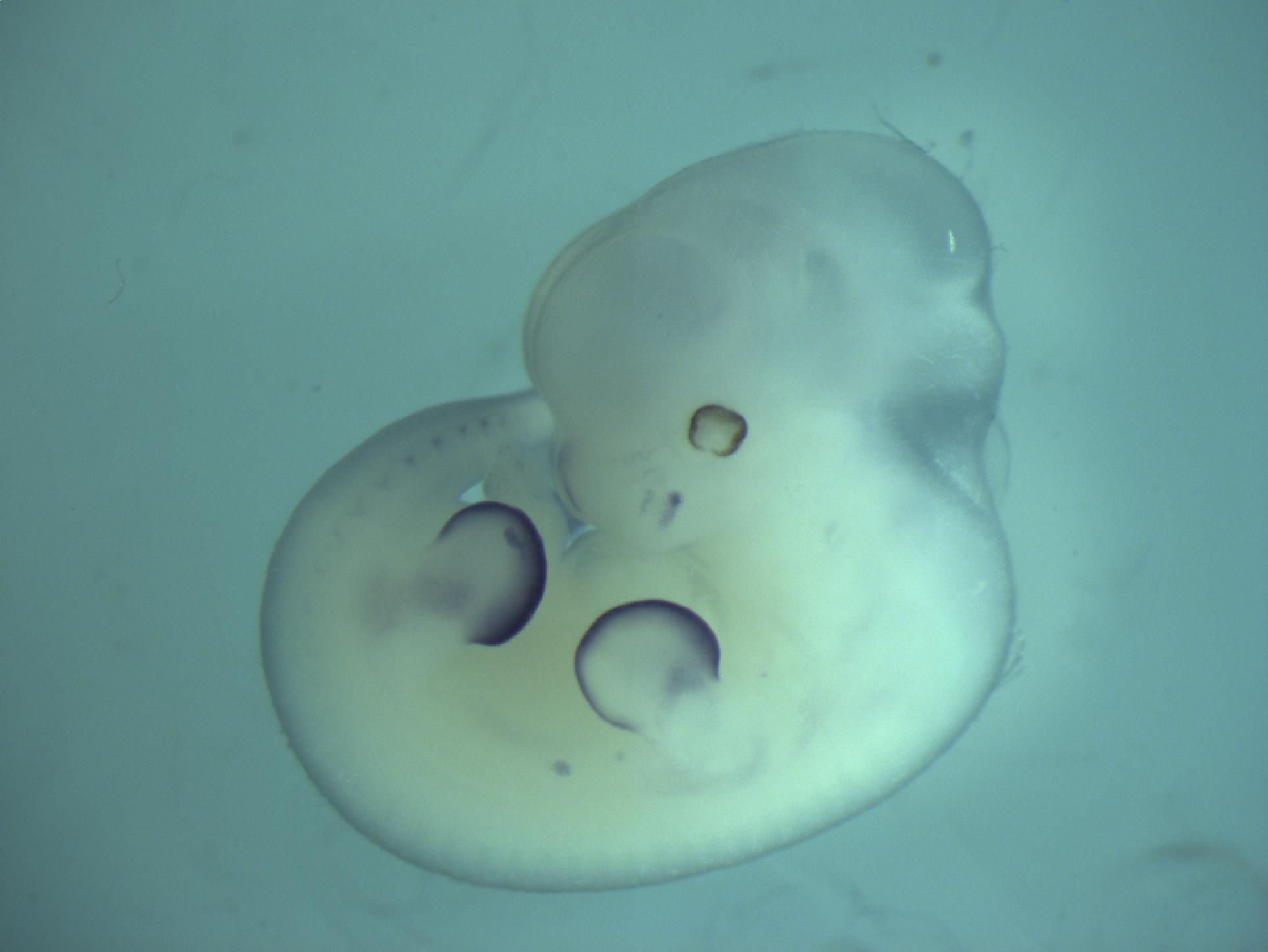

Supplement: Figure 2—source data 1. — This zip archive contains pictures, taken using a Leica MX16F microscope, of the right and left sides of the mouse embryos that underwent Dusp6 WMISH. Folders are organized by developmental stage and genotype. [file elife-36405-fig2-data1.zip › Figure 2 supplement 1-Source data 1/Dusp6 11.5 wt/Dusp6 11.5 wt5L.jpg]

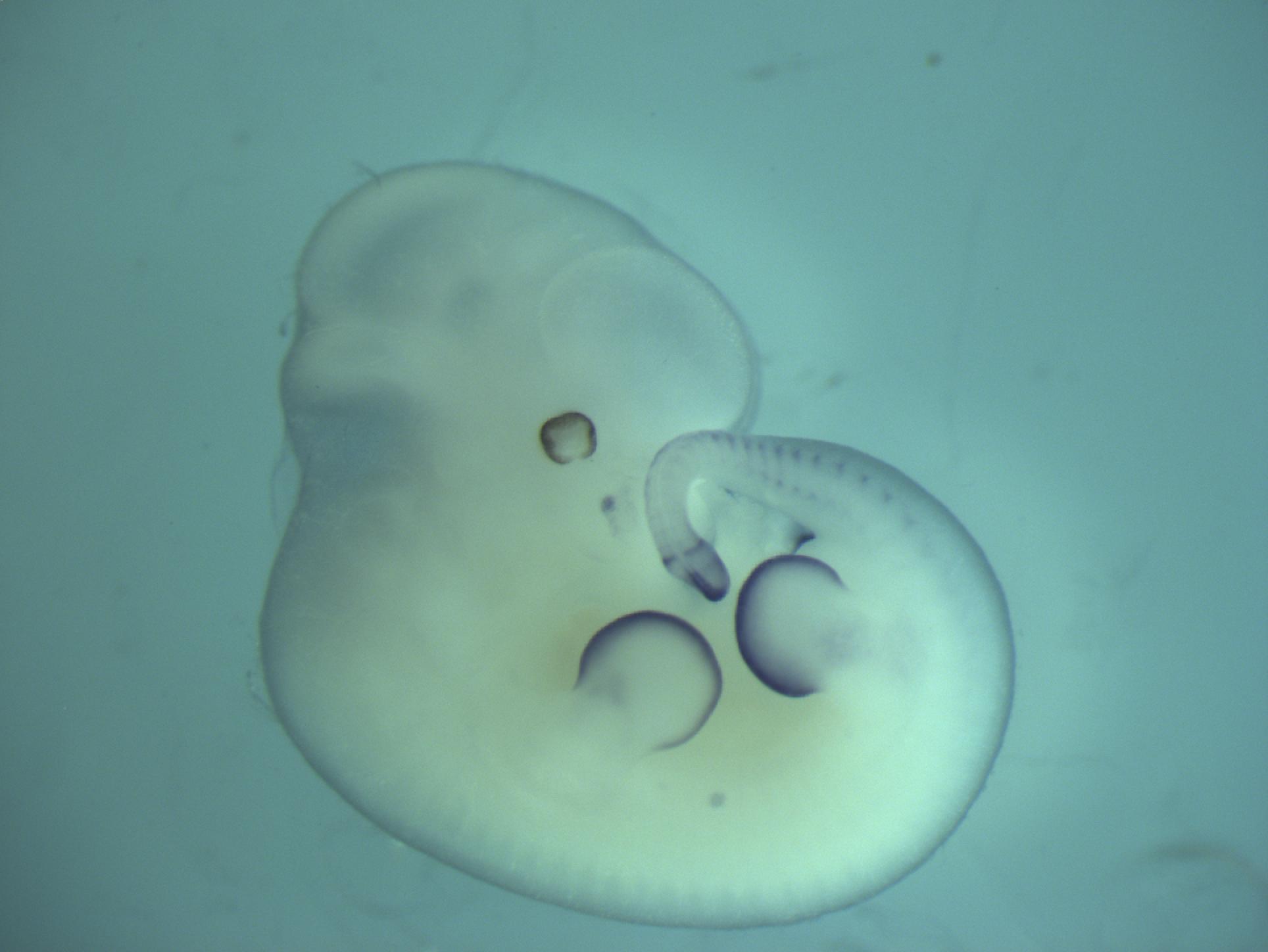

Supplement: Figure 2—source data 1. — This zip archive contains pictures, taken using a Leica MX16F microscope, of the right and left sides of the mouse embryos that underwent Dusp6 WMISH. Folders are organized by developmental stage and genotype. [file elife-36405-fig2-data1.zip › Figure 2 supplement 1-Source data 1/Dusp6 11.5 wt/Dusp6 11.5 wt5R.jpg]

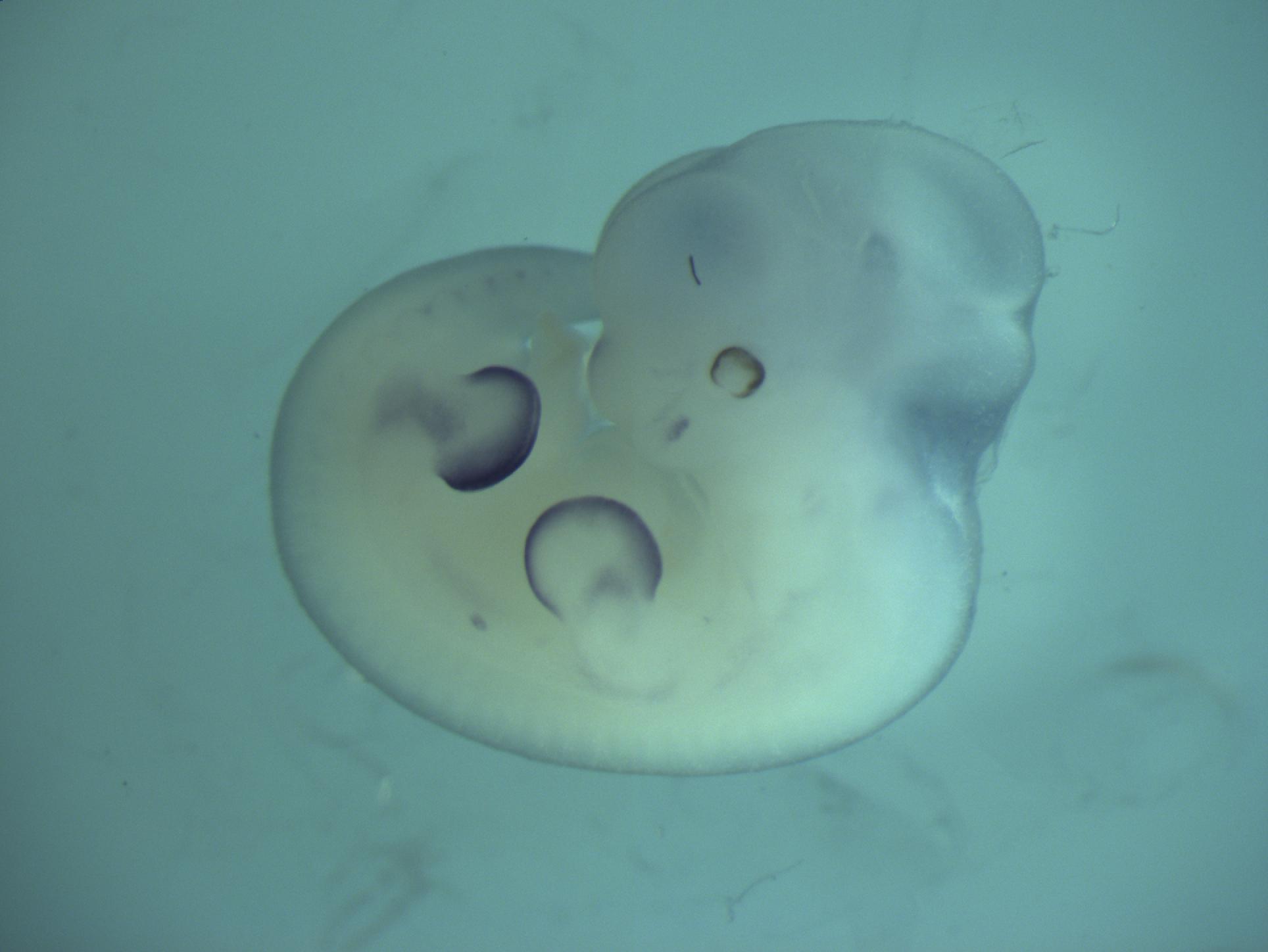

Supplement: Figure 2—source data 1. — This zip archive contains pictures, taken using a Leica MX16F microscope, of the right and left sides of the mouse embryos that underwent Dusp6 WMISH. Folders are organized by developmental stage and genotype. [file elife-36405-fig2-data1.zip › Figure 2 supplement 1-Source data 1/Dusp6 11.5 wt/Dusp6 11.5 wt6L.jpg]

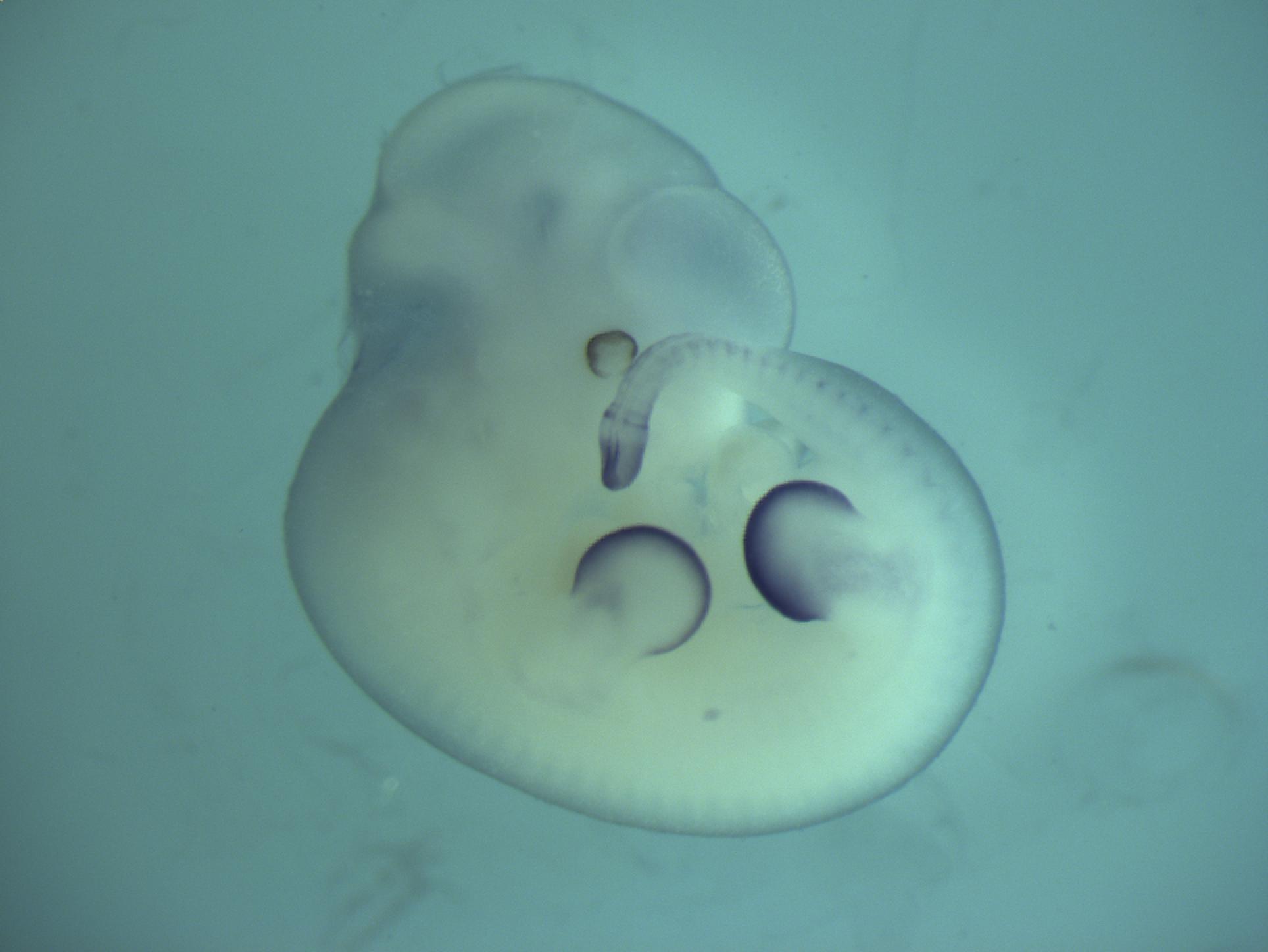

Supplement: Figure 2—source data 1. — This zip archive contains pictures, taken using a Leica MX16F microscope, of the right and left sides of the mouse embryos that underwent Dusp6 WMISH. Folders are organized by developmental stage and genotype. [file elife-36405-fig2-data1.zip › Figure 2 supplement 1-Source data 1/Dusp6 11.5 wt/Dusp6 11.5 wt6R.jpg]
